# Programming intramolecular charge transfer in NDI–pyrene paracyclophanes through distance and linker design

**DOI:** 10.1039/d6tc02775j

**Published:** 2026-10-21

**Authors:** Camila Negrete-Vergara, Vladislav Slama, Elnaz Zyaee, Hans-Martin Frey, Silvio Decurtins, Thomas Feurer, Ursula Röthlisberger, Shi-Xia Liu

**Affiliations:** a Department of Chemistry, Biochemistry and Pharmaceutical Sciences, W. Inäbnit Laboratory for Molecular Quantum Materials and WSS-Research Center for Molecular Quantum Systems, University of Bern Freiestrasse 3 Bern 3012 Switzerland shi-xia.liu@unibe.ch; b Laboratory of Computational Chemistry and Biochemistry, Institute of Chemical Sciences and Engineering, École Polytechnique Fédérale de Lausanne Lausanne 1015 Switzerland ursula.roethlisberger@epfl.ch; c Institute of Applied Physics, University of Bern Sidlerstrasse 5 Bern 3012 Switzerland

## Abstract

Through-space charge transfer (TSCT) in electron donor–acceptor (D–A) molecules is a powerful strategy for controlling excited-state processes. However, the influence of molecular architecture on TSCT dynamics remains poorly understood. Herein, we report a series of naphthalene diimide (NDI)-pyrene (Py) D–A paracyclophanes (PCPs) with systematically varied interchromophore distance and spatial arrangement *via* different linkers: phenylene (NDI-Ph-Py), benzylamine (NDI-BnNH-Py), and adamantylamine (NDI-AdaNH-Py). All PCPs exhibit low-energy CT absorption bands absent in the corresponding reference compounds, confirming efficient intramolecular TSCT interactions. Combined spectroscopic, electrochemical, and computational studies reveal that the TSCT strength and energies are highly sensitive to linker structure, rigidity, and NDI–Py separation. Furthermore, femtosecond transient absorption spectroscopy shows multiple CT states and distinct relaxation pathways governed by linker properties. NDI-AdaNH-Py exhibits CT-state formation on the sub-picosecond timescale, facilitated by the short NDI–Py separation distance and substantial orbital overlap. NDI-Ph-Py, with the shortest interchromophore distance, strongest electronic coupling, and rigid structure, further promotes the formation of an exciplex state. These findings demonstrate that linker engineering is an effective means of modulating TSCT interactions, providing valuable design principles for the development of next-generation functional organic materials.

## Abstract 2

The molecular geometry controls the through-space charge transfer (TSCT) by adjusting the distance between chromophores and the overlap of their orbitals. Shorter distances between the NDI and pyrene enable rapid CT and efficient exciplex formation.

## Graphical abstract

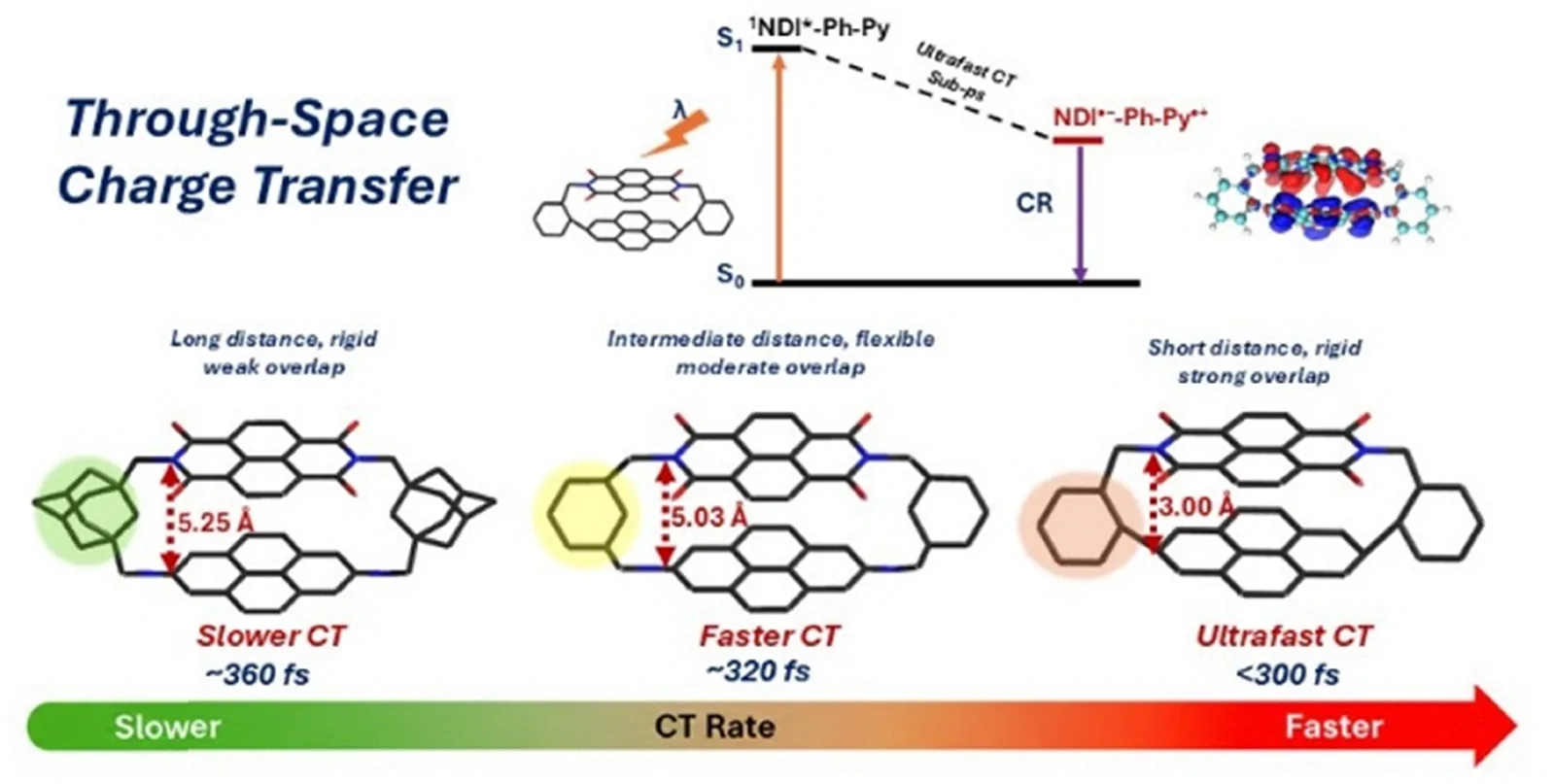

## Introduction

Understanding charge transport and charge transfer (CT) phenomena in organic electron donor–acceptor (D–A) systems is fundamental to the rational design of molecular electronic and optoelectronic devices.^1–7^ The efficiency of the CT state depends on the strength of the electronic coupling between the D and A moieties, which is primarily determined by the overlap of their frontier molecular orbitals, namely the HOMO and LUMO. An effective strategy for enhancing D–A coupling is to covalently link the D and A chromophores *via* π-conjugated bridges.^3^ While this approach promotes efficient electronic delocalization, particularly in fused D–A ensembles, it can also lead to significant mixing of the respective HOMO and LUMO levels, which diminishes their effectiveness as strong donors or strong acceptors by increasing the HOMO–LUMO gap.^8^ As orbital overlap is highly sensitive to interchromophore distance and orientation, the spatial organization imposed by different types of linkers becomes a key factor in governing CT rates and excited-state energetics. Consequently, through-space charge transfer (TSCT) emerges as an appealing alternative, enabling effective electronic interaction through π-orbital overlap. Despite this potential, the structure–property relationships of TSCT-based optoelectronic materials are far less well understood than those of their through-bond charge transfer (TBCT) counterparts, likely because identifying, precisely controlling, and characterizing noncovalent interactions is challenging.^9^

Paracyclophane (PCP) is a versatile scaffold that could provide both conformational rigidity and chirality.^10–21^ Replacing the PCP's benzene rings with D and A moieties can create a well-defined chiral environment, enabling the study of chiroptical materials^22^ and the engineering of π–π interactions between the aromatic chromophores.^23–25^ In this context, the extent of orbital overlap between π-stacked aromatic D and A units largely governs TSCT, depending critically on their distance and relative orientation, as well as the conformational constraints imposed by the bridge.^22,24,26^ However, systematic investigations of TSCT dynamics in hetero cyclophane systems remain scarce, and to date, only a few reports have addressed their TSCT dynamics.^22,24^ One study focused on naphthalene diimide (NDI)-based PCPs that incorporate electron donors, such as carbazole, benzodithiophene, and dihydroindole.^22^ The authors demonstrated that enforced D–A π-stacking promotes efficient non-radiative decay and photothermal conversion, as evidenced by femtosecond transient absorption spectroscopy. Another study explored an asymmetric perylene diimide-based PCP,^24^ showing that rapid charge separation arises from TSCT, while the significantly prolonged lifetime of the charge-separated (CS) state is due to charge transport through intermolecular π-stacks, which is advantageous for solar energy conversion.

The combination of NDI and Py is a well-established motif in CT materials, including supramolecular polymers,^27–29^ single cocrystals and complexes,^30–32^ as well as PCPs.^33^ Notably, only one example of an NDI–Py cyclophane fixed by ethylene bridges has been reported to date. This molecule exhibited a broad absorption band centred at 520 nm (*ε* = 1010 cm^−1^ M^−1^ in CHCl_3_), which is attributed to a TSCT transition arising from a face-to-face arrangement. Single-crystal X-ray diffraction revealed an interchromophore separation of approximately 3.5 Å.^33^ Nevertheless, a comprehensive investigation into how bridge identity and D–A distance influence TSCT and excited-state dynamics in these systems is lacking.

To address this gap, we report a new family of NDI–Py PCPs in which D and A moieties are connected by phenylene, benzylamine, or adamantylamine bridges (Scheme 1). The Py core is selectively functionalized at the 2,7-positions with bromine and boronic ester groups, enabling C–N Buchwald coupling and Suzuki–Miyaura coupling, respectively. This strategy allows for either direct connection to phenylene linkers or connection *via* an amine (NH) group to benzyl or adamantane linkers. These bridge structures impose different levels of rigidity and interchromophore separation, which allows us to evaluate distance- and bridge-dependent electronic properties using a combination of steady-state UV-vis absorption spectroscopy and cyclic voltammetry measurements. All experimental findings are further verified by extensive quantum chemical calculations. Importantly, time-resolved transient absorption spectroscopy reveals intricate dynamics, emphasizing the crucial role of bridge-controlled spatial proximity in regulating intramolecular TSCT dynamics.

**Scheme 1:**
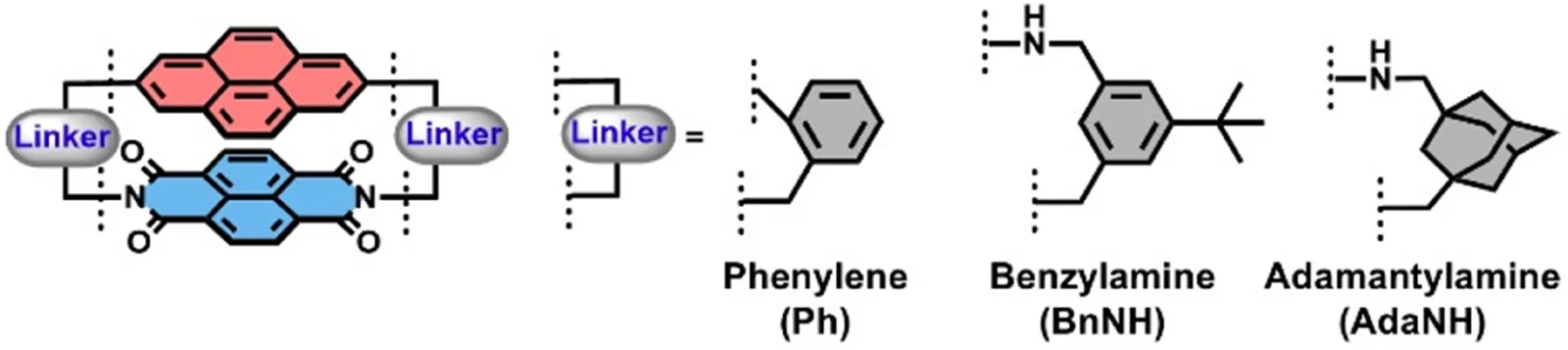
Schematic representation of the different PCP structures investigated in this work.

## Results and discussion

### Synthesis and characterization

The cyclophanes cyclization was achieved *via* the condensation of naphthalenetetracarboxylic dianhydride (NTDA) with the corresponding deprotected amine (AdaNH-Py, BnNH-Py, and PhNH-Py) in the presence of triethylamine (Et_3_N) in dilute conditions (*ca.* 1 mM), as illustrated in Scheme 2. The inherently low yields of these reactions are primarily attributed to the unavoidable formation of polymeric byproducts.^34^ The 2,7-substituted pyrene building blocks were synthesized through palladium-catalyzed cross-coupling reactions: C–N Buchwald coupling for AdaNHBoc-Py and BnNHBoc-Py, and Suzuki–Miyaura coupling for the PhNHBoc-Py scaffold. All PCPs underwent extensive purification, including column chromatography, gel permeation chromatography, and, in some cases, high-temperature vacuum treatment to eliminate trace impurities. The structures of the resulting cyclophanes and reference compounds were unambiguously confirmed by ^1^H and ^13^C NMR spectroscopy, as well as high-resolution mass spectrometry. Notably, as shown in Fig. 1 and Fig. S1–S3, ^1^H NMR measurements revealed that the aromatic protons of the NDI and Py units in the D–A PCPs are significantly upfield shifted compared to their respective monomers. For example, the ^1^H NMR signals of the NDI moiety in NDI-BnNH-Py PCP (8.06 ppm) were significantly upfield shifted compared to those of the NDI monomer (8.68 ppm). Similarly, the protons of the BnNHBoc-Py monomer (7.79 and 7.38 ppm) were also significantly shifted compared to those of the D–A PCP (7.29 and 7.02 ppm). These findings indicate strong π–π interactions, even in solution. Additionally, NDI and Py protons in all D–A PCPs appear as single peaks, revealing the symmetric nature of these systems.

**Scheme 2:**
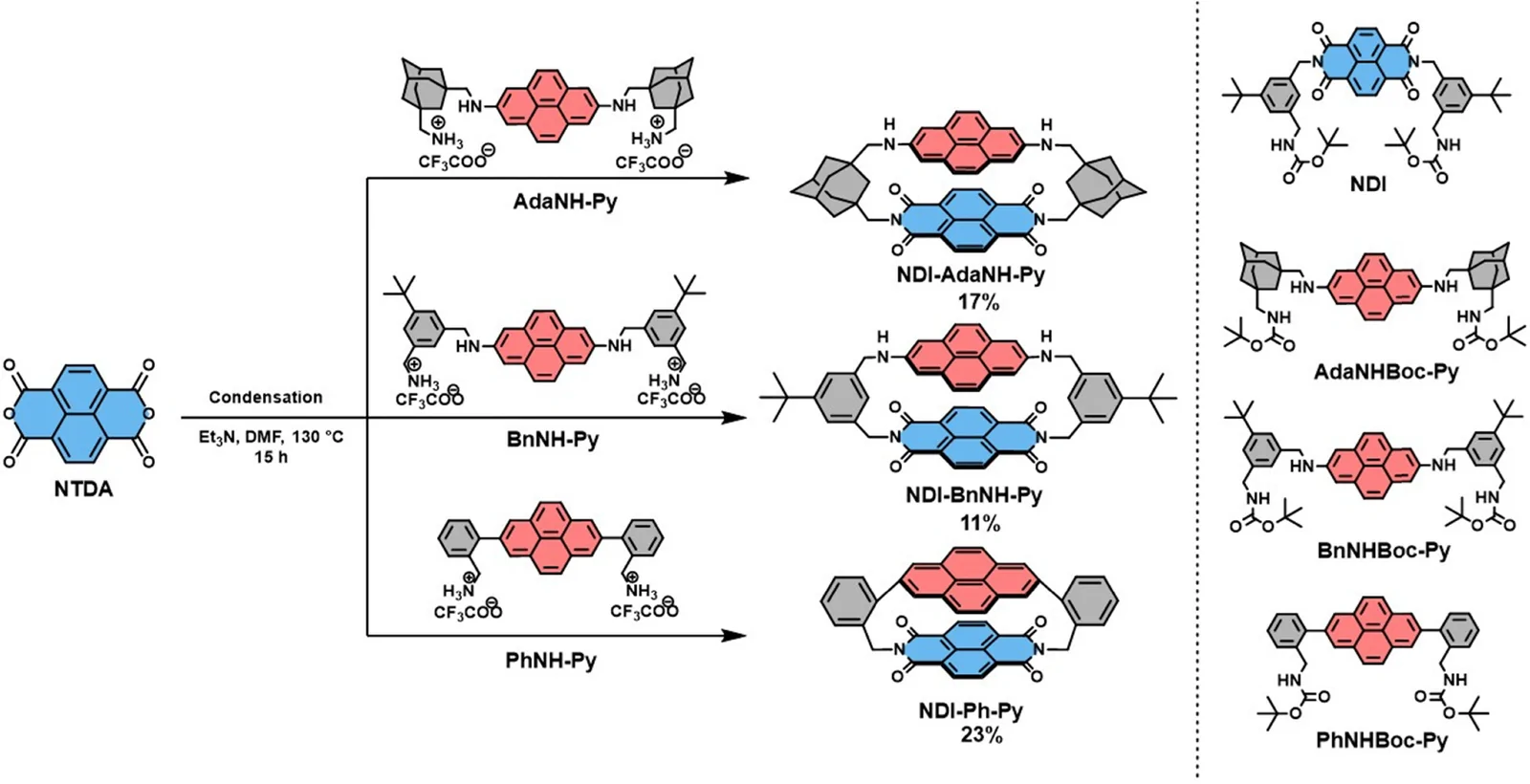
Synthetic routes to NDI-AdaNH-Py, NDI-BnNH-Py, and NDI-Ph-Py cyclophanes and structures of their reference molecules (NDI, AdaNHBoc-Py, BnNHBoc-Py, and PhNHBoc-Py).

**Fig. 1:**
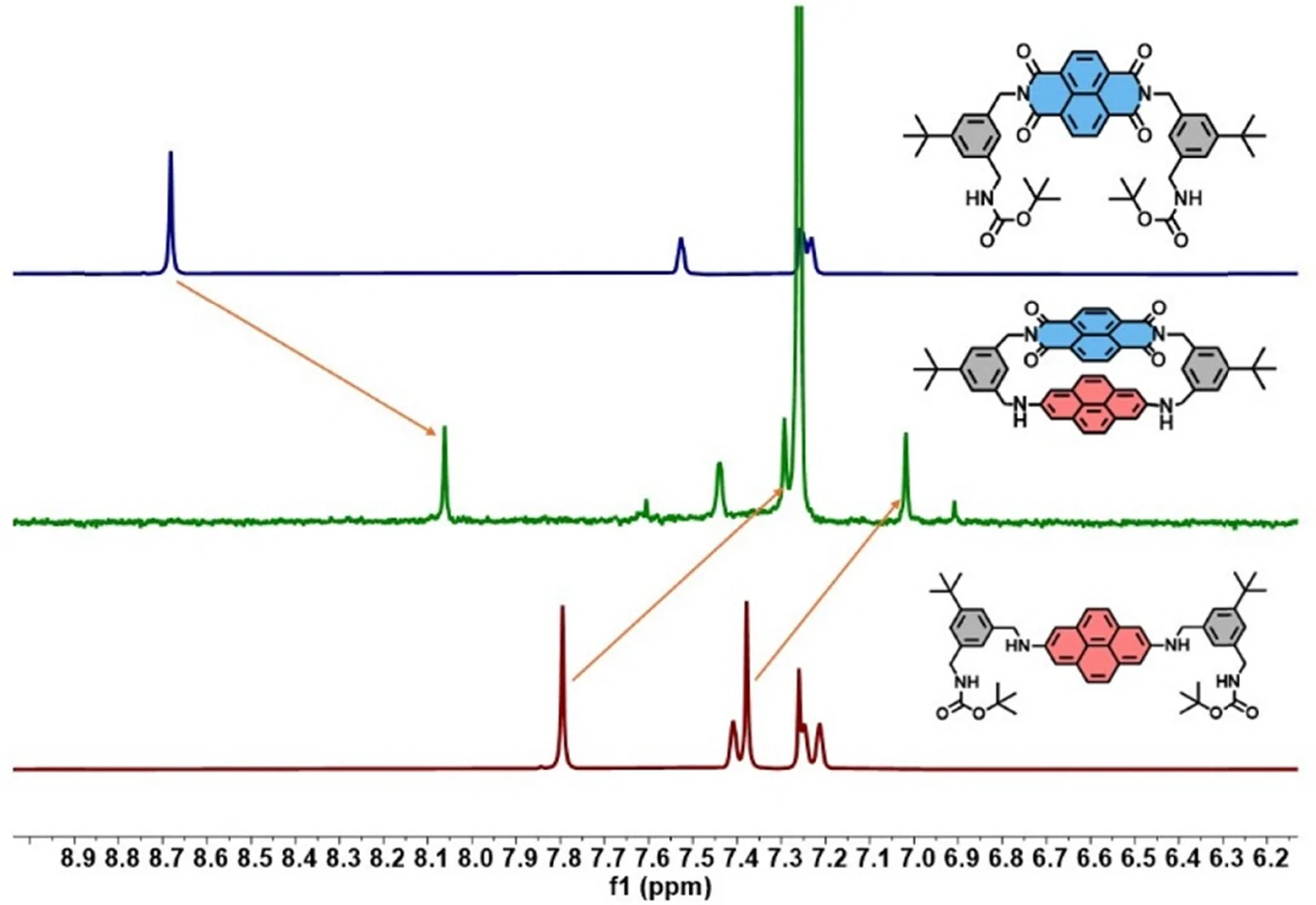
Partial ^1^H NMR spectra of NDI reference, NDI-BnNH-Py, and BnNHBoc-Py reference in CDCl_3_.

### Structure of the paracyclophanes

Since many attempts to crystallize the hetero paracyclophanes were unsuccessful, the optimized geometries were instead calculated using DFT with solvent effects modeled *via* the polarizable continuum model (PCM) approach in CH_2_Cl_2_.

Structural features depend on the linker type and the relative lengths of the two chromophore units. The BnNH linker introduces greater flexibility, allowing parallel stacking of slightly mismatched units, whereas the more rigid AdaNH linker causes a pronounced inclination between monomers. PCPs with amine groups can adopt cis or trans conformations, where amine hydrogens face the same or opposite directions, respectively. These conformers are nearly isoenergetic at the room temperature: for AdaNH-linked PCPs, the *cis* form is favored by 29.6 meV, while for BnNH-linked PCP, the *trans* form is slightly more stable by 38.3 meV (though *cis* is favored at 0 K by 13.6 meV). These small differences, comparable to thermal energy at room temperature (∼25.9 meV), suggest that both forms coexist. A length mismatch of ∼1.8 Å between the Py and NDI units leads to mutual rotation, allowing for accommodation of the geometry without excessive bending. Structures with *cis* amine groups show a greater inclination between the NDI and Py planes compared to *trans* structures. In NDI-Ph-Py with the shortest linker, the inter-unit distance near the linker is 3.0 Å, which is smaller than the sum of the van der Waals radii of two carbon atoms (∼3.4 Å). This leads to repulsion between the aromatic units, which bends the structure and increases the NDI–Py spacing at the center to ∼3.7 Å (see Fig. 2).

**Fig. 2:**
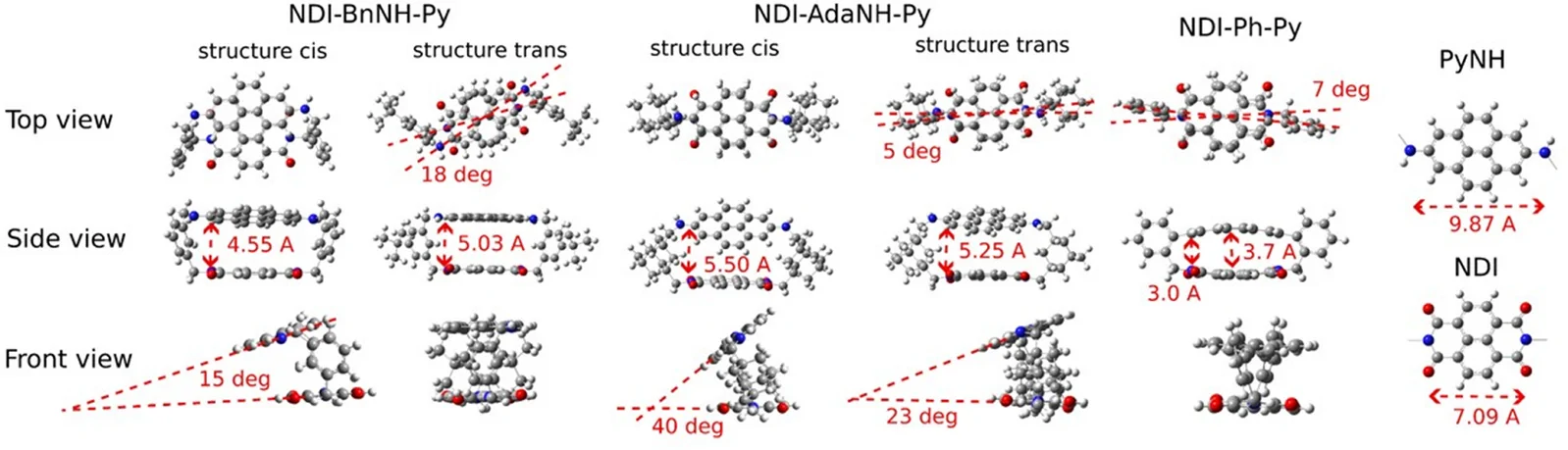
DFT-optimized geometries for the three D–A PCPs.

### Optical properties

The UV-vis absorption spectra of the different PCPs measured in CH_2_Cl_2_ are shown in Fig. 3 and summarized in Table 1, along with the calculated ones for comparison. For clarity, only the spectra of the energetically most favorable conformers are shown. The corresponding *cis*/*trans* conformers exhibit very similar optical properties with only small differences (Fig. S13 in SI). The combination of the electron-donating Py chromophore, especially conferred by the amine group at the 2,7 positions, with the electron-accepting NDI molecule could lead to the formation of several low-energy inter-chromophore (through-space) CT states. To calculate the excited state transitions, we employed a two-step computational procedure previously reported by us that provides high accuracy of the excited state properties and simulated optical spectra in exceptional agreement with the experimental ones (Fig. 3).^35^ In the blue and UV region of the optical spectra (230–400 nm), all D–A PCPs present intense absorption bands that can easily be ascribed to π–π* transitions of the individual NDI and Py chromophores. Remarkably, for NDI-Ph-Py, the distinct S_1_ transition of the NDI moiety around 380 nm exhibits a visible red shift (*ca.* 11 nm; see Table 1). In contrast, NDI-AdaNH-Py and NDI-BnNH-Py with larger separations between chromophores display more subtle effects when compared with their respective references, with only minor broadening and negligible shifts in the bands of the locally excited (LE) states. This is supported by theoretical calculations, which yield a coupling of 668 cm^−1^ between Py and NDI LE states for NDI-Ph-Py, which is more than 2.6 times larger than the one in NDI-AdaNH-Py (255 cm^−1^). Additionally, in the 400–500 nm region, the AdaNHBoc-Py and BnNHBoc-Py reference compounds exhibit a broad, relatively weak absorption band, previously ascribed to the short-axis polarized transition (^1^L_b_), which includes a significant contribution from intramolecular charge transfer (ICT) from the amine group to the Py. Similar ICT bands have been observed in other 2,7-diamine-substituted Py derivatives.^36–41^

**Fig. 3:**
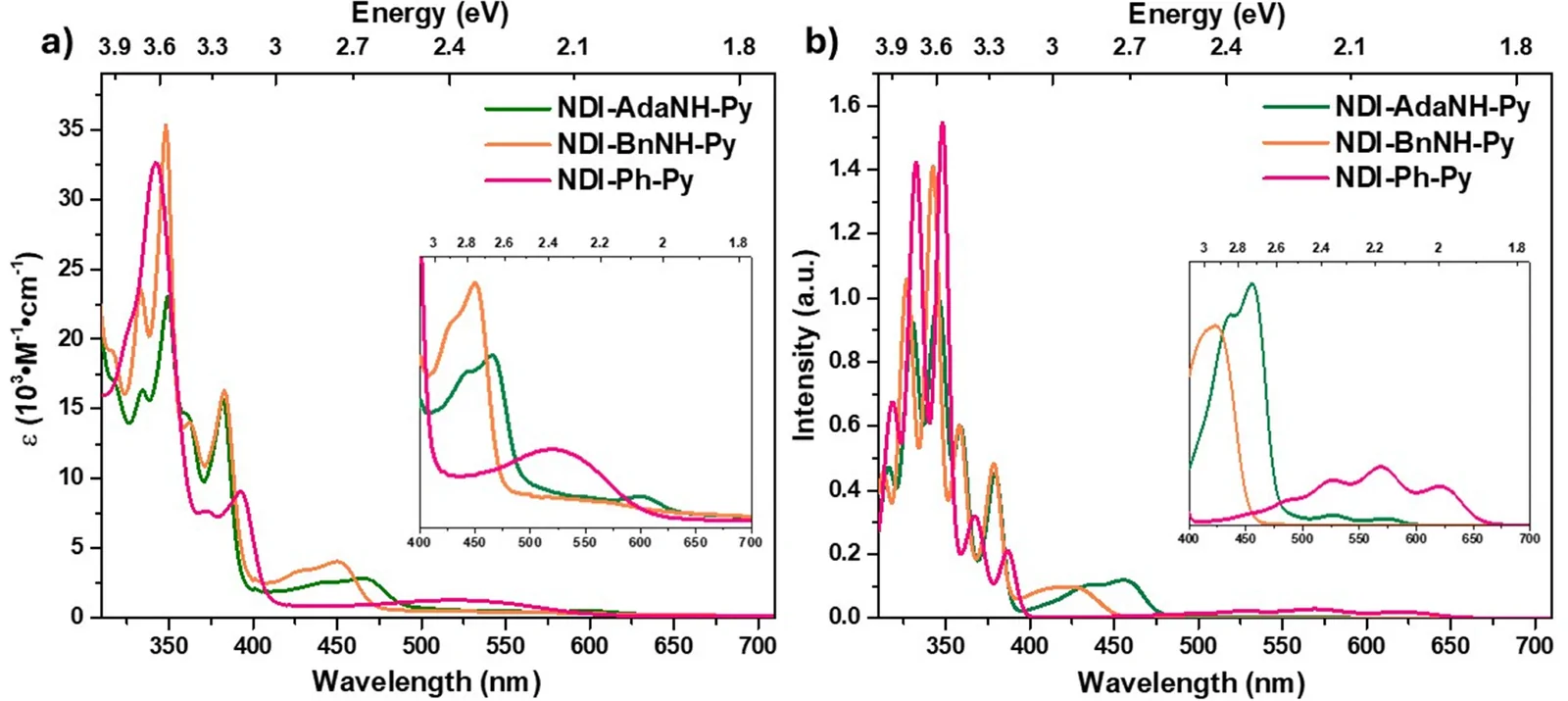
(a) Experimental UV-vis absorption spectra measured in CH_2_Cl_2_ at [*c*] = 1 × 10^−5^ M, and (b) calculated UV-vis absorption spectra of NDI-AdaNH-Py, NDI-BnNH-Py, and NDI-Ph-Py.

**Table 1:** Photophysical data of the different PCPs and their references measured in CH_2_Cl_2_

Compound	*λ*_abs_ [nm] (*ε* [10^3^ M^−1^ cm^−1^])	*E* ^opt^_g_ a [eV]	*λ*_em_ [nm]	*Φ*_Pl_ b [%]
AdaNHBoc-Py	348 (30.5), 453 (3.85)	2.72	477	34
BnNHBoc-Py	347 (35.7), 446 (3.90)	2.76	474	41
PhNHBoc-Py	343 (52.7)	3.33	400	9
NDI	382 (22.6)	3.10	466	<1
NDI-AdaNH-Py	349 (23.1), 382 (15.7), 466 (2.86), 600 (0.51)	—	nd	nd
NDI-BnNH-Py	348 (35.3), 383 (16.3), 450 (4.06), 550 (0.45)	—	nd	nd
NDI-Ph-Py	342 (32.6), 393 (9.10), 520 (1.30)	2.02^c^	nd	nd

a Estimated from the intersection of the normalized absorption and fluorescence emission spectra.

b Relative quantum yield using 9,10-diphenylanthracene as standard (*Φ*_Pl_ = 97% in cyclohexane).

c Calculated from the onset of the lowest-energy absorption band. nd = Not detected.

The ICT band is also observed for NDI-AdaNH-Py and NDI-BnNH-Py, though it appears slightly broadened and red-shifted relative to their reference compounds (Fig. S4). As expected, this transition is not present in NDI-Ph-Py (and the PhNHBoc-Py reference), due to the absence of amine substituents at the 2,7-positions. Our theoretical calculations indicate that several low-lying transitions originate from interactions between the electron-donating Py chromophore and the electron-accepting NDI moiety. The weak broad absorption band in the range of 400–500 nm (3.10–2.48 eV) in NDI-AdaNH-Py and NDI-BnNH-Py also corresponds to the Py ^1^L_b_ transition with partial ICT character, in agreement with previous observations for similar 2,7-amine-substituted pyrenes.^36–41^

In our systems, this band is assigned to the S_4_ state (Fig. 4) and, consistent with experimental data, appears red-shifted for NDI-AdaNH-Py relative to the NDI-BnNH-Py analogue (Fig. 3), following the trend previously reported for their reference molecules.^35^ This can be attributed to the differences in delocalization of the Py HOMO across the linkers (for a detailed discussion, see SI).

**Fig. 4:**
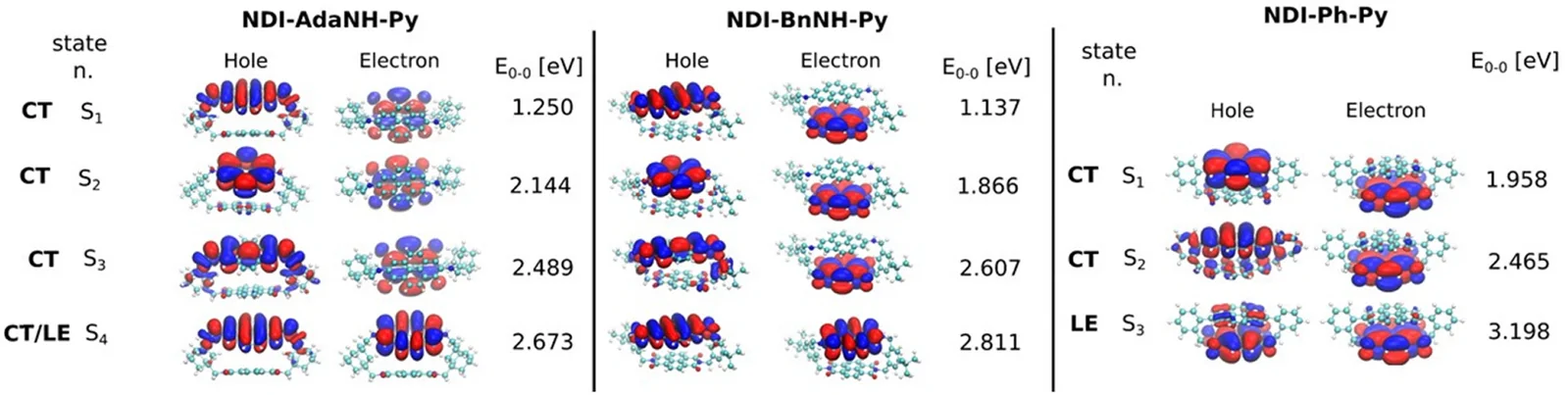
Natural transition orbital analysis for NDI-AdaNH-Py, NDI-BnNH-Py, and NDI-Ph-Py, together with computed corresponding adiabatic energies.

Very interestingly, a new broad and structureless band around 500–600 nm emerges in all D–A PCPs, which is not observed in their reference compounds. This feature cannot be attributed to the individual donor (Py) or acceptor (NDI) chromophores, but rather to a TSCT transition arising from the interaction between the Py and the NDI moiety. Furthermore, the TSCT band intensity correlates directly with rigidity and interchromophore distance. NDI-Ph-Py, which has the shortest linker, exhibits the strongest band [*λ*_max_(*ε*) ∼ 520 nm, (*ε* = 1300 M^−1^ cm^−1^)], while NDI-AdaNH-Py [*λ*_max_(*ε*) ∼ 600 nm, (*ε* = 512 M^−1^ cm^−1^)] and NDI-BnNH-Py [*λ*_max_(*ε*) ∼ 550 nm, (*ε* = 450 M^−1^ cm^−1^)] display a weak and very broad CT band. Notably, the CT band for NDI-AdaNH-Py and NDI-BnNH-Py extends into the near-infrared (NIR), and its intensity increases linearly with concentration, as shown in Fig. S5, ruling out scattering effects.

Our quantum chemical calculations assign this broad band to three distinct low-lying TSCT transitions (Fig. 4). The first excited state (S_1_) is dominated by electron excitation from the HOMO localized on the amine–Py unit to the LUMO localized on the NDI core. It is characterized by a large reorganization energy, resulting in a broad absorption band from 2.00 to 1.15 eV (620–1080 nm) for both AdaNH- and BnNH-linked PCPs. The slightly lower energy (by 12 meV) of the CT state for the NDI-AdaNH-Py molecule can be attributed to differences in the delocalization of the Py HOMO over the linker (Fig. 4), which is consistent with observations in the spectra of the Py reference compounds with AdaNH and BnNH linkers found in our previous work (For more details, see SI).^35^ The second computed CT state (S_2_) exhibits vibronic structure and corresponds to weak bands at 2.08 and 2.35 eV (∼520 and 600 nm), in good agreement with the experimental absorption spectra. The excitation energy for NDI-BnNH-Py is red-shifted by 154 meV relative to NDI-AdaNH-Py, while in the gas phase, the excitation energies are nearly identical (Table S1). This shift can thus be attributed to different state-specific solvation effects. The same trend is also obtained for the other through-space CT states, suggesting that solvent cavity shape, influenced by the molecular geometry and chromophore separation, affects the solvation shifts of the AdaNH- and BnNH-linked PCPs. Like S_1_, the calculated intensity of the S_2_ state is underestimated due to the neglect of thermal fluctuations. Including proper thermal sampling of the structures and resulting transition dipoles can lead to orders of magnitude increase in the intensity (Table S2 in the SI). The third computed CT state (S_3_) appears at energies of 3.30–2.40 eV (380–520 nm), exhibiting low intensity and a clear vibronic feature that overlaps with the S_4_ state.

Across all CT states, the reorganization energies of NDI-BnNH-Py are larger than those of NDI-AdaNH-Py. This can be attributed to the greater structural flexibility of the BnNH-linked system, which enables more substantial geometric relaxation in the excited state. A similar effect has been reported for the NDI homo-PCPs.^35^

A similar analysis was performed for NDI-Ph-Py. Although it exhibits overall spectral similarities to NDI-AdaNH-Py and NDI-BnNH-Py, several key differences arise from the distinct structural and electronic characteristics of the Ph linker. Notably, the Ph linker lacks the electron-donating amine groups present in the AdaNH- and BnNH-linked PCPs, eliminating the HOMO delocalization over the amine-substituted Py.

As a result, the HOMO in NDI-Ph-Py is localized exclusively on the Py (Fig. 4 and Fig. S15). The localization of the orbital leads to its stabilization, resulting in an exchange of the relative energetic ordering of the HOMO and HOMO-1 orbitals. This leads to the shift of the CT state corresponding to the S_1_ state and the intramolecular CT/LE state S_4_ present in NDI-AdaNH-Py and NDI-BnNH-Py to higher excitation energies, accompanied by the loss of transition dipole strength. Instead, the states S_1_ and S_2_ in NDI-Ph-Py both correspond to TSCT transitions from Py to NDI, giving rise to a broad absorption band extending from 2.80 to 1.90 eV (∼440 to 650 nm), in excellent agreement with the experimental spectrum (Fig. 3), with the S_1_ state responsible for the oscillator strength of the band. Although the S_1_ state is similar in character to the S_2_ state in NDI-AdaNH-Py and NDI-BnNH-Py, its transition dipole moment is approximately an order of magnitude larger. This enhancement does not originate from mixing with nearby LE states, but rather from the substantially shorter D–A spatial separation, which increases orbital overlap. Specifically, the shorter spatial separation in NDI-Ph-Py (3.7 Å) results in a significantly larger HOMO–LUMO overlap (0.039), compared to NDI-AdaNH-Py (0.008), where the chromophores are separated by 5.44 Å (Fig. 2). The enhanced orbital overlap in NDI-AdaNH-Py leads to partial delocalization of the HOMO and LUMO orbitals and strengthens the CT transition, giving rise to a transition dipole moment oriented perpendicular to the π-conjugated planes of the NDI and Py moieties (A detailed explanation is provided in the SI). The computed excitation and reorganization energies, transition dipoles, and excited state contributions for all the CT and LE states of the three studied PCPs are summarized in Table S1 and the optical spectra decomposition to the individual transitions is shown in Fig. S12.

The emission spectra of the three D–A PCPs and their corresponding reference compounds were recorded in CH_2_Cl_2_ (Fig. S6 and S7), and the data are summarized in Table 1. The reference compounds AdaNHBoc-Py and BnNHBoc-Py exhibit strong, broad, and featureless emission bands with maxima at 477 and 474 nm, and relative quantum yields of 34% and 41%, respectively. These compounds show clear solvatochromism, with a bathochromic shift observed when the solvent polarity increases from toluene to acetonitrile (Fig. S6). This shift is characteristic of emission from a state with significant CT character, which becomes increasingly stabilized in more polar solvents, resulting in red-shifted fluorescence.^37^ In contrast, the PhNHBoc-Py reference compound displays an emission band centered at *ca.* 400 nm with a relative quantum yield of 9% attributed to a pure π–π* transition. Notably, the fluorescence originating from both donor and acceptor chromophores in all three PCPs is dramatically quenched (Fig. S7) due to photoinduced electron transfer.

### Electrochemical properties

The electrochemical properties of the D–A PCPs and their corresponding reference compounds were investigated using cyclic voltammetry (Fig. 5). The NDI reference exhibits two reversible single-electron reduction processes at −0.63 V and −1.05 V, corresponding to the sequential reduction of the NDI unit to its radical anion and dianion species. All three PCPs display similar reduction profiles, but with the first reduction potential cathodically shifted by approximately 100 mV relative to the NDI reference. This shift suggests that the donor Py moiety influences the electronic properties of the adjacent NDI unit, destabilizing the LUMO level. In the positive potential window, the pyrene-based reference compounds exhibit distinct electrochemical behavior. AdaNHBoc-Py shows two reversible single-electron oxidation processes at 0.50 V and 0.92 V, corresponding to the sequential formation of pyrene radical cation and dication species.^37,38,42,43^ In contrast, BnNHBoc-Py undergoes one irreversible oxidation at 0.59 V and a quasi-reversible oxidation at 1.02 V. The relatively low oxidation potential observed for both compounds indicates their strong tendency to form radical cationic species. Notably, NDI-AdaNH-Py and NDI-BnNH-Py exhibit two reversible oxidation processes that are marginally shifted relative to their corresponding reference compounds (Table 2). These shifts point to a modest electronic interaction between the NDI and Py units, resulting in a small perturbation of the HOMO energy level upon formation of the π-stacked D–A structure. In stark contrast, the reference PhNHBoc-Py undergoes a single irreversible oxidation process at 1.34 V, whereas NDI-Ph-Py exhibits an anodic shift of 70 mV. This observation suggests that the shorter, more rigid linker in NDI-Ph-Py enforces a closer spatial arrangement of the NDI and Py chromophores, thereby increasing electronic coupling and leading to a stabilized HOMO level. It can therefore be deduced that the distinct redox behavior observed for NDI-Ph-Py compared with NDI-AdaNH-Py and NDI-BnNH-Py is primarily governed by the relative spatial arrangement of the NDI and Py units and the substantial involvement of the amine group in the HOMO.

**Fig. 5:**
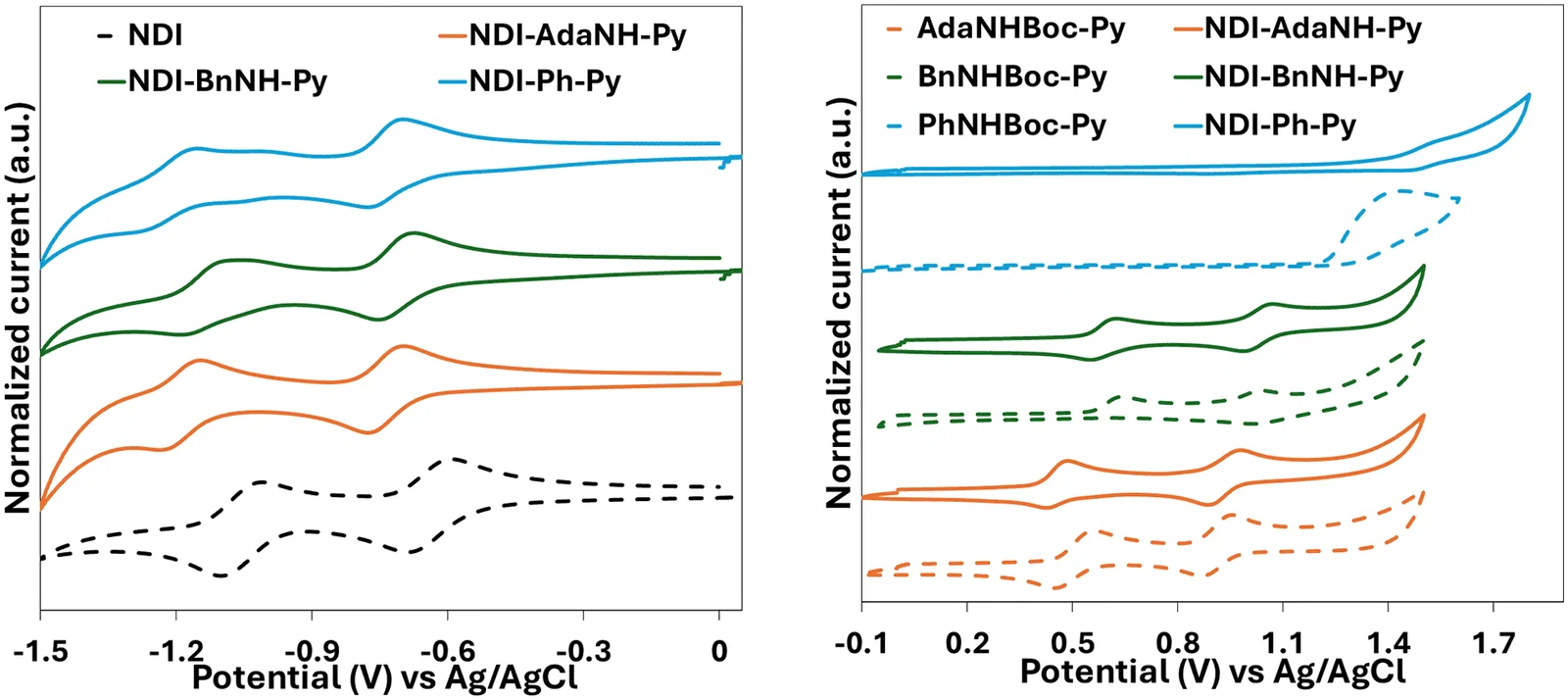
Cyclic voltammograms of the different PCPs with their reference molecules measured in CH_2_Cl_2_ with TBAPF_6_ (0.1 M) as the supporting electrolyte. Pt working electrode, glassy carbon as counter electrode, scan rate 0.1 V min^−1^. (a) negative window, (b) positive window.

**Table 2:** Electrochemical data of the different PCPs and their references. Redox potentials (V) *vs.* Ag/AgCl in CH_2_Cl_2_, HOMO and LUMO energies, electrochemical band gap *E*^el^_*g*_, calculated ionization potential (IP), electron affinity (EA), and bandgap (*E*_g_)

Compound	*E* ^ox1^_1/2_ [V]	*E* ^red1^_1/2_ [V]	HOMO^a^ [eV]	LUMO^a^ [eV]	*E* ^el^_g_ b [eV]	IP^c^ [eV]	EA^c^ [eV]	*E*_g_ c [eV]
AdaNHBoc-Py	0.50	nd	−4.76^d^	nd	nd	−4.82	−1.94	2.88
BnNHBoc-Py	0.58	nd	−4.86^e^	nd	nd	−4.98	−1.92	3.06
PhNHBoc-Py	1.34	nd	−5.56^e^	nd	nd	−5.65	−2.04	3.61
NDI	nd	−0.63	nd	−3.82^d^	nd	−6.66	−3.80	2.86
NDI-AdaNH-Py	0.46	−0.73	−4.71^e^	−3.71^d^	1.00	−4.81	−3.68	1.13
NDI-BnNH-Py	0.59	−0.76	−4.87^e^	−3.76^e^	1.10	−4.97	−3.72	1.25
NDI-Ph-Py	1.41	−0.73	−5.72^e^	−3.77^e^	1.95	−5.76	−3.71	2.05

a The HOMO level was calculated from the onset of the first oxidation potential according to the equation *E*_HOMO_ = [−*e*(*E*_onset*, *ox_ – (Fc^+^/Fc) + 4.8)] eV. The LUMO level was calculated from the onset of the first reduction potential according to the equation *E*_LUMO_ = [*−e*(*E*_onset, red_ – (Fc^+^/Fc) + 4.8)] eV. 4.8 eV is the energy level of ferrocene below the vacuum level.

b Electrochemical bandgap.

c Calculated at DFT level with B3LYP functional and 6-31+G(d,p) basis set and PCM solvation.

d The oxidation potential of Fc^+^/Fc against Ag/AgCl was recorded to be 0.45 V.

e The oxidation potential of Fc^+^/Fc against Ag/AgCl was recorded to be 0.43 V.

These experimental results are fully supported by DFT calculations with B3LYP DFT functional and 6-31+G(d,p) basis set. The solvent effects of the CH_2_Cl_2_ were included within the implicit continuum polarizable model (PCM). The adiabatic electron affinities (EA) and ionization potentials (IP), obtained from the optimized structures as IP = *E*^opt^_+1_ − *E*^opt^_0_ and EA = *E*^opt^_0_ − *E*^opt^_−1_ where *E*^opt^_i_ correspond to the total energy of the optimized structure with the charge *t*, and were in exceptional agreement with the experimental values with errors as low as 0.1 eV (Table 2). The experimentally determined HOMO and LUMO energies, as well as the associated energy gap, are in excellent agreement with the calculated values (more details concerning the calculation of electrochemical properties and corresponding solvent effects can be found in SI; Table S3).

### Transient absorption spectroscopy

To investigate the excited-state dynamics of the three NDI–Py PCPs, femtosecond transient absorption (fsTA) spectroscopy was performed in CHCl_3_ for NDI-AdaNH-Py and NDI-BnNH-Py, and in CH_2_Cl_2_ for NDI-Ph-Py due to its limited solubility in CHCl_3_. Broadband probe pulses covering the 350–720 nm range were used to monitor the photoinduced CT dynamics upon photoexcitation. The TA data were analyzed by a global analysis using a sequential kinetic model (A → B → C → D), yielding species-associated spectra (SAS) and the corresponding time constants. Initial experiments were carried out upon 380 nm excitation, which selectively populates the S_1_ state of the NDI moiety. As illustrated in Fig. 6, NDI-AdaNH-Py exhibits two distinct spectral evolutions following photoexcitation. At early times, two positive excited state absorption (ESA) features centered at ∼470 and 488 nm are observed. These features decay within ∼300 fs, accompanied by the rise of an ESA band with characteristic peaks at 470 and 600 nm. This spectral feature closely resembles the absorption spectrum of the isolated NDI radical anion (NDI˙^−^).^44,45^

**Fig. 6:**
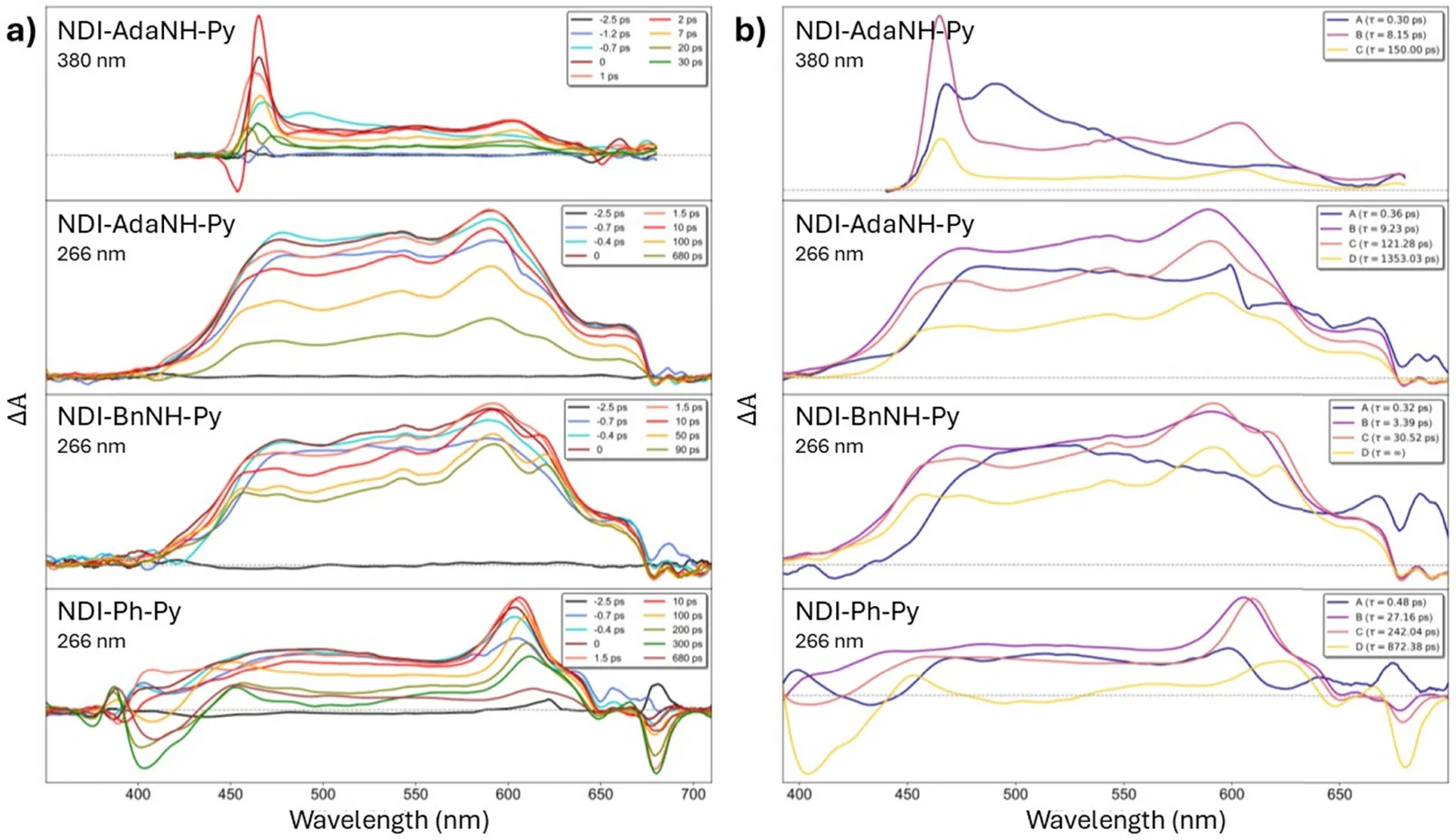
fsTA spectra (a) and SAS (b) of NDI-AdaNH-Py and NDI-BnNH-Py (in CHCl_3_) and NDI-Ph-Py (in CH_2_Cl_2_), excited at 380 and 266 nm.

The appearance of the 470 nm peak within the temporal resolution of the TA experiment suggests that relaxation from the ^1^NDI* state occurs rapidly. Assignment of the long-lived component to the NDI radical anion is consistent with electronic structure calculations of the excited states, which identify the three lowest-energy excited states of NDI-AdaNH-Py as TSCT states with NDI–Py electronic configuration.

Due to the limited solubility of NDI-BnNH-Py and NDI-Ph-Py in CHCl_3_, the absorbance at 380 nm was insufficient to obtain reliable transient signals. To enable a direct comparison among all three PCPs, TA experiments were therefore performed upon 266 nm excitation, where all compounds absorb strongly. However, excitation in the UV introduces substantial excess energy into the system, making detailed interpretation of the resulting dynamics challenging. Therefore, the discussion below focuses primarily on the similarities and differences among the PCPs with different linkers.

The observed transient spectral signatures of NDI-AdaNH-Py and NDI-BnNH-Py are nearly identical (Fig. S18a), indicating the same excited-state evolution upon excitation. The principal difference between the two systems lies in the rates of the excited state evolution. The SAS analysis revealed four spectral components with lifetimes of 360 fs, 9.23 ps, 121.28 ps, and 1353 ps for NDI-AdaNH-Py and 320 fs, 3.39 ps, 30.52 ps, and a long-lived component beyond the time window of our experimental setup for NDI-BnNH-Py. The faster excited state dynamics for NDI-BnNH-Py are consistent with its greater structural flexibility, which permits more extensive geometric relaxation and conformational freedom in the excited state than the more rigid NDI-AdaNH-Py. The early-time TAS spectrum (−0.7 ps component in Fig. 6) exhibits a broad and largely featureless ESA band extending approximately from 450 to 650 nm, markedly different from that observed following 380 nm excitation. This broad spectral feature most likely arises from the overlapping contributions of the ^1^Py* and ^1^NDI* locally excited states, together with a broad band whose signature persists throughout the entire excited-state dynamics. Based on the fact that this band exhibits a similar temporal evolution across the whole spectral region, it can be assigned to a single electronic state, most likely a CT state. This interpretation is consistent with the substantial reduction of the fluorescence quantum yield observed for the PCPs relative to the reference compounds (Fig. S7). The early-time evolution is characterized by decay of the spectra in the region 460 nm to 550 nm and at around 680 nm. This behavior closely resembles the dynamics observed for the reference compounds BnNHBoc-Py and AdaNHBoc-Py (Fig. S18b) and can therefore be attributed to relaxation within the Py moiety, followed by relaxation into the final CT state. The later time dynamics are dominated by a slower decay or rise of the ESA signal around 600 nm, which may indicate partial population of the ^1^NDI* state. Although the initial dynamics represented by the early-time TAS spectra (−0.7 ps component in Fig. 6) are similar for all three systems (Fig. S18a), the subsequent excited-state evolution for NDI-Ph-Py differs significantly from that of NDI-AdaNH-Py and NDI-BnNH-Py. However, since NDI-Ph-Py was measured in CH_2_Cl_2_ whereas NDI-AdaNH-Py and NDI-BnNH-Py were measured in CHCl_3_, a contribution of solvent-dependent effects to these differences cannot be excluded. For NDI-Ph-Py, the initially excited state evolves to a species whose spectral signature can be well represented as a sum of the ^1^NDI* and ^1^Py* signals (Fig. S18c). This spectrum can be assigned to an ^1^NDI* state that is partially delocalized over the ^1^Py* state because of the strong exciton coupling of 668 cm^−1^ as discussed for the absorption spectra. At later times, this spectrum is accompanied by the rise of the stimulated emission (SE) bands at 400–420 nm, 680 nm, and a weaker feature between 470–550 nm. The large energy separation of 1.2 eV between these emission bands suggests the presence of multiple excited state species rather than a single electronic excited state. The 680 nm (1.8 eV) emission appears below the lowest CT state (2 eV), and its relatively sharp SE band suggests formation of a new species rather than emission from a weakly allowed, broad CT state. A plausible assignment for this state is an NDI–Py exciplex, expected to arise from an intermolecular interaction between an electronically excited NDI-Ph-Py molecule and a neighbouring ground-state NDI-Ph-Py molecule, resulting in an excited-state complex stabilized by intermolecular CT interactions.

Additionally, previous reports on conformationally rigid NDI–pyrene-based systems have demonstrated the formation of exciplex species exhibiting emission centred around 670 nm, which has been attributed to the strong electronic coupling within the D–A framework.^46,47^ These precedents provide relevant support for interpreting the corresponding feature observed in our system and therefore, the assignment to an exciplex state.

## Conclusions

In summary, we have developed a new family of D–A PCPs incorporating NDI as the electron acceptor and pyrene as the electron donor, connected through linkers with varying rigidity and flexibility, including adamantane-, benzyl-, and phenylene-based bridges. The nature of the linker plays a crucial role in controlling the spatial arrangement of the chromophores, the extent of electronic coupling, and thus the excited-state dynamics. All three PCPs exhibit clear signatures of TSCT interactions, evidenced by the emergence of broad low-energy absorption bands that are absent in the corresponding reference compounds. The intensity and energy of these CT transitions are highly sensitive to the interchromophore distance and the linker structure. In particular, the shorter and more rigid phenylene bridge in NDI-Ph-Py facilitates stronger orbital overlap and enhanced D–A coupling. This gives rise to a substantially more intense CT absorption band than that observed for the amine-containing AdaNH and BnNH linkers in NDI-AdaNH-Py and NDI-BnNH-Py. The presence of the amine-substituted pyrene donor also leads to distinct redox behavior and multiple TSCT interactions. FsTA spectroscopy further revealed that all PCPs undergo ultrafast photoinduced electron transfer, leading to the formation of multiple CT states. These TSCT dynamics are strongly influenced by linker flexibility and NDI–Py proximity. Specifically, NDI-BnNH-Py exhibits faster excited-state evolution than NDI-AdaNH-Py, consistent with the greater conformational flexibility of the benzyl linker. In contrast, NDI-Ph-Py displays markedly different excited-state dynamics leading to population of states that can be assigned to an NDI–Py exciplex.

Overall, these findings confirm that NDI–Py PCPs are a versatile model system for investigating TSCT processes in structurally well-defined D–A architectures. More broadly, they demonstrate that linker engineering in confined π-stacked systems is an effective strategy for precisely modulating CT interactions and excited-state dynamics. When incorporated into a single-molecule junction, these D–A architectures can enable asymmetric charge transport and molecular rectification, as well as light-controlled conductance modulation. This has potential applications in single-molecule diodes and optical switching. Beyond single-molecule junctions, the regulation of electrical conductivity by TSCT could also be exploited in molecular field-effect transistors and photodetectors. Taken together, these prospects highlight the broader potential of the current TSCT system for advancing molecular electronics and optoelectronics.

## Author contributions

C. N.-V. synthesized and characterised molecules and with the aid of E. Z., H.-M. F. performed transient absorption spectroscopy measurements and analyzed the data. S. D., T. F., U. R. and S.-X. L. conceived and co-supervised the project. V. S. carried out theoretical calculations and analyzed the data. C. N.-V., E. Z. and V. S. wrote the initial draft with input from all authors. S.-X. L. prepared the manuscript with the aid of S. D., U. R. and T. F. All authors have given approval to the final version of the manuscript.

## Conflicts of interest

There are no conflicts to declare.

## Supplementary Material

TC-OLF-D6TC02775J-s001

## Data Availability

The data supporting this article are provided in the supplementary information (SI). Supplementary information: detailed experimental procedures, characterisation data for all newly synthesised compounds, DFT calculations, and additional transient absorption spectra. See DOI: https://doi.org/10.1039/d6tc02775j. Additional references cited in the SI have been listed at the end of this article.^35–37,48–61^
