# Supplementary material for: Programming intramolecular charge transfer in NDI–pyrene paracyclophanes through distance and linker design
Source: J Mater Chem C Mater. 2026 Oct 21. Online ahead of print. doi: 10.1039/d6tc02775j (PMC13718283; doi:10.1039/d6tc02775j)
Supplement: Supplementary file 1 [file TC-OLF-D6TC02775J-s001.pdf]

Supplementary Information (SI) for Journal of Materials Chemistry C.
This journal is © The Royal Society of Chemistry

Supplementary information

Programming Intramolecular Charge Transfer in NDI–Pyrene Paracyclophanes through Distance and Linker Design

Camila Negrete-Vergara[a], Vladislav Slama[b], Elnaz Zyaee[c], Hans-Martin Frey[c], Silvio Decurtins[a], Thomas Feurer[c], Ursula Rothlisberger[b*], and Shi-Xia Liu[a*]

[a]Department of Chemistry, Biochemistry and Pharmaceutical Sciences, W. Inäbnit Laboratory for Molecular Quantum Materials and WSS-Research Center for Molecular Quantum Systems, University of Bern, Freiestrasse 3, 3012 Bern, Switzerland.

[b]Laboratory of Computational Chemistry and Biochemistry, École Polytechnique Fédérale de Lausanne.

[c]Institute of Applied Physics, University of Bern, Sidlerstrasse 5, 3012 Bern, Switzerland.

E-mail: ursula.roethlisberger@epfl.ch; shi-xia.liu@unibe.ch

Table of Contents

Experimental Section 2
Synthesis of NDI-AdaNH-Py 2
Synthesis of NDI-BnNH-Py 5
Synthesis of NDI-Ph-Py 7
^{1}H-NMR upfield shift effect 9
UV-vis absorption spectra 10
Fluorescence spectra 11
Theoretical Section 12
Structure of the paracyclophanes 12
Optical spectra calculation 12
Spectral densities 13
Paracyclophanes' excited state and optical properties 15
Transition density of the CT state for NDI-Ph-Py 21
Electrochemical properties 23
Transient absorption spectroscopy 24
Setup 24
Reference Compounds 25

References 28
NMR spectra..30
Mass spectra 42

Experimental Section

Air- and/or water-sensitive reactions were conducted under nitrogen and dry, freshly distilled solvents were used. Chemicals used to synthesize the compounds were purchased from commercial suppliers (Sigma-Aldrich, TCI, Alfa Aesar, or Thermo Fischer) and used without further purification. UV-Vis absorption spectra were measured on a Jasco V-730 spectrophotometer using quartz cuvettes with an optical path of 1 cm. Fluorescence spectra were collected on a Jasco FP-8300 spectrophotometer. Relative quantum yield was measured using 1,9-diphenylanthracene as a standard ($φ_{pl}$ = 0.97 in cyclohexane, $λ_{exc}$ = 355 nm). Automated flash chromatography (Silica gel) was performed with a Biotage Isolera Four instrument using a gradient of solvents that were given by the Rf values from the TLC. ^{1}H and ^{13}C NMR spectra were recorded on a Bruker Avance 300 or 400 spectrometer (^{1}H NMR = 300 MHz or 400 MHz; ^{13}C NMR = 75 MHz or 101 MHz) Chemical shifts are reported in parts per million (ppm) and are referenced to the residual solvent peak ($CDCl_3$, δ ^{1}H = 7.26 ppm, CD_2Cl_2, δ ^{1}H = 5.32 ppm, and DMSO-d_6, δ ^{1}H = 2.50 ppm; $CDCl_3$, δ ^{13}C =77.16 ppm, CD_2Cl_2, δ ^{13}C = 53.84 ppm, and DMSO-d_6, δ ^{13}C = 39.52 ppm). The following abbreviations were used: s (singlet), d (doublet), and m (multiplet). High-resolution mass spectra (HR-MS) were measured by the Analytical Research and Services (ARS) of the University of Bern, Switzerland, on a Thermo Fisher LTQ Orbitrap XL using Nano Electrospray Ionization. Cyclic voltammetry (CV) was performed in a three-electrode cell with a Pt working electrode, a glassy carbon counter-electrode, and an Ag/AgCl reference electrode. The electrochemical experiments were conducted under an oxygen-free atmosphere in dichloromethane with $TBAPF_6$ (0.1 M) as the supporting electrolyte.

Synthesis of NDI-AdaNH-Py

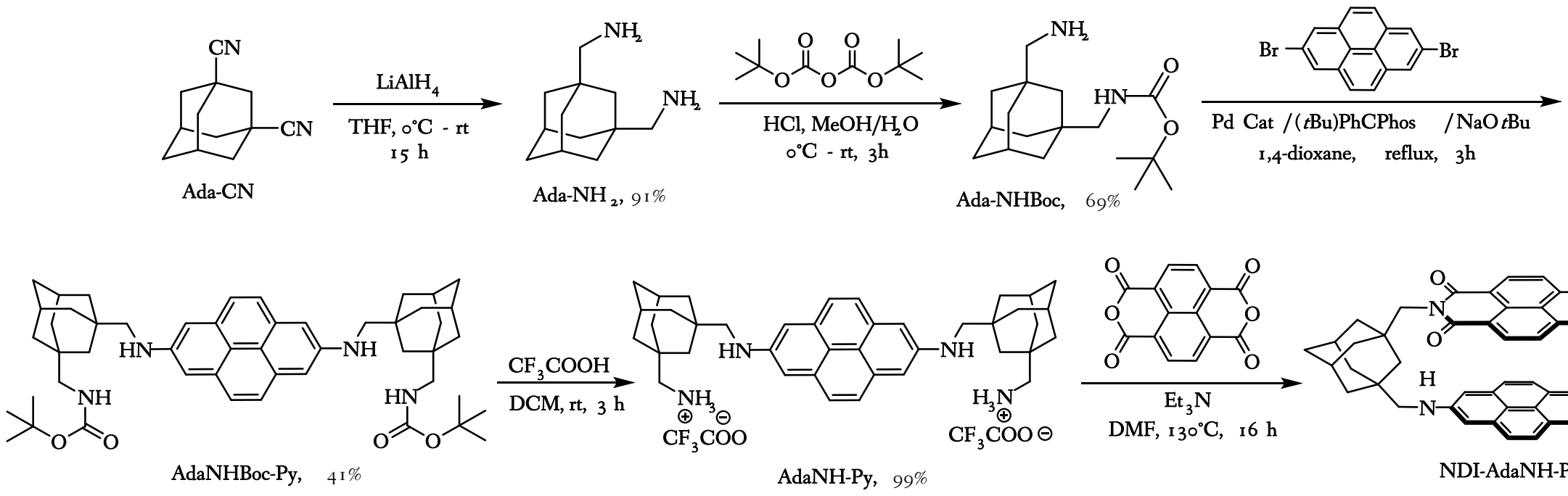

Ada-NH$_2$

In a well-dried two-neck round-bottom flask, a suspension of $LiAlH_4$ in THF (1.0 M, 25 mL) was added under a nitrogen atmosphere. The mixture was cooled to 0°C with an ice bath, followed by the slow addition of Ada-CN (466 mg, 2.5 mmol) dissolved in dry THF (20 mL). After 30 minutes, the reaction was slowly warmed up to room temperature and left to react for a further 15 hours under constant stirring. The white suspension formed was exposed to air and then cooled down to 0°C. After that, it was carefully quenched with the following in this order: water (1 mL, added drop by drop), then NaOH (0.6 mL, 15% wt/v), and finally water (3 mL; vapors and gas are released during this process). The mixture was stirred for another 30 minutes, and the inorganic precipitate was filtered off using a glass frit (pore size 4) and washed with THF. The organic phase was dried over anhydrous Na_2SO_4 and then evaporated under reduced pressure to obtain the product, which appeared as a colorless oil. No further purification was necessary (440 mg, 91%). Store the product under inert gas at a temperature below 4°C due to its instability under ambient conditions. ^{1}H NMR (300 MHz, $CDCl_3$, ppm): δ=2.36 (s, 4H), 2.15 – 2.02 (m, 2H), 1.61 (m, 2H), 1.50 – 1.31 (m, 8H), 1.16 (m, 6H). ^{13}C NMR (75 MHz, $CDCl_3$, ppm): δ=54.84, 42.57, 39.84, 36.96, 34.67, 28.70. HR-MS (ESI, positive): *m/z* calcd for $C_{12}H_{22}N_2$+H^+: 195.1856 [*M*+H]$^+$; found: 195.1855.

Ada-NHBoc

In a two-neck round-bottom flask and under a nitrogen atmosphere, 1 equivalent of Ada-NH$_2$ (380 mg, 1.96 mmol) was dissolved in MeOH (15 mL). The solution was cooled to 0°C, followed by the slow addition of HCl solution in diethyl ether (1.0 M, 1.96 mL). After that, the mixture was warmed to room temperature and stirred for 30 minutes. Subsequently, water (4 mL) was added and stirred for a further 30 minutes. Following this, a solution of 1.5 equivalents of (Boc)$_2$O (640 mg, 2.94 mmol) in MeOH (20 mL) was added slowly over

the course of 1 h, and the resulting solution was stirred for an additional hour. The mixture was concentrated under vacuum, then the residue was diluted with 10 mL of water. Next, the aqueous solution was extracted with two portions of Et_2O (15 mL each) to remove unreacted amine and/or double-protected amine. After treating the aqueous phase with a solution of NaOH (2 equivalents in 10 mL of water), the monoprotected amine was extracted with CH_2Cl_2 (3 x 30 mL). The organic phase was washed with brine, dried over anhydrous Na_2SO_4, and then evaporated under reduced pressure to obtain a colorless oil, requiring no further purification (398 mg, 69%). ^{1}H NMR (300 MHz, $CDCl_3$, ppm): δ=4.57 (s, 1H), 2.84 (d, *J* = 6.5 Hz, 2H), 2.38 – 2.36 (m, 2H), 2.08 – 2.06 (m, 2H), 1.68 – 1.59 (m, 5H), 1.46 – 1.35 (m, 16H), 1.20 – 1.17 (m, 2H);^{13}C NMR (75 MHz, $CDCl_3$, ppm): δ=156.48, 54.83, 54.69, 52.25, 42.56, 39.86, 39.83, 39.59, 36.95, 36.68, 34.67, 34.63, 28.69, 28.57, 28.52; HR-MS (ESI, positive): *m/z* calcd. for $C_{17}H_{30}N_2O_2+H^+$: 295.2380 $[M+H]^+$; found: 295.2370.

AdaNHBoc-Py

A mixture of 2,7-dibromopyrene (240 mg, 0.68 mmol), sodium *tert*-butoxide (Na*t*BuO, 156 mg, 1.63 mmol), 2-aminobiphenylpalladium methanesulfonate (10.5 mg), (*t*Bu)PhCPhos (5.5 mg), and Ada-NHBoc (440 mg, 1.50 mmol) dissolved in dry 1,4-dioxane (10 mL) was stirred at 100 °C under a positive flow of nitrogen gas. The mixture was monitored by TLC (EtOAc/n-heptane 3:7) and the reaction was stopped when the pyrene precursor was completely consumed. The reaction was cooled to room temperature and diluted with EtOAc. The solution was filtered through Celite and concentrated under reduced pressure. The crude was purified by automated flash column chromatography (R_f= 0.32, EtOAc/n-heptane 3:7). Yellow powder (220 mg, 41%). ^{1}H NMR (300 MHz, $CDCl_3$, ppm): δ = 7.77 (s, 4H), 7.33 (s, 4H), 4.59 (s, 2H), 3.08 (s, 4H), 2.89 (d, *J* = 6.5 Hz, 4H), 2.13 (s, 4H), 1.69 – 1.60 (s, 14H), 1.49 – 1.40 (m, 30H). ^{13}C NMR (75 MHz, $CDCl_3$, ppm): δ=156.49, 146.52, 131.19, 127.09, 119.02, 109.85, 79.20, 56.66, 52.21, 43.37, 40.41, 39.78, 36.56, 34.83, 34.63, 28.58, 28.54. HR-MS (ESI, positive): *m/z* calcd. for $C_{50}H_{66}N_4O_4+H^+$: 787.5157 $[M+H]^+$; found: 787.5131.

AdaNH-Py

AdaNHBoc-Py (220 mg, 0.28 mmol) was dissolved in CH_2Cl_2 (30 mL) under a nitrogen atmosphere and protected from light. Subsequently, an excess of trifluoroacetic acid (5.1 mL, 67 mmol) was added, and the mixture was stirred for 3 hours. The solution was then evaporated to yield an oily residue. Additional CH_2Cl_2 was added and evaporated to remove unreacted acid, forming a green solid. The product was then fully

crushed by adding n-pentane and then evaporated iunder reduced pressure to obtain a green solid in quantitative yield.^{1}H NMR (400 MHz, DMSO, ppm): δ=7.76 – 7.42 (m, 10H), 7.42 (s, 4H), 3.01 (s, 4H), 2.60 – 2.55 (m, 4H), 2.11 – 2.09 (m, 4H), 1.72 – 1.37 (m, 24H). ^{13}C NMR (101 MHz, DMSO, ppm): δ=146.62, 130.23, 126.66, 117.69, 117.40, 114.49, 109.32, 55.66, 49.83, 42.06, 38.32, 35.55, 34.47, 32.35, 27.64. HR-MS (ESI, positive): *m/z* calcd. for $C_{40}H_{52}N_4^{2+}$: 294.2091[*M*]$^{2+}$; found: 294.2090.

NDI-AdaNH-Py

A solution of naphthalenetetracarboxylic dianhydride (NDTA, 32 mg, 0.123 mmol) in anhydrous DMF (50 mL) was slowly added (over one hour) to a solution of AdaNH-Py (120 mg, 0.147 mmol) in a mixture of Et_3N (1 mL) in warm anhydrous DMF at 90 °C (70 mL) under a nitrogen atmosphere. The mixture was then heated at 130 °C with constant stirring for 15 h. After that, the solvent was evaporated under reduced pressure. The remaining solid was dissolved in $CHCl_3$ (50 mL) and filtered with a frit (pore size 4). The resulting brown solution was evaporated under a high vacuum to eliminate any residual DMF. The dark residue was initially purified by silica gel column chromatography (eluent: DCM/isopropanol = 100:3, v/v). This was followed by gel permeation chromatography (THF, Bio-Beads S-X1 Support). Finally, the purified compound was heated at 180°C under a high vacuum ($1x10^{-6}$ bar) to give the title compound as a dark solid (17 mg, 17%). ^{1}H NMR (400 MHz, $CDCl_3$, ppm): δ=8.48 (s, 4H), 7.45 (s, 4H), 7.07 (s, 4H), 3.90 (s, 4H), 3.17 (s, 4H), 2.10 (s, 4H), 1.67 – 1.58 (m, 17H), 1.49 – 1.43 (m, 9H). ^{13}C NMR (101 MHz, $CDCl_3$, ppm): δ=163.85, 146.06, 130.85, 130.38, 126.67, 126.17, 125.87, 118.10, 109.25, 54.67, 51.64, 43.35, 41.98, 41.50, 37.02, 36.45, 36.22, 29.22. HR-MS (ESI, positive): *m/z* calcd. for = $C_{54}H_{50}N_4O_4$+H^{+}: 819.3905 [*M*+H]$^{+}$; found: 819.3927.

Synthesis of NDI-BnNH-Py

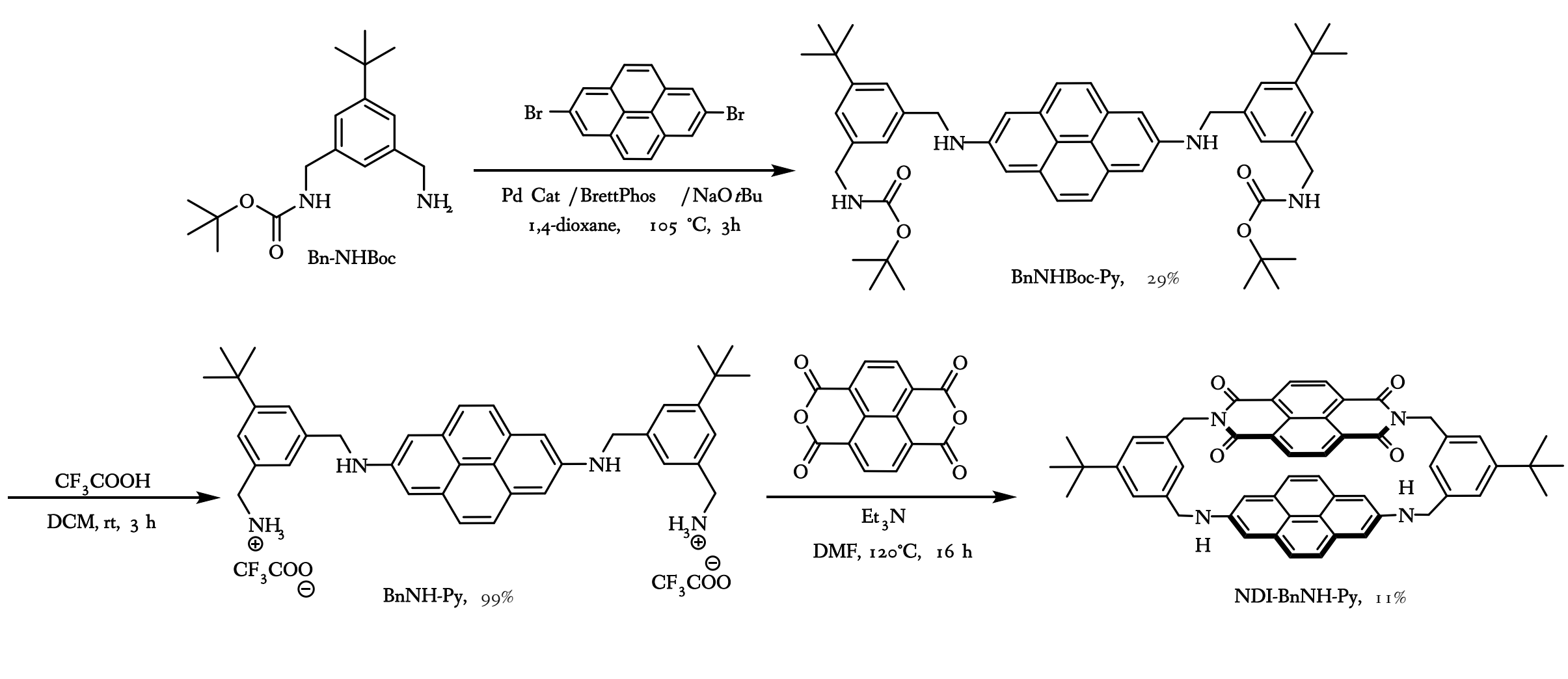

BnNHBoc-Py

$Pd_2(dba)_3$ (23.4 mg, 0.0256 mmol), brettPhos (27.5 mg, 0.0512 mmol), and sodium *tert*-butoxide (NaO*t*Bu, 148 mg, 1.54 mmol) were suspended in 1,4-dioxane (5 mL) under a nitrogen atmosphere. Then, the suspension was heated at 65 °C for 90 minutes. After that, 2,7-dibromopyrene (230 mg, 0.64 mmol) was added, and subsequently, Bn-NHBoc (450 mg, 1.54 mmol) dissolved in 1,4-dioxane (5 mL). The reaction was stirred at 105 °C, monitored by TLC (EtOAc/n-heptane 3:7), and stopped when the pyrene precursor was completely consumed. The reaction was cooled to room temperature and diluted with EtOAc (30 mL). The solution was filtered through a pad of Celite and concentrated under reduced pressure. The crude was purified by automated flash column chromatography (R_f=0.34, EtOAc/n-heptane 3:7). Yellow powder (145 mg, 29%).^{1}H NMR (400 MHz, $CDCl_3$, ppm): δ=7.79 (s, 4H), 7.41 – 7.38 (m, 6H), 7.25 –7.21 (m, 4H), 4.82 (s, 2H), 4.53 (s, 4H), 4.33 – 4.31 (m, 4H), 1.44 (s, 18H), 1.32 (s, 18H). ^{13}C NMR (101 MHz, $CDCl_3$, ppm): δ=156.07, 152.33, 145.77, 139.73, 139.27, 131.31, 127.20, 124.17, 123.75, 119.31, 110.06, 49.44, 45.08, 34.92, 31.54, 28.56. HR-MS (ESI, positive): *m/z* calcd. for $C_{50}H_{62}N_4O_4$+H^+: 783.4844 [*M*+H]$^+$; found: 783.4852.

BnNH-Py

BnNHBoc-Py (165 mg, 0.201 mmol) was dissolved in CH_2Cl_2 (30 mL) under a nitrogen atmosphere and protected from light. Subsequently, an excess of trifluoroacetic acid (4 mL, 53 mmol) was added, and the mixture was stirred for 3 hours. The solution was then evaporated to yield an oily residue. Additional CH_2Cl_2 was added and evaporated to remove unreacted acid, forming a solid. The product was then fully crushed by adding n-pentane and then evaporated under reduced pressure to obtain a green solid in quantitative yield. ^{1}H NMR (300 MHz, DMSO, ppm) δ 8.17 (s, 6H), 7.75 (s, 4H), 7.57 (s, 2H), 7.41 – 7.36 (m, 8H), 4.50 (s, 4H), 4.04 – 3.98 (q, *J* = 5.9 Hz, 4H), 1.27 (s, 18H). ^{13}C NMR (75 MHz, DMSO, ppm): δ=130.29, 126.77, 125.14, 109.83, 42.57, 34.53, 31.08. HR-MS (ESI, positive): *m/z* calcd. for $C_{40}H_{48}N_4^{2+}$ = 292.1934 [*M*]$^{2+}$; found: 292.1931.

NDI-BnNH-Py

A solution of naphthalenetetracarboxylic dianhydride (NDTA, 45 mg, 0.167 mmol) in anhydrous DMF (70 mL) was slowly added (over one hour) to a solution of *t*BuPh-NH-Py (160 mg, 0.200 mmol) with Et_3N (1 mL) in warm anhydrous DMF at 90 °C (80 mL) under a nitrogen atmosphere. The mixture was then heated at 130 °C with constant stirring for 15 h. After that, the solvent was evaporated under reduced pressure. The

brown residue was purified by silica gel column chromatography (eluent: DCM/EtOAc = 95:5, v/v), and the solid obtained was washed with diethyl ether to give the title compound as a brown solid (15 mg, 11%). ^{1}H NMR (400 MHz, CD_2Cl_2, ppm): δ=8.08 (s, 4H), 7.43 - 7.40 (m, 4H), 7.30 - 7.28 (m, 6H), 7.03 (s, 4H), 5.16 (s, 4H), 4.59 (s, 4H), 1.41 (s, 18H). (101 MHz, CD_2Cl_2, ppm): δ=31.66. (Solubility was not sufficient to record a full spectrum.) HR-MS (ESI, positive): *m/z* calcd. for = $C_{54}H_{46}N_4O_4$+H^+: 815.3592 [*M*+H]$^+$; found: 815.3586.

Synthesis of NDI-Ph-Py

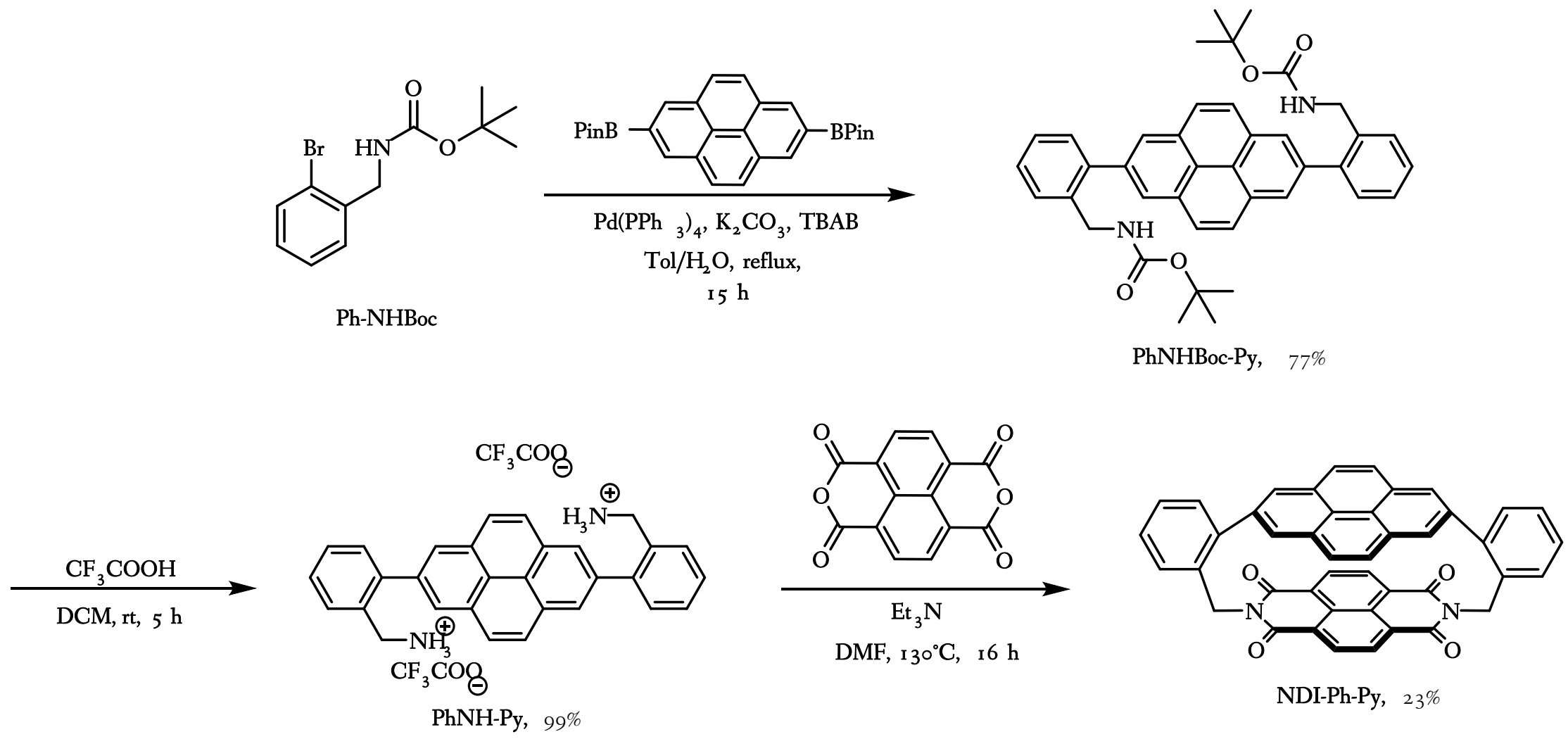

PhNHBoc-Py

2,7-diBpin-pyrene (454 mg, 1.00 mmol), $Pd(PPh_3)_4$ (46.2 mg, 0.04 mmol), K_2CO_3 (276 mg, 2.00 mmol), Tetrabutylammonium bromide (TBAB, 32.2 mg, 0.10 mmol), and Ph-NHBoc (798 mg, 2.80 mmol) were added to a two-neck round-bottom flask (50 mL) under a nitrogen atmosphere. Then, toluene (10 mL) and water (1 mL) were added to the mixture, which was left to react at 105 °C for 24 hours. After that, the

suspension was cooled, and DCM (20 mL) was added. The solution was washed first with water (50 mL) and then with brine (50 mL). The organic phase was dried over Na_2SO_4, and the solvent was evaporated under reduced pressure to produce an oil, which was then crushed with n-pentane. Finally, the crude was purified by precipitation using a mixture of 1:4 DCM/Hexane, to yield a white solid. (471 mg, 77%). ^{1}H NMR (300 MHz, $CDCl_3$, ppm): δ=8.15 (s, 4H), 8.12 (s, 4H), 7.60 – 7.36 (m, 8H), 4.67 (s, 2H), 4.40 (d, *J* = 5.9 Hz, 4H), 1.38 (s, 18H).^{13}C NMR (75 MHz, $CDCl_3$, ppm): δ=155.87, 141.78, 138.72, 136.68, 131.20, 131.00, 128.31, 128.10, 128.03, 127.40, 125.89, 123.69, 42.80, 28.48. HR-MS (ESI, positive): *m/z* calcd. for = $C_{40}H_{40}N_2O_4$+Na^+: 635.2881 $[M+Na]^+$; found: 635.2880.

PhNH-Py

PhNHBoc-Py (200 mg, 0.28 mmol) was dissolved in CH_2Cl_2 (30 mL) under a nitrogen atmosphere and protected from light. Subsequently, an excess of trifluoroacetic acid (5.1 mL, 67 mmol) was added, and the mixture was stirred for 5 hours. The solution was then evaporated to yield an oily residue. Additional CH_2Cl_2 was added and evaporated to remove unreacted acid, forming a light brown solid. The product was then fully crushed by adding n-pentane and then evaporated under reduced pressure to obtain a brown solid in quantitative yield.^{1}H NMR (400 MHz, DMSO, ppm): δ=8.37 (s, 4H),), 8.31 – 8.26 (m, 10H), 7.76 – 7.74 (m, 2H), 7.64 – 7.58 (m, 6H), 4.12 (s, 4H). ^{13}C NMR (101 MHz, DMSO, ppm): δ=141.54, 137.65, 131.61, 131.02, 130.67, 128.46, 128.41, 128.15, 127.93, 125.95, 122.68. HR-MS (ESI, positive): *m/z* calcd. for $C_{30}H_{26}N_2^{2+}$: 207.1043 $[M]^{2+}$; found: 207.1041.

NDI-Ph-Py

A solution of naphthalenetetracarboxylic dianhydride (NDTA, 70 mg, 0.260 mmol) in anhydrous DMF (70 mL) was slowly added (over one hour) to a solution of PhNH-Py (200 mg, 0.312 mmol) with Et_3N (1 mL) in warm anhydrous DMF at 90 °C (80 mL) under a nitrogen atmosphere. The mixture was then heated at 130 °C with constant stirring for 15 h. After that, the solvent was evaporated under reduced pressure. The pink residue was purified by silica gel column chromatography (eluent: DCM/EtOAc = 95:5, v/v) to give the title compound as a pink solid (38 mg, 23%). ^{1}H NMR (300 MHz, $CDCl_3$, ppm): δ=8.11 (s, 4H), 7.75 (s, 4H), 7.61 (s, 4H), 7.52 – 7.51 (m, 4H), 7.39 – 7.38 (m, 4H), 5.67 (s, 4H). Solubility was not sufficient to record a ^{13}C NMR. HR-MS (ESI, positive): *m/z* calcd. for = $C_{44}H_{24}N_2O_4$+Na^+: 667.1635 $[M+Na]^+$; found: 667.1629.

^{1}H-NMR upfield shift effect

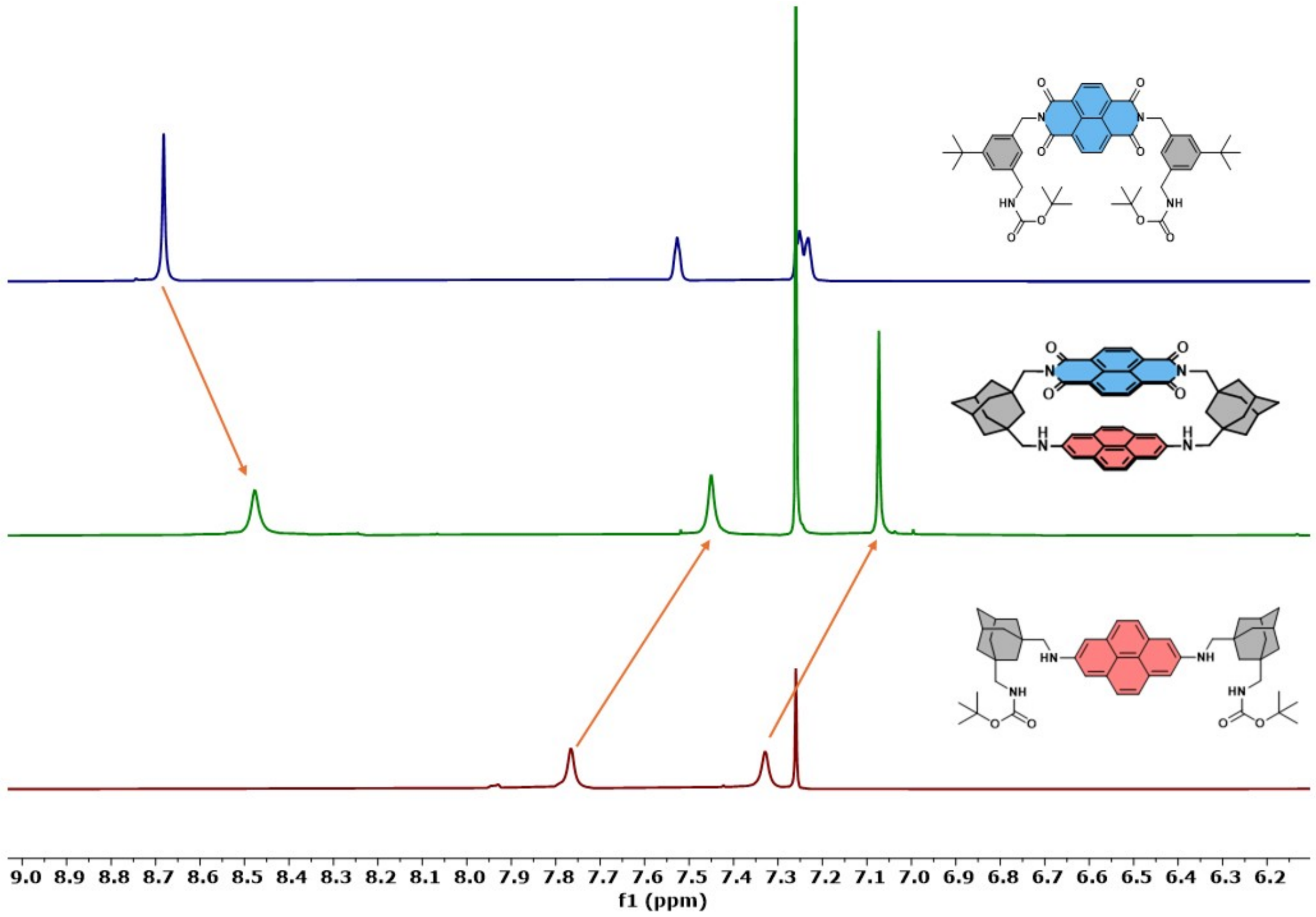

Figure S1. Partial ^{1}H NMR spectra of **NDI**, **NDI-AdaNH-Py**, and **AdaNHBoc-Py** in $CDCl_3$.

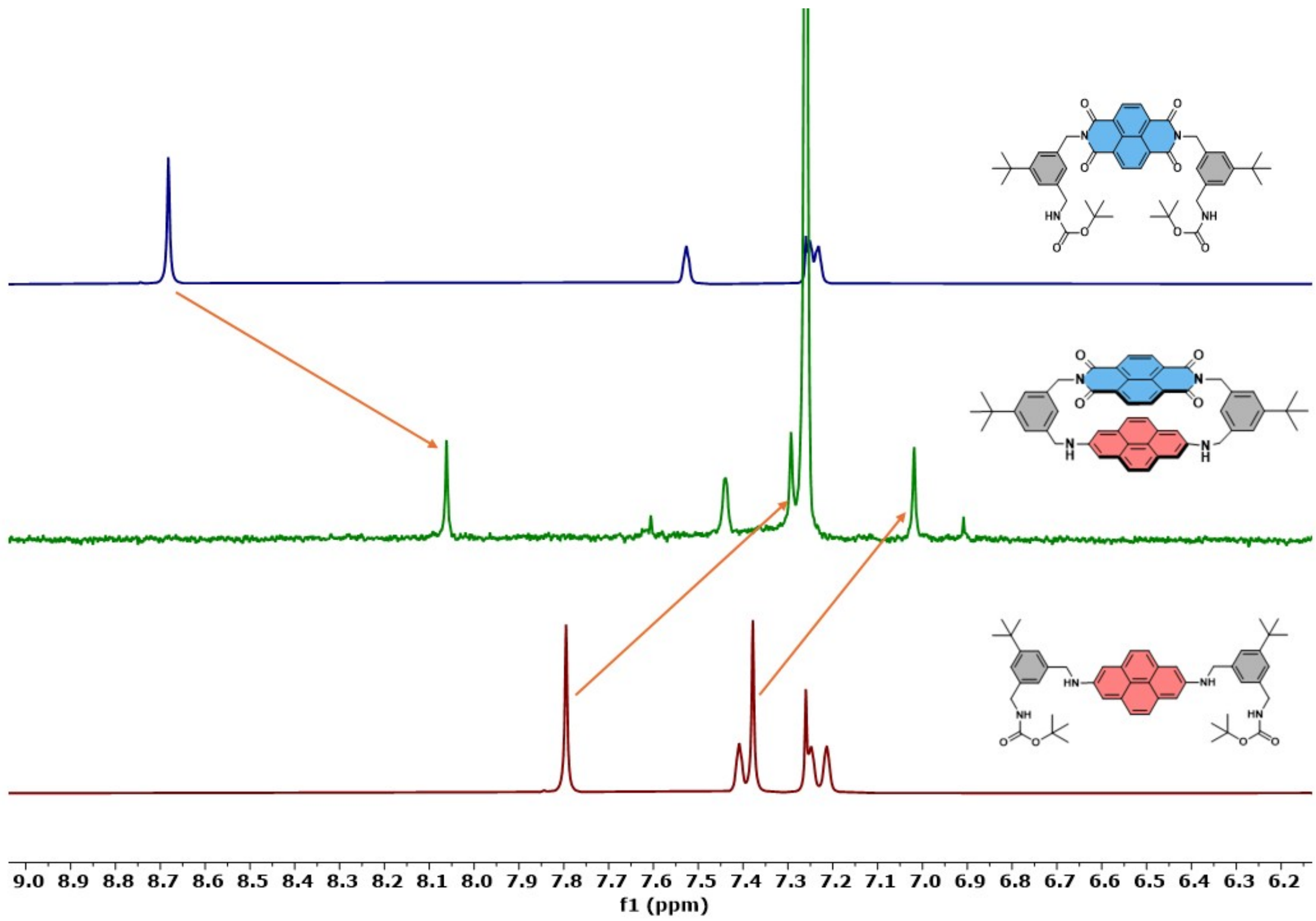

Figure S2. Partial ^{1}H NMR spectra of **NDI**, **NDI-BnNH-Py**, and **BnNH-Py** in $CDCl_3$.

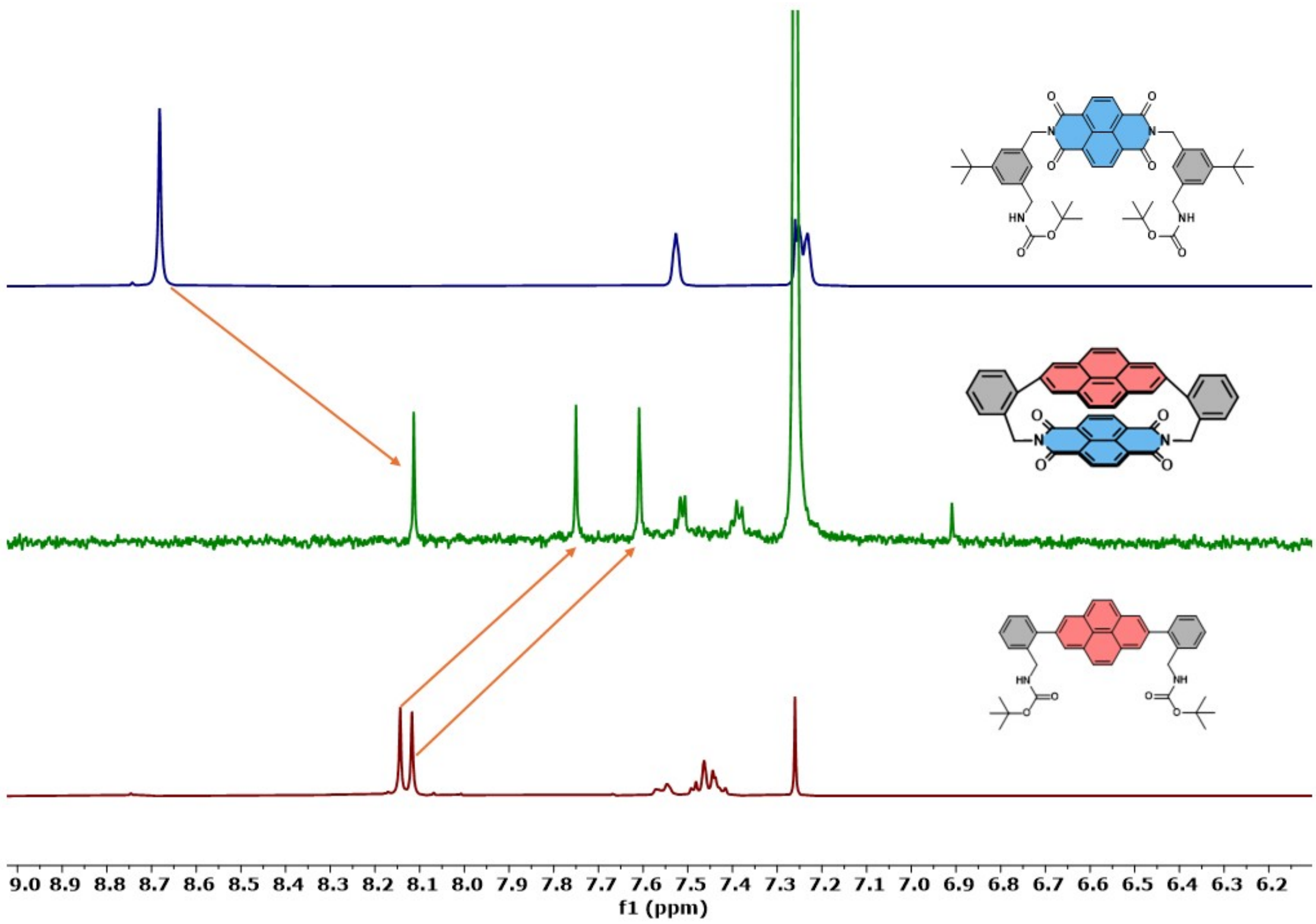

Figure S3. Partial ^{1}H NMR spectra of **NDI**, **NDI-Ph-Py**, and **PhNHBoc-Py** in $CDCl_3$.

UV-vis absorption spectra

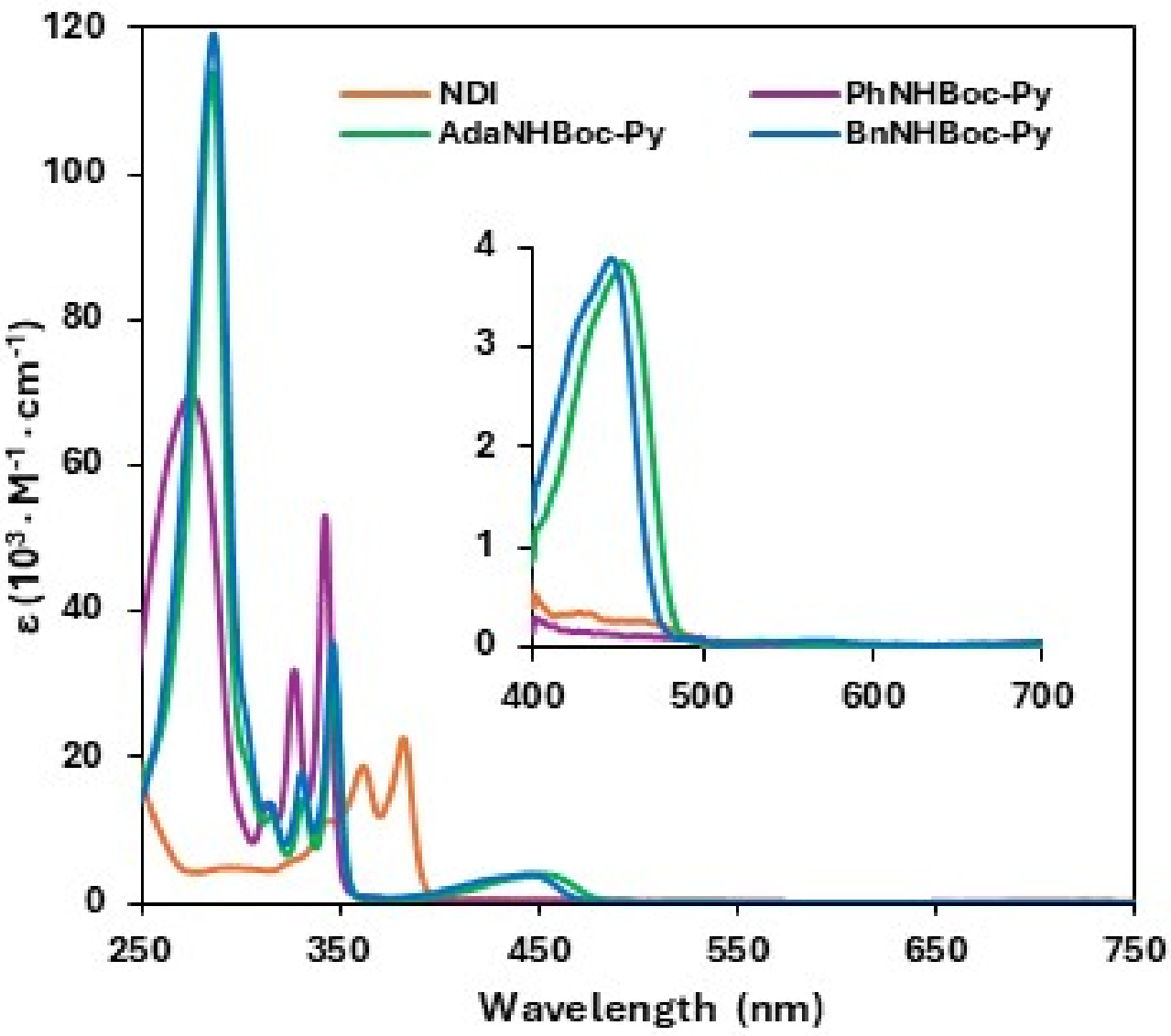

Figure S4. Experimental UV-vis absorption spectra of the reference compounds measured in CH_2Cl_2 for NDI and **Ph-NHBoc-Py** (c = 1x10^{-5} M), and **AdaNHBoc-Py** and **BnNHBoc-Py** (c = 5x10^{-6} M).

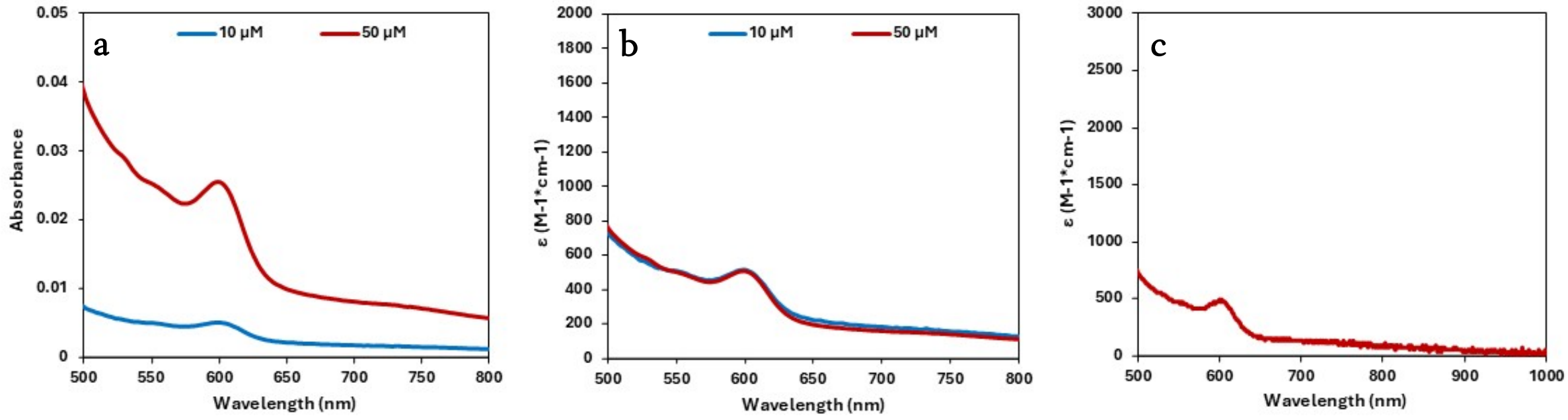

Figure S5. UV-vis absorption spectra of **NDI-AdaNH-Py** in CH_2Cl_2. (**a**) at two different concentrations: blue ($1x10^{-5}$ M) and red ($5x10^{-5}$ M), (**b**) normalized UV-vis absorption spectra at different concentrations. (**c**) UV-Vis-NIR absorption spectrum of **NDI-AdaNH-Py** in CH_2Cl_2 ($5x10^{-5}$ M).

Fluorescence spectra

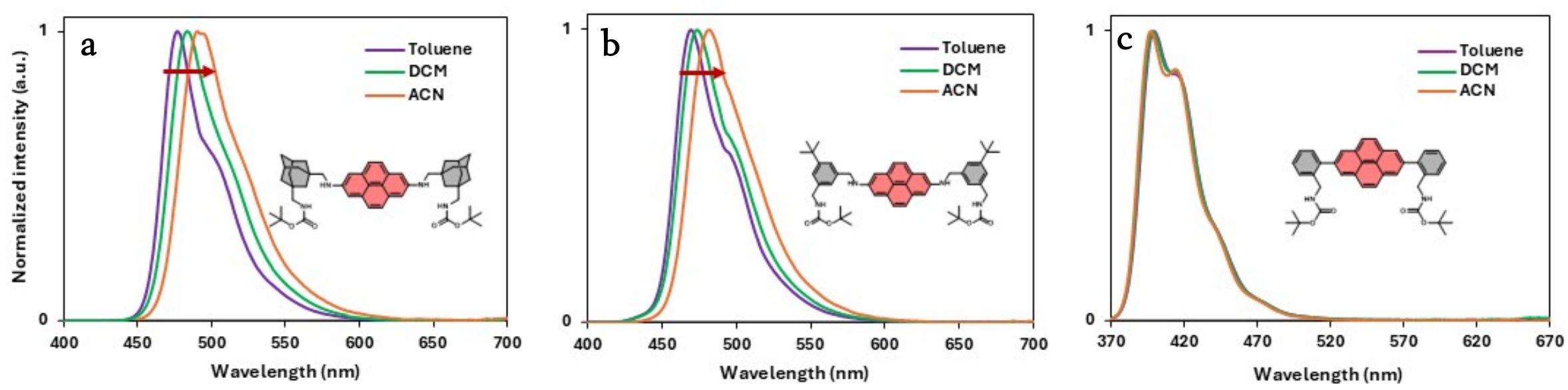

Figure S6. Fluorescence spectra of (**a**) **AdaNHBoc-Py** ($\square_{exc}$ = 290 nm, c = $5x10^{-6}$ M), (**b**) **BnNHBoc-Py** ($\square_{exc}$ = 290 nm, c = $5x10^{-6}$ M), and (**c**) **PhNHBoc-Py** ($\square_{exc}$ = 343 nm, c = $1x10^{-5}$ M) measured in different solvents.

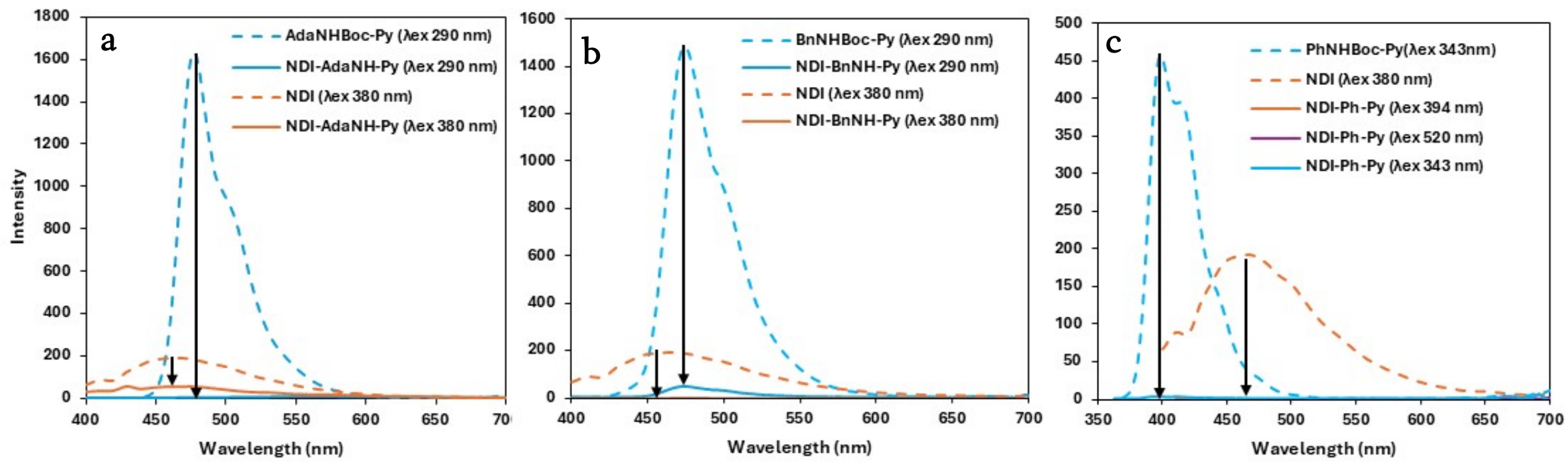

Figure S7. Fluorescence spectra and quenching effect of the different PCPs vs. reference molecules in CH_2Cl_2. (**a**) **NDI-AdaNH-Py** (c = 5x10^{-6} M), (**b**) **NDI-BnNH-Py** (c = 5x10^{-6} M), and (**c**) **NDI-Ph-Py** (c = 1x10^{-5} M).

Theoretical Section

Structure of the paracyclophanes

The molecular geometries were optimized at DFT B3LYP level with 6-31+G(d,p) basis set and solvent effects included by the polarizable continuum model (PCM). The ground state DFT calculations and excited state TDDFT calculations were performed with Gaussian 16[1].

Optical spectra calculation

The excited state properties were computed employing a two-step computational procedure[2]. In the first step, linear-response time-dependent density functional theory (TD-DFT) using the range-separated ωB97XD functional with 6-31+G(d,p) basis set was applied. This method provides a reliable description of electron density redistribution upon excitation for both locally excited (LE) and CT transitions (Figures S8-S10), while also allowing the inclusion of solvent effects via the polarizable continuum model (PCM) with state-specific solvation for CT states.

Although TD-DFT describes LE state energies with good accuracy, often in quantitative agreement with experimental observations, it tends to underestimate or overestimate CT state energies, with typical absolute errors ranging from 0.1 to 1.0 eV. To correct this discrepancy, we calculated the gas-phase excitation energies using the approximate coupled-cluster method (CC2) with aug-cc-pVDZ basis set, which offers consistent accuracy for both LE and CT states. In our two-step approach, gas-phase excited-state energies were obtained at the CC2 level, while solvent effects were subsequently incorporated from the TD-DFT calculations. The CC2 and ADC(2) calculations were performed with Turbomole 7.1[3].

Validity of this approach was verified for the lowest intramolecular CT state of amine-substituted Py, where the two-step approach within the linear-response framework for solvent effects reproduced the same solvent shift of -64 meV from the gas-phase excitation energy as obtained with ADC(2) with a COSMO solvent description.

The optical spectra are simulated using vertical gradient (VG) approximation combined with the cumulant expansion formalism[4]. This method is widely employed in the modeling of organic molecules and super-molecular systems and allows us to include vibronic effects on the optical spectra without extensive sampling of the molecular geometries. This approach also makes it possible to combine different levels of theory for the electronic excitations (CC2 approach) and solvent effects (TD-DFT). Within this methodology, the absorption α(ω) and fluorescence I(ω) spectra are obtained as a sum over excited states:

$$\square(\square) \; \square \; \square \; \square_{i} \left| \square_{gi} \right|^{2} D_{i}(\square)$$

$$I(\square) \; \square \; \square^{2} \; \square_{i} p_{i} \left| \square_{gi} \right|^{2} \tilde{D}_{i}(\square)$$

Where p_i corresponds to the Boltzmann factor $p_i = \exp\left[-\frac{\square_i}{k_B T}\right] / \square_j \exp\square\left[-\frac{\square_j}{k_B T}\right]$ and $\square_{gi}$ is the transition dipole moment between the ground and i-th exciton state.

The homogeneous lineshape $D_i(\square)$ and fluorescence lineshape are obtained in the cumulant expansion formalism as:

$$D_{i}(\square) = \square_{-\square}^{\square} e^{-i\left(\square_{gi} - \square\right)t - g_{i}(t)} dt$$

$$\tilde{D}_{i}(\square) = \square_{-\square}^{\square} e^{-i\left(\square_{gi} - \square\right)t + 2i\square_{i} t - g_{i}^{*}(t)} dt$$

where $g_i(t)$ is the lineshape function of the excited state i and $\square_i$ is the corresponding reorganization energy. The lineshape function $g_i(t)$ can be obtained from the spectral density $J_i(\square)$ of the excited state i as:

$$g_{i}(t) = -\square_{0}^{\square} d\square \frac{J_{i}(\square)}{\square\square^{2}} \left[\coth\left(\frac{\square\square\square}{2}\right)(\cos(\square t) - 1) - i(\sin(\square t) - \square t)\right]$$

Spectral densities

The spectral density of the molecules was modeled by two contributions. The first contribution corresponds to the intramolecular vibrations coupled to the electronic excited states and is modeled by an underdamped

Brownian oscillator. The intramolecular normal modes were obtained with normal mode analysis and the corresponding Huang-Rhys factors through the vertical gradient method[4–6]. The structure of the system was optimized with the B3LYP functional and 6-31+G(d,p) basis set with CH_2Cl_2 solvent included through the polarizable continuum model (PCM). At the optimal geometry, the normal mode analysis was performed using the same approach as for the geometry optimization. The gradients of the excited and ground state potential surfaces were computed at the TD-DFT level with the range separated ωB97XD functional, which describes both LE and CT states with reasonable accuracy. The excited state gradients were corrected for the different DFT functional used for geometry optimization by subtracting the non-zero gradient of the ground state. This is within the harmonic approximation, used also for computing the spectral density. The second contribution originates from the nuclear motion of the environment, and is modeled by an overdamped Brownian oscillator. This contribution is not possible to obtain within the PCM approximation and therefore, we used the reorganization energy, which well represents the broadening of the vibronic peaks as well as the fluorescence Stokes shift of the monomers.

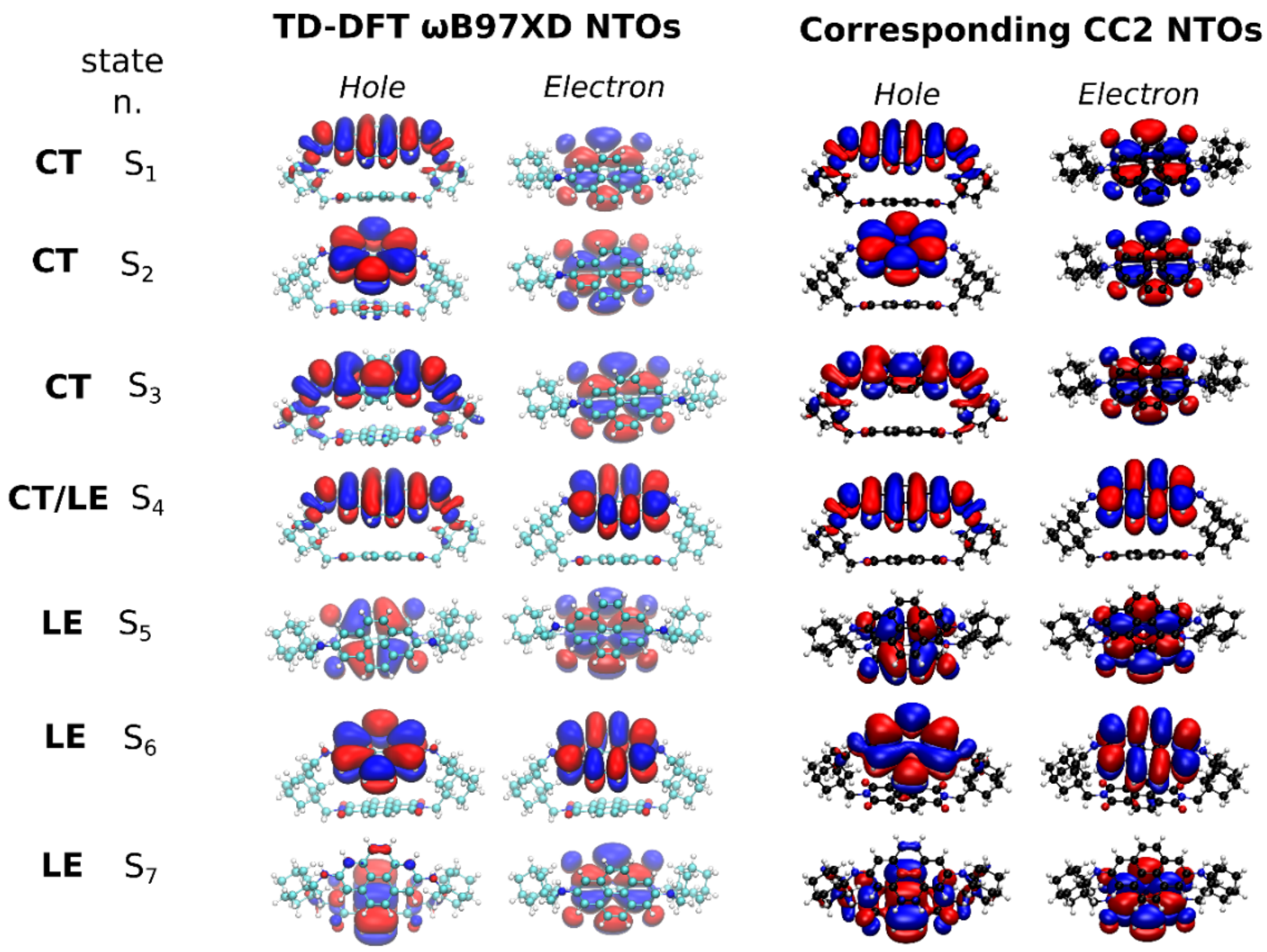

Figure S8. Comparison of the NTO analysis between TD-DFT with ωB97XD functional and the corresponding states from the CC2 method for the excited states of the **NDI-AdaNH-Py** paracyclophane.

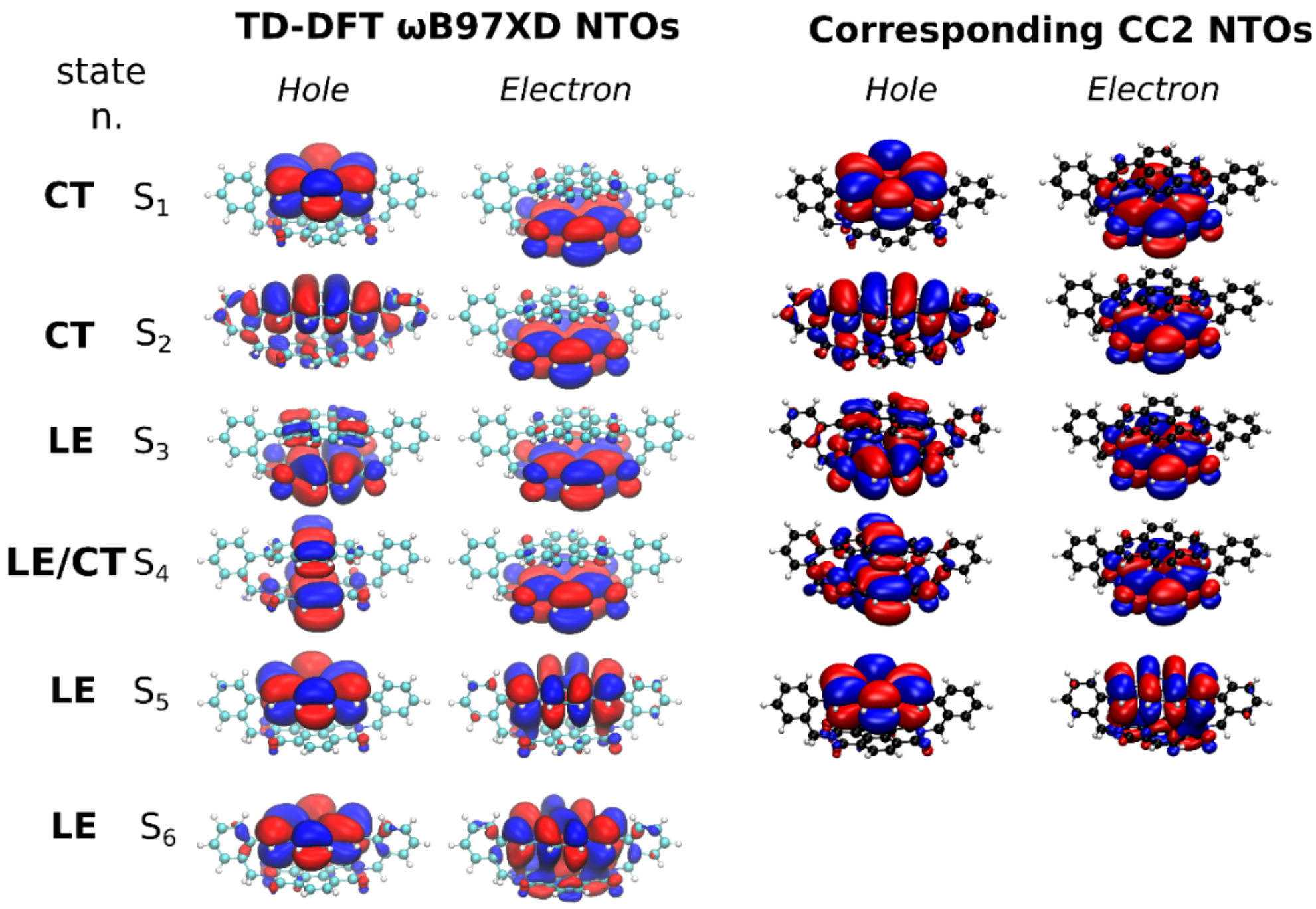

Figure S9. Comparison of the NTO analysis between TD-DFT with ωB97XD functional and the corresponding states from the CC2 method for the excited states of the **NDI-Ph-Py** paracyclophane.

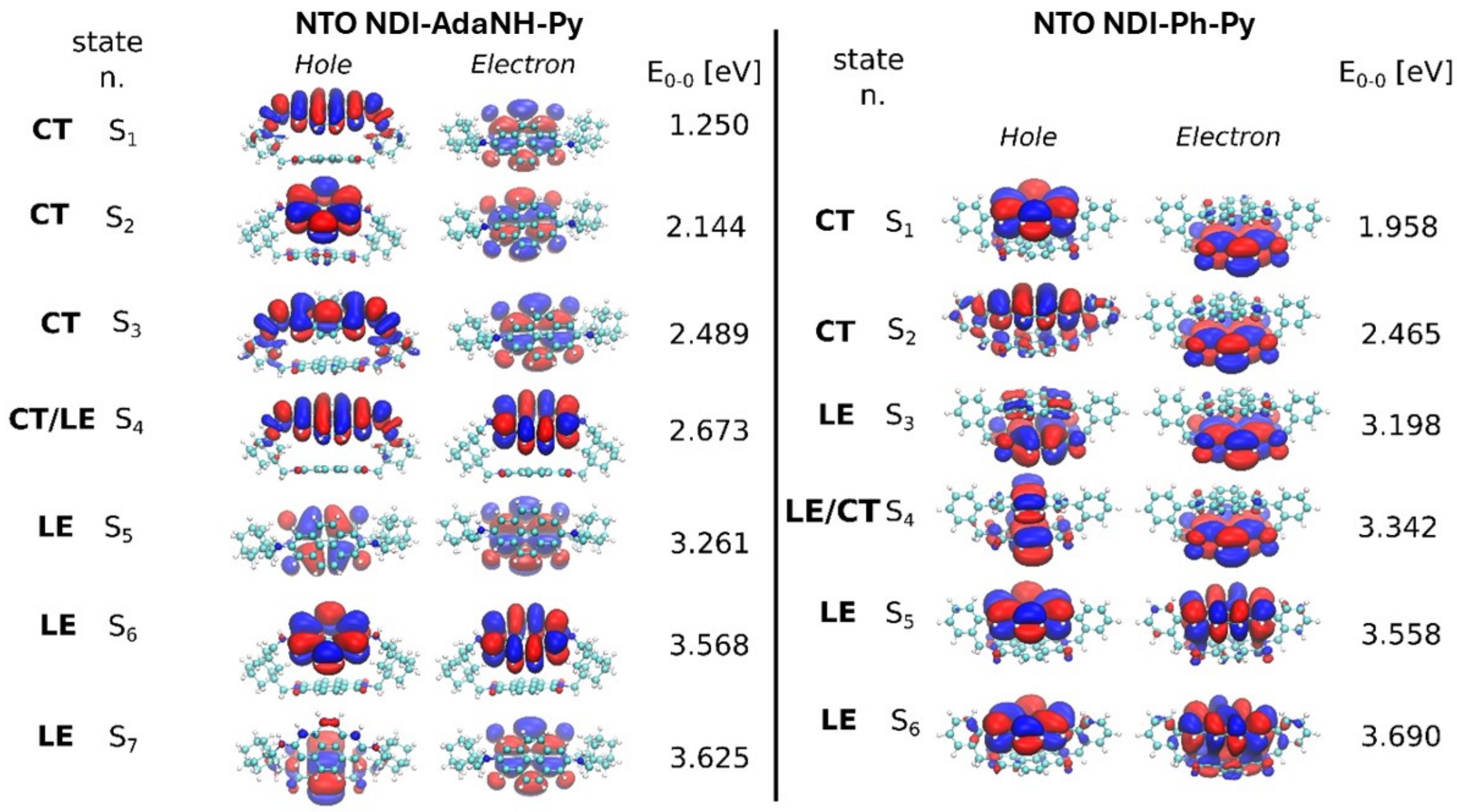

Figure S10. Natural Transition Orbital analysis for **NDI-AdaNH-Py** and **NDI-Ph-Py** together with corresponding adiabatic energies.

Paracyclophanes' excited state and optical properties

The first excited state (S_1) is dominated by electron excitation from the highest occupied molecular orbitals (HOMO) localized on the amine-Py unit to the lowest unoccupied molecular orbitals (LUMO) localized on the NDI core. The slightly lower energy by 12 meV of the CT state for the **NDI-AdaNH-Py** molecule can be attributed to differences in the delocalization of the Py HOMO over the linker (Figure 4), which is consistent with observations in the spectra of the monomeric Py with AdaNH and BnNH linkers found in our previous work[2,7]. Although the S_1 transition corresponds to the low-energy tail of the experimental absorption spectra, the calculated intensity is underestimated by approximately two orders of magnitude. This discrepancy arises from the use of the vertical gradient approach, in which excitation properties are computed at the optimized ground-state geometry. Under these conditions, the S_0 → S_1 transition dipole moment is nearly zero due to molecular symmetry. In solution, however, thermal motion breaks this symmetry, enabling coupling between dark CT and bright LE states, thereby enhancing the transition dipole of the CT state, as seen in the experimental spectra. For the S_2 CT state, the excitation energy for **NDI-BnNH-Py** is red-shifted by 154 meV relative to **NDI-AdaNH-Py**, while in the gas phase, the excitation energies are nearly identical (Table S1). This shift can thus be attributed to different state-specific solvation effects. The same trend is also obtained for the other through-space CT states, suggesting that solvent cavity shape, influenced by the molecular geometry and chromophore separation, affects the solvation shifts of the AdaNH- and BnNH-linked PCPs. Like for S_1, the calculated intensity of the S_2 state is underestimated due to the neglect of thermal fluctuations. The S_4 state (Figure 4), assigned for the amine-linked PCPs, appears red-shifted for **NDI-AdaNH-Py** relative to the **NDI-BnNH-Py** analogue (Figure 3), following the trend previously reported for their reference molecules[2]. This can be attributed to the differences in delocalization of the Py HOMO across the linkers. The computed shift between the two linkers of 216 meV is slightly overestimated relative to the experimental value of 100 meV. This deviation arises mainly from differences in the linear-response solvent shift of 97 meV between the two linkers, suggesting that TD-DFT with linear response solvation tends to overestimate the solvent-shift differences between the **NDI-AdaNH-Py** and **NDI-BnNH-Py**.

Apart from the CT excited state and optical properties discussed in the main text, there are significant differences in LE states, corresponding mainly to NDI or Py excited state units, among the three studied PCPs. The strong absorption band corresponding to the S_5 excited state between 3.1-3.5 eV originates from the lowest excited state of the NDI moiety, while the intense absorption band between 3.5-4.0 eV (S_6) corresponds to the Py ^1L_a transition. Theoretical calculations also predict a higher intensity of the absorption spectra in the S_6 state region (pyrene unit excitation) for the BnNH-linked PCPs compared to the AdaNH-

linked one, consistent with the experimental findings (Figure 3). This enhancement arises from the shorter inter-unit separation in the BnNH-linked PCP, which increases the interaction energy between the Py ^1L_a state and the NDI lowest excited state from 255 cm^{-1} for the AdaNH linker to 293 cm^{-1} for the BnNH linker, resulting in transition dipole moment redistribution between these transitions. Furthermore, due to the spectral overlap with the S_7 state corresponding to the second excited state of the NDI moiety, the increased transition dipole in the BnNH-linked PCP leads to enhanced absorption in the 3.5-4.0 eV spectral region. The small coupling between the NDI and Py units and larger energy separation leads to only small delocalization between the two units, manifested by the transition dipole moments of 2.08 a.u. and 2.07 a.u. for the NDI and Py lowest LE states of **NDI-AdaNH-Py**, respectively, and 2.06 a.u. and 2.17 a.u. for **NDI-BnNH-Py**, which are close to the values for isolated monomers of 2.39 and 2.06 a.u (Table S1). In **NDI-Ph-Py**, however, short separation of the NDI and Py units and the strong interaction between the corresponding transition densities lead to substantial state mixing and exciton formation, resulting in transition dipole strength redistribution: the NDI LE state dipole decreases to 1.32 a.u., while that of the Py LE state increases to 2.67 a.u (Table S1). The coupling between the lowest LE states of the NDI and Py obtained from diabatization of the dimer states into the monomeric basis is 668 cm^{-1}, closely matching the 660 cm^{-1} coupling derived from localized fragments (Figure S11) using Poisson-TrEs method[8]. This coupling is more than 2.6 times larger than the coupling for the **NDI-AdaNH-Py**, 255 cm^{-1} and 259 cm^{-1}, respectively. The sum of the squares of the transition dipole moments remains nearly constant for both Ada and Ph linkers, confirming that the observed changes result from exciton formation and redistribution of the transition dipole strength between NDI and Py units. Finally, a small redshift of about 75 meV is observed for the first vibronic peak of the NDI absorption around 3.2 eV in **NDI-Ph-Py** (Figure S12). This shift originates from partial delocalization between the NDI LUMO and the Py HOMO orbitals, further supporting the enhanced interaction enabled by the Ph linker's reduced spatial separation.

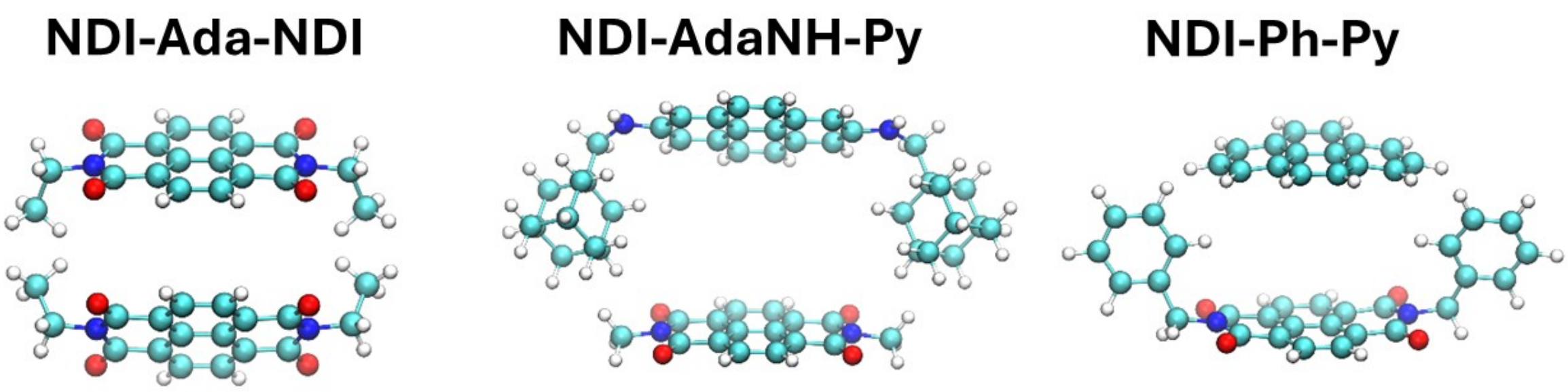

Figure S11. Individual fragments for the calculation of the couplings between transition densities and molecular orbital overlaps for the homo- and hetero-PCPs.

NDI-Ph-Py NDI-AdaNH-Py NDI-BnNH-Py

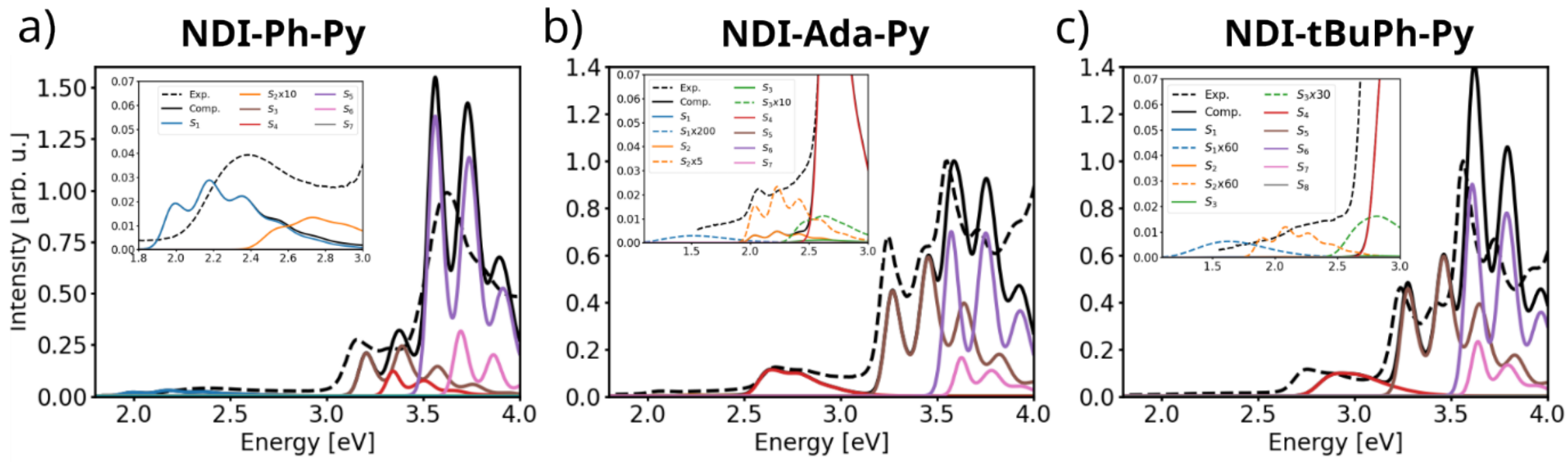

Figure S12. Absorption spectra decomposed into the individual transitions for the three different PCPs.

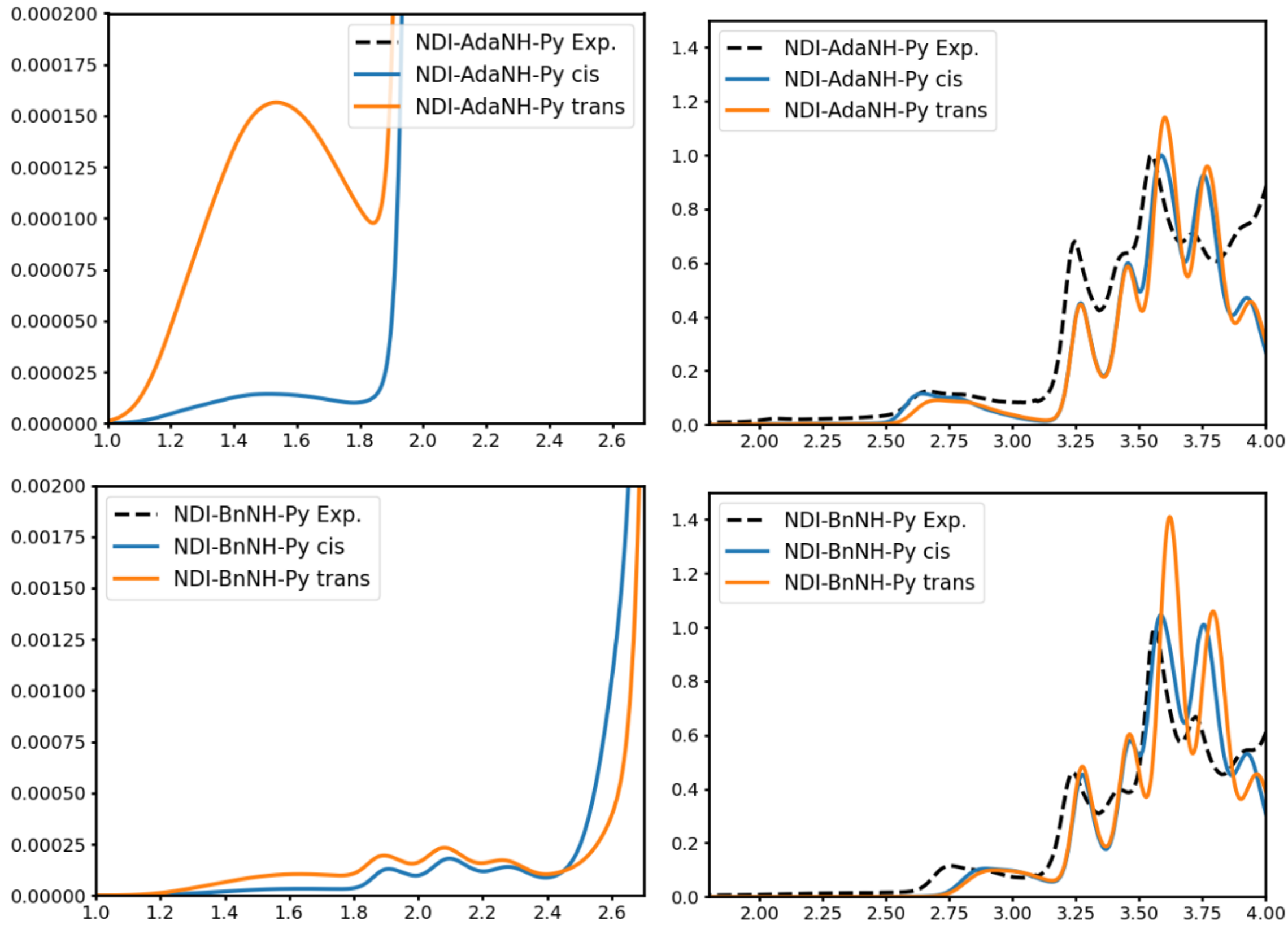

Figure S13. Absorption spectra for *cis* and *trans* conformers of AdaNH and BnNH linkers. There is only a very small difference in the absorption spectra between the two conformers. The main difference is in Py S_1 transition around 3.6 eV and the intensities of the lowest energy CT states.

Table S1. A series of parameters of hole-electron analysis and calculated transition dipole and excitation energy decomposition in the gas phase for the PCPs. The percentage contribution of the MO transition is based on TD-DFT calculation with wB97XD functional and 6-31+G(d,p) basis set. While the main MO contributions to the excitations are reliably determined, the exact percentage contribution is strongly dependent on the DFT functional or method used for calculation of the excited states and starting orbitals for the linear response or perturbative approach.

**NDI-AdaNH-Py** (*cis*)						
State	Gas-phase vertical excitation energy CC2 (eV)	Solvent shift (eV)	Reorganization Energy (eV)	Adiabatic energy E_{0-0} (eV)	Transition dipole moment (a.u.)	Major contribution
S_1	1.879	-0.315	-0.439	1.125	0.020	99% HOMO → LUMO
S_2	2.613	-0.282	-0.311	2.020	0.260	96% H-1 → LUMO
S_3	2.991	-0.386	-0.304	2.301	0.124	95% H-2 → LUMO
S_4	2.822	-0.057	-0.170	2.595	1.017	92% HOMO → L+1
S_5	3.589	-0.067	-0.268	3.245	2.076	97% HOMO-3→ LUMO
S_6	3.830	-0.053	-0.209	3.568	2.069	85% H-1 → L+1 11% HOMO → L+5
S_7	3.832	-0.069	-0.138	3.625	0.836	56% H-11→ LUMO 18% H-6→LUMO 11% H-5→LUMO 7% H-3→L+3
**NDI-BnNH-Py** (*trans*)						
State	Gas-phase vertical excitation energy CC2 (eV)	Solvent shift (eV)	Reorganization Energy (eV)	Adiabatic energy E_{0-0} (eV)	Transition dipole moment (a.u.)	Major contribution
S_1	2.093	-0.424	-0.532	1.137	0.055	99% HOMO → LUMO
S_2	2.642	-0.456	-0.320	1.866	0.057	99% H-1 → LUMO
S_3	3.257	-0.385	-0.265	2.607	0.085	93% H-2 → LUMO
S_4	2.981	0.040	-0.210	2.811	0.959	90% HOMO → L+1
S_5	3.461	0.073	-0.265	3.269	2.064	87% H-7→ LUMO 6% H-5→ LUMO
S_6	3.695	0.094	-0.185	3.604	2.173	79% H-1 → L+1 6% H-9 → LUMO
S_7	3.717	0.041	-0.120	3.638	0.930	70% H-9→ LUMO 10% H-10→LUMO 7% H-1→L+1 6% H-7→L+3
**NDI-Ph-Py**						

State	Gas-phase vertical excitation energy CC2 (eV)	Solvent shift (eV)	Reorganization Energy (eV)	Adiabatic energy $E_{0\text{-}0}$ (eV)	Transition dipole moment (a.u.)	Major contribution
S_1	2.504	-0.235	-0.311	1.958	0.671	99% HOMO → LUMO
S_2	2.999	-0.189	-0.345	2.465	0.137	96% H-1 → LUMO
S_3	3.454	-0.017	-0.239	3.198	1.321	59% H-2→ LUMO 31% H-4→ LUMO 8% HOMO → L+1
S_4	3.543	-0.069	-0.132	3.342	0.739	56% H-7→ LUMO 28% H-8→ LUMO
S_5	3.805	-0.067	-0.180	3.558	2.672	68% HOMO→ L+1 7% H-1→ L+1 7% HOMO → L+2
S_6	3.822	-0.010	-0.122	3.690	1.076	33% H-1→ L+1 20% HOMO→ L+2 17% HOMO → L+4 15% HOMO→L+1

Table S2. Transition dipole moments for the lowest CT transitions of **NDI-AdaNH-Py** sampled along ab-initio molecular dynamics at 300 K. The average transition dipole for the lowest CT state is almost two orders of magnitude larger than the one for the optimal geometry. Compared to the S_4 (CT/LE) transition dipole strength $\left|\square_{S_4}\right|^2 = 1.034$ the intensity of the CT transitions should be 60x and 20x lower for the CT1 and CT2, respectively, which roughly agrees with the experimental absorption intensity.

**NDI-AdaNH-Py** (*cis*)		
frame	Transition dipole moment CT_1 $\left|\square_{S_1}\right|^2$ (a.u.)	Transition dipole moment CT_2 $\left|\square_{S_2}\right|^2$ (a.u.)
1	0.046	0.055
2	0.0188	0.0388
3	0.0109	0.0846
4	0.0113	0.1186
5	0.0158	0.1137
6	0.0176	0.091
7	0.0117	0.06
8	0.0233	0.0369
9	0.0522	0.0038
10	0.0308	0.035
11	0.0039	0.0567
12	0.0118	0.0591
13	0.012	0.0945
14	0.0061	0.0445
15	0.0021	0.0237
16	0.0051	0.0136
17	0.0055	0.0403
18	0.0018	0.0409

19	0.0001	0.0074
20	0.0036	0.024
21	0.0107	0.0497
22	0.0216	0.0372
23	0.0761	0.0234
mean	**0.0173**	**0.0501**
STD	**0.0185**	**0.0317**

Transition density of the CT state for NDI-Ph-Py

Although the S_1 state is similar in character to the S_2 state in **NDI-AdaNH-Py** and **NDI-BnNH-Py**, its transition dipole moment is approximately an order of magnitude larger. This enhancement does not originate from mixing with nearby LE states, but rather from the substantially shorter D-A spatial separation, which increases orbital overlap. This is supported by the theoretical calculations, where the diabatization analysis using the Fragment Charge Difference (FCD) algorithm[9] shows that the couplings between the CT state and the two lowest excited states of the NDI are 0.34 cm^{-1} and 0.84 cm^{-1}, while the couplings to the two lowest excited states of pyrene are 0.29 cm^{-1} and 0.44 cm^{-1}. These small coupling values, together with the large energy gap between CT and LE states, indicate the absence of delocalization and transition dipole moment redistribution. Nevertheless, the shorter spatial separation in **NDI-Ph-Py** (3.7 Å) results in a significantly larger HOMO-LUMO overlap (0.039), compared to **NDI-AdaNH-Py** (0.008), where the chromophores are separated by 5.44 Å (Figure 2). Hence, the enhanced orbital overlap between the HOMO of the pyrene unit and LUMO of the NDI unit in NDI-AdaNH-Py leads to partial delocalization between these MOs and results in enhancement of the CT state dipole moment. In the simplified picture, the resulting HOMO of the **NDI-Ph-Py** can be expressed as:

$$\psi_{HOMO}(r) \approx c_1 \psi^{Pyr}_{HOMO} + c_2 \psi^{NDI}_{LUMO}$$

where ψ^{Pyr}_{HOMO} and ψ^{NDI}_{LUMO} are HOMO and LUMO orbitals of the individual separated units and c_i are expansion coefficients satisfying $c_1 \gg c_2$ which represent only small delocalization of the corresponding states and major contribution of the Pyrene HOMO orbital to the MO of the paracyclophane. The same is also true for the LUMO orbital of the **NDI-Ph-Py**, which can be expressed as

$$\psi_{LUMO}(r) \approx -c_2 \psi^{Pyr}_{HOMO} + c_1 \psi^{NDI}_{LUMO}$$

which is mostly localized on the NDI unit. The resulting transition density of the lowest CT state S1 can therefore be approximated as:

$$\square_{ge}^{(S_1)}(r) = c_2 c_1 \left(\square_{LUMO}^{NDI}(r) - \square_{HOMO}^{Pyr}(r) \right) + \square_{HOMO}^{Pyr}(r) \square_{LUMO}^{NDI}(r)(c_1^2 - c_2^2)$$

In the case of small overlap S and therefore small delocalization, we can assume $c_1 \square 1$ and $c_2 \square \square S$. In this case, the second term with HOMO and LUMO orbital overlap with scaling as S becomes negligible and only the first term with scaling $\square S$ prevails, yielding the final approximation for the transition density as:

$$\square_{ge}^{(S_1)}(r) \square c_2 c_1 \left(\square_{LUMO}^{NDI}(r) - \square_{HOMO}^{Pyr}(r) \right)$$

Such transition density can be interpreted as negative charge density in the NDI LUMO orbital and positive charge density in the pyrene HOMO orbital. This leads to the transition dipole moment oriented in a perpendicular direction to the NDI and Pyrene planes. The iso-surface of the transition density is plotted in Figure S14.

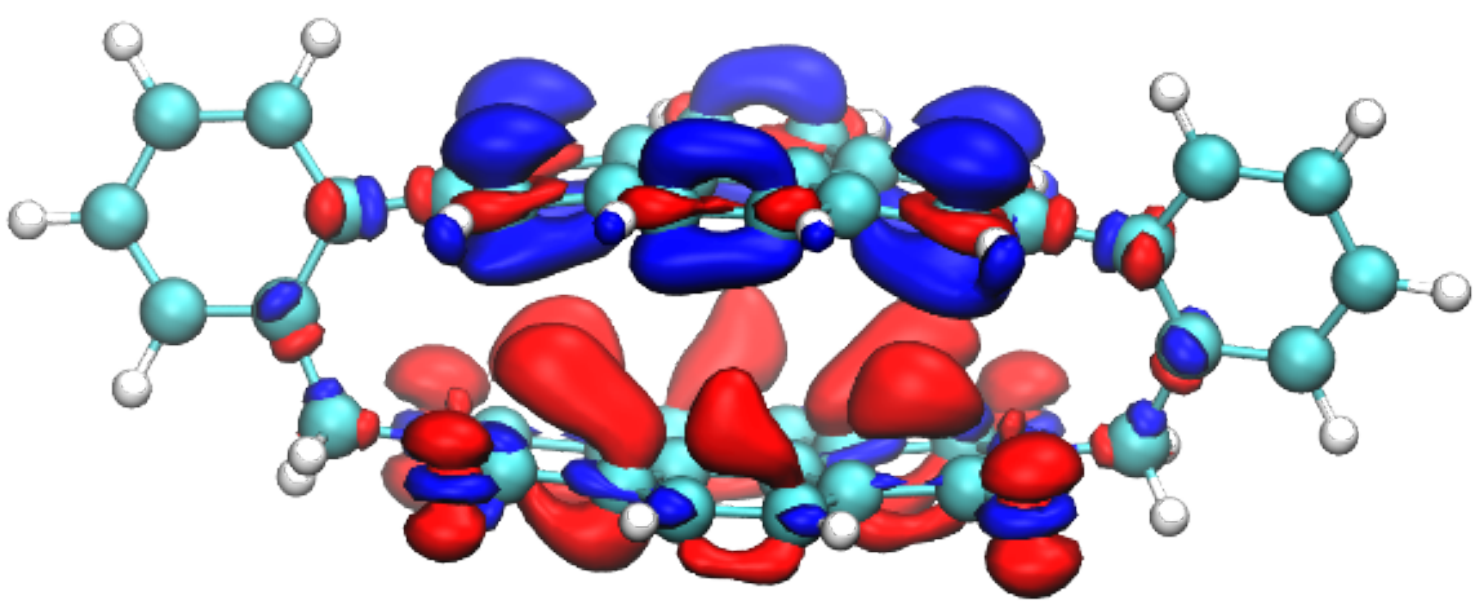

Figure S14. Iso-surface representation of the transition density of the **NDI-Ph-Py** lowest CT state S_1. The positive part (red) of the transition density is localized on the NDI unit, whereas the negative part (blue) is localized on pyrene. This leads to the transition dipole moment oriented in a perpendicular direction to the NDI and Pyrene planes.

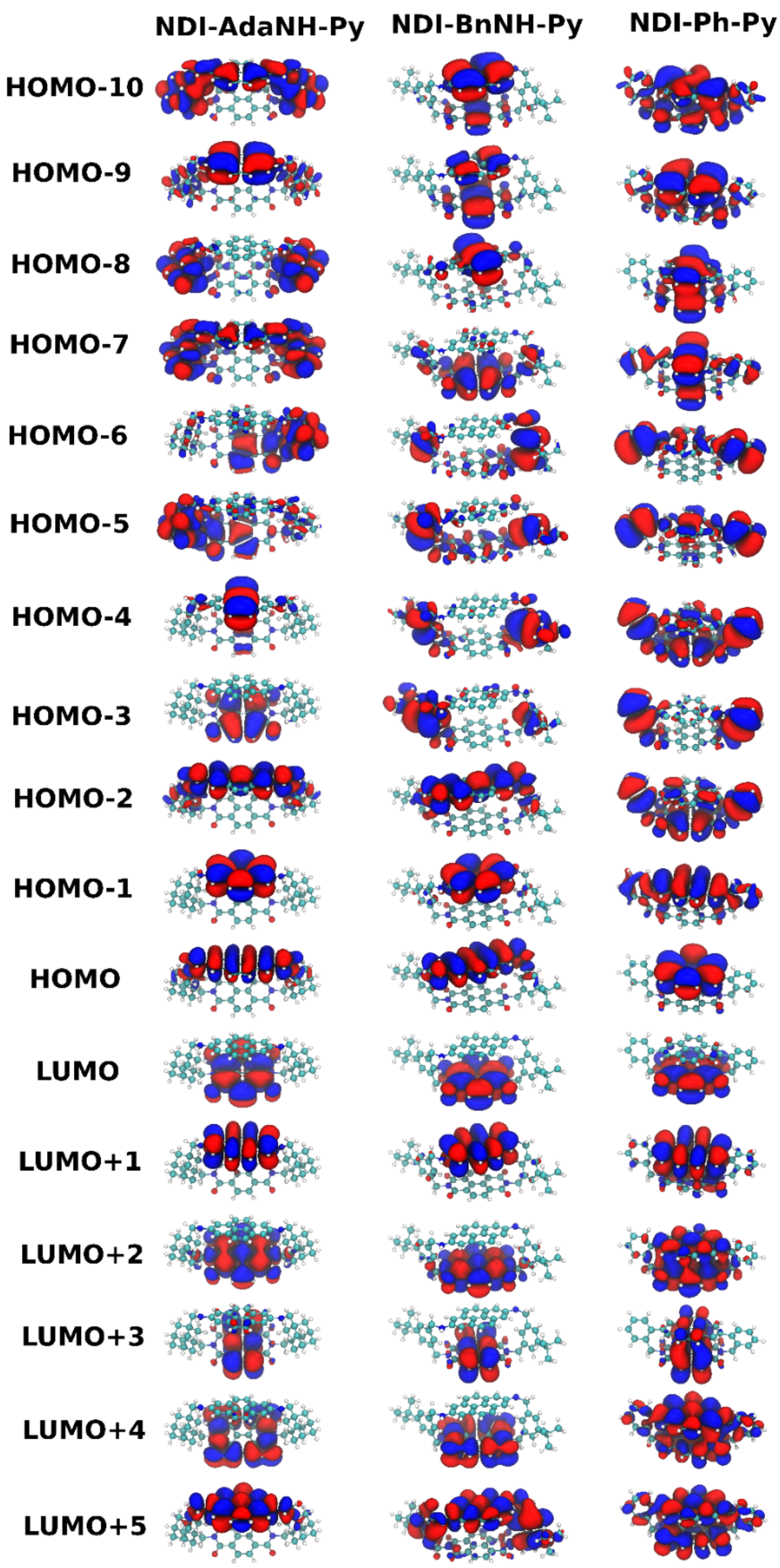

Figure S15. Molecular orbitals (MO) of three hetero-PCPs calculated at the CC2 level.

Electrochemical properties

Including the solvent effects is crucial for obtaining a quantitative description of the redox properties, because it can augment to a change of around 1 eV in the energies of the charged systems compared to the gas-phase

results (Table S2). Moreover, structural relaxation during the redox reactions leads to the shift of the HOMO orbitals to higher energy; meanwhile, the LUMO energy is lowered, reducing the band gap by around 0.2-0.4 eV.

Table S3. Comparison of different approaches for computing the redox properties. For the vertical ionization potential (IP) and electron affinity (EA), the geometry of the system is kept fixed at the neutral-charged system, while for the adiabatic approach, the geometry was optimized for every charged state separately. The calculations, including the solvent effects, were performed with the polarization continuum model (PCM).

Compounds	Vertical gas-phase			Vertical PCM			Adiabatic PCM		
	$-IP_{comp}$ (eV)	$-EA_{comp}$ (eV)	$E_{g,comp}$ (eV)	$-IP_{comp}$ (eV)	$-EA_{comp}$ (eV)	$E_{g,comp}$ (eV)	$-IP_{comp}$ (eV)	$-EA_{comp}$ (eV)	$E_{g,comp}$ (eV)
NDI-Ada-NDI				-7.13	-3.68	3.44	-7.07	-3.81	3.79
NDI-Bn-NDI				-6.81	-3.76	3.06	-6.73	3.85	2.88
NDI-AdaNH-Py				-4.97	-3.53	1.45	-4.81	-3.68	1.13
4 (Pyrene-Ada)	-6.20	-0.31	5.89	-5.05	1.75	3.31	-4.82	-1.94	2.88
Pyrene-*t*BuPh	-6.34	-0.38	5.96	-5.18	-1.79	3.39	-4.98	-1.92	3.06
NDI-Ada	-8.0	-2.31	5.68	-7.13	-3.68	3.44	-6.81	-3.70	3.11
7 (NDI-*t*BuPh)	-7.72	-2.45	5.27	-6.81	-3.76	3.06	-6.66	-3.80	2.86

Transient absorption spectroscopy

Setup

Femtosecond transient absorption (TA) measurements were performed using a home-built broadband pump-probe setup. Ultrashort pulses were generated by a Ti:Sapphire amplifier system operating at a repetition rate of 4 kHz with a pulse duration of ∼80 fs. The 266 nm pump pulses were produced by third-harmonic generation of the 800 nm fundamental in BBO crystals. A white-light continuum probe was generated by focusing a small fraction of the 800 nm beam onto a CaF_2 crystal, providing broadband coverage in the 350-720 nm range. Pump and probe beams were focused onto the sample, and the transmitted probe light was detected with a CMOS array detector. Pump-probe time delays were varied up to 2 ns using a motorized delay stage. All samples were measured in a static 1 mm path length quartz cuvette. Global analysis was performed applying a sequential kinetic model (A → B → C → D) to extract the Species-Associated Spectra (SAS) and the corresponding time constants.

Reference Compounds

Reference compounds **AdaNHBoc-Py**, **BnNHBoc-Py**, and **PhNHBoc-Py** were measured under identical conditions to establish the spectral fingerprints of the individual chromophores. Upon 266 nm excitation, the pyrene moiety in both **AdaNHBoc-Py** and **BnNHBoc-Py** is promoted to a high-lying singlet excited state (^{n}Py$^{\square}$), followed by rapid internal conversion (IC) to the lowest pyrene singlet state (^{1}Py∗). The two compounds exhibit similar transient spectra, characterized by a broad ESA band spanning ∼430-650 nm with a maximum around 560 nm, which we assign to the S_1 excited state of the amine-functionalized pyrene chromophore. The red-shifted position of this ESA band relative to unsubstituted pyrene is consistent with the electron-donating character of the secondary amine substituents introduced at the 2,7-positions of the pyrene core[10,11]. Such donor substitution modifies the pyrene frontier orbitals and low-lying excited-state electronic structure, as shown by DFT calculations in the main text. Global analysis of **AdaNHBoc-Py** yields four time constants: $\square_1$ = 2.36 ps, $\square_2$ = 30.91 ps, $\square_3$ = 318 ps, and $\square_4$ = 6.6 ns. For **BnNHBoc-Py**, three components are sufficient: $\square_1$ = 0.30 ps, $\square_2$ = 42.77 ps, and $\square_3$ = 2.2 ns. In both cases, the fastest component ($\square_1$) is assigned to the early relaxation of an initially hot pyrene-centered singlet state. Because excitation at 266 nm may populate higher-lying singlet states, this component may include relaxation through higher excited states, internal conversion, vibrational cooling, and structural relaxation within the singlet excited-state manifold. The intermediate components ($\square_2$ and $\square_3$ for **AdaNHBoc-Py**) are assigned to further spectral relaxation within this manifold. The final resolved species is attributed to the relaxed pyrene S_1 state. Since the excited-state signal does not necessarily return fully to the baseline within the measured time window, this final species and its associated time constant should be regarded as the last resolved excited-state contribution rather than direct evidence for complete ground-state recovery. A minor contribution from intermolecular excimer formation cannot be fully excluded, since pyrene excimer absorption has been reported at higher concentrations[12,13]. However, under the present measurement conditions, excimer formation is expected to be limited, and the observed ESA band is therefore primarily assigned to the monomeric pyrene-centered ^{1}Py∗ state. The TAS and SAS of **AdaNHBoc-Py** and **BnNHBoc-Py** are shown in Figure S16.

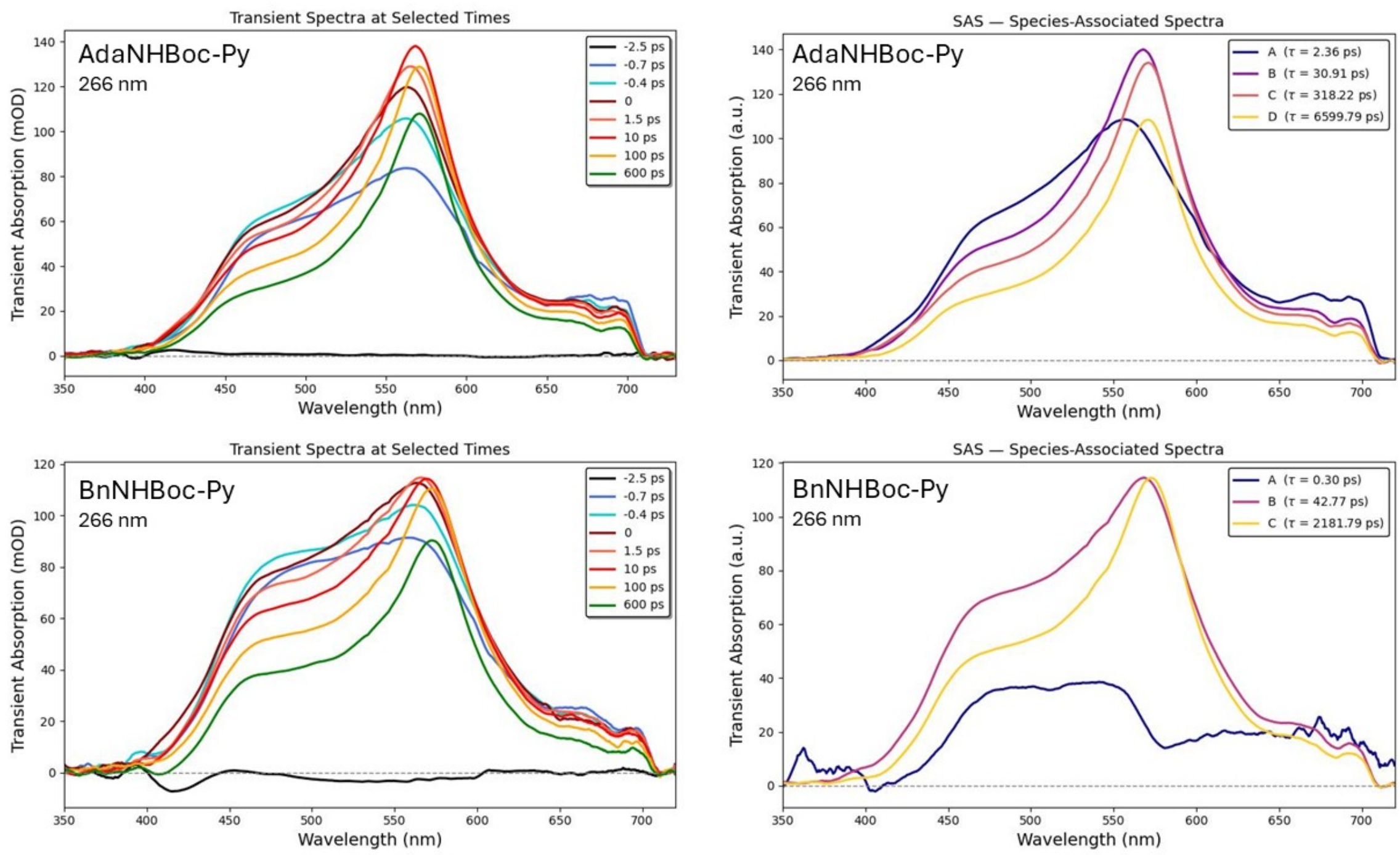

Figure S16. TA spectra and SAS of reference compounds **AdaNHBoc-Py** and **BnNHBoc-Py**, excited at 266 nm.

The transient absorption of **PhNHBoc-Py** differs from that of the amine-functionalized references. Upon 266 nm excitation, the ESA band peaks at ∼455 nm, significantly blue-shifted relative to **AdaNHBoc-Py** and **BnNHBoc-Py** (∼560 nm). This position is closer to the reported S_1 ESA of unsubstituted pyrene, suggesting that **PhNHBoc-Py** exhibits a more pyrene-like excited-state electronic structure[13–15]. In contrast, the amine substituents in **AdaNHBoc-Py** and **BnNHBoc-Py** are expected to act as stronger electron donors and to perturb the pyrene frontier orbitals more substantially, leading to a red-shifted ESA response[13,16]. Global analysis yields four time constants: τ$_1$ = 0.30 ps, τ$_2$ = 20.90 ps, τ$_3$ = 296 ps, and τ$_4$ = 1.6 ns. The fastest component (τ$_1$) is assigned to early relaxation of an initially hot pyrene singlet state following high-energy excitation at 266 nm. This component may include relaxation from higher-lying singlet states, internal conversion, and vibrational cooling, rather than pure S_2 → S_1 internal conversion alone[13,17]. The subsequent evolution is attributed to continued relaxation within the singlet excited-state manifold. This may involve vibrational cooling and structural reorganization toward the relaxed S_1 geometry. The longest-lived component (τ$_4$ = 16 ns) is assigned to the relaxed pyrene singlet state. The TAS and SAS of **PhNHBoc-Py** are shown in Figure S17.

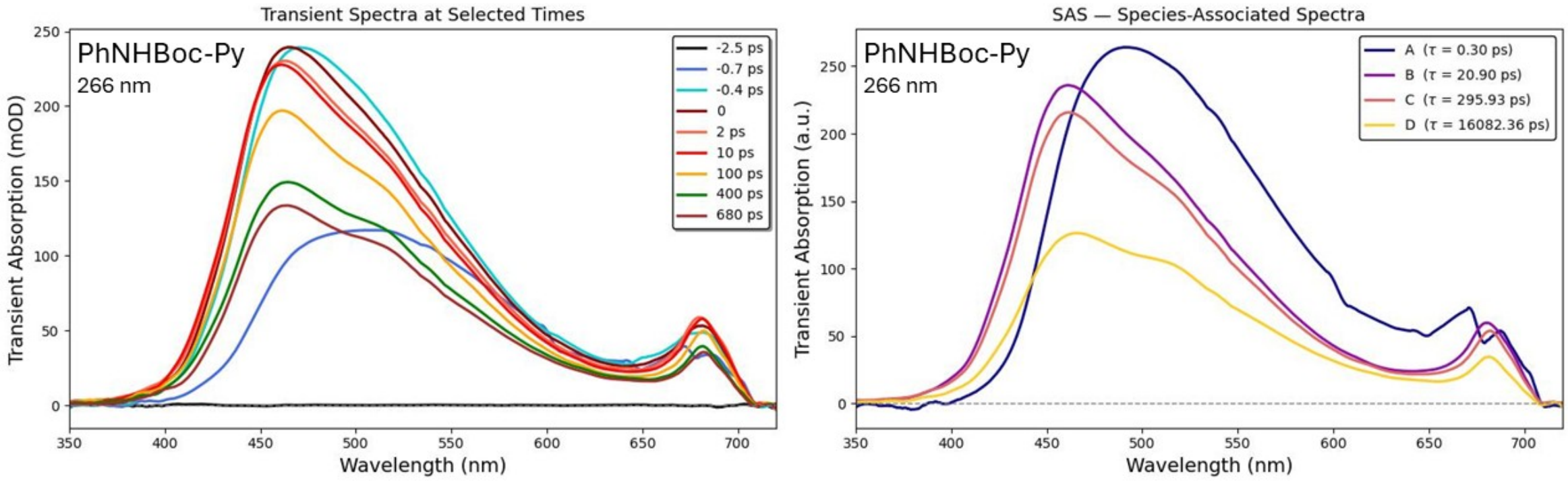

Figure S17. TA spectra and SAS of reference compound **PhNHBoc-Py**, excited at 266 nm.

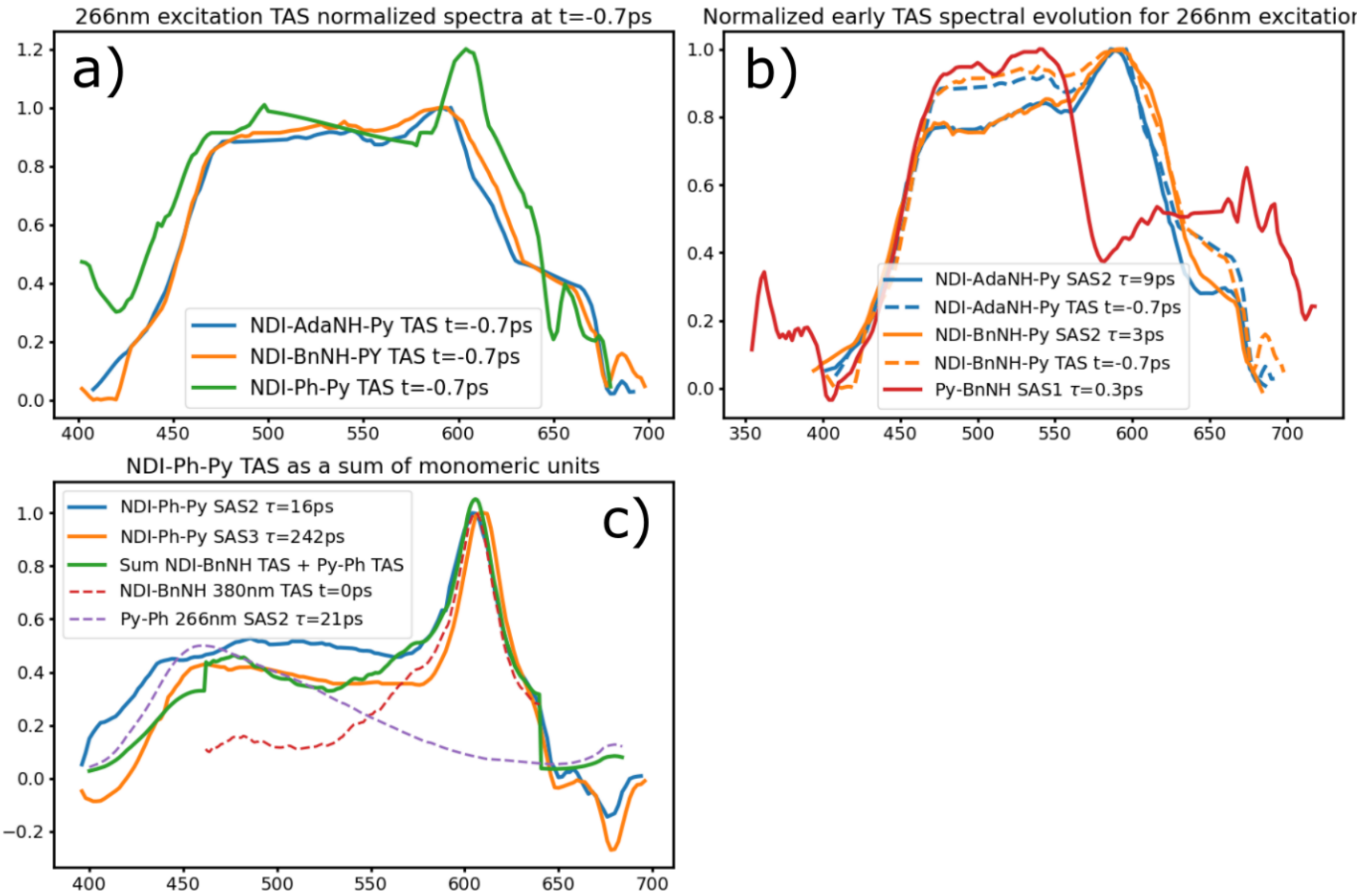

Figure S18. Additional analysis of the TA spectra excited at 266 nm. **a)** Comparison of the early time evolution of the normalized TA spectra of the three PCPs excited at 266 nm. Spectra show large similarities among systems with different linkers, suggesting similar initial population of locally excited states by the pump-pulse. **b)** Comparison of early time evolution of the PCPs TA and SAS excited at 266nm with the early spectral signatures of the Py with the linker. Early time spectra of the Py match well with the observed spectral changes in the PCPs. **c)** SAS spectral signature of the **NDI-Ph-Py** at later times compared to spectra of the NDI and Py reference compounds and the rescaled sum of the spectra of the reference compounds.

References

[1] M. J. Frisch, G. W. Trucks, H. B. Schlegel, G. E. Scuseria, M. A. Robb, J. R. Cheeseman, G. Scalmani, V. Barone, G. A. Petersson, H. Nakatsuji, X. Li, M. Caricato, A. V Marenich, J. Bloino, B. G. Janesko, R. Gomperts, B. Mennucci, H. P. Hratchian, J. V Ortiz, A. F. Izmaylov, J. L. Sonnenberg, D. Williams-Young, F. Ding, F. Lipparini, F. Egidi, J. Goings, B. Peng, A. Petrone, T. Henderson, D. Ranasinghe, V. G. Zakrzewski, J. Gao, N. Rega, G. Zheng, W. Liang, M. Hada, M. Ehara, K. Toyota, R. Fukuda, J. Hasegawa, M. Ishida, T. Nakajima, Y. Honda, O. Kitao, H. Nakai, T. Vreven, K. Throssell, J. A. Montgomery Jr., J. E. Peralta, F. Ogliaro, M. J. Bearpark, J. J. Heyd, E. N. Brothers, K. N. Kudin, V. N. Staroverov, T. A. Keith, R. Kobayashi, J. Normand, K. Raghavachari, A. P. Rendell, J. C. Burant, S. S. Iyengar, J. Tomasi, M. Cossi, J. M. Millam, M. Klene, C. Adamo, R. Cammi, J. W. Ochterski, R. L. Martin, K. Morokuma, O. Farkas, J. B. Foresman and D. J. Fox, 2016, Revision C.01, Gaussian, Inc., Wallingford CT.

[2] V. Slama, C. Negrete-Vergara, E. Zyaee, S. Decurtins, H. M. Frey, T. Feurer, S.-X. Liu and U. Rothlisberger, arXiv, 2026, preprint, arXiv:2602.14114v1, https://doi.org/10.48550/arXiv.2602.14114

[3] Turbomole V7.1, a development of University of Karlsruhe and Forschungszentrum Karlsruhe GmbH, TURBOMOLE GmbH, since 2007, https://www.turbomole.org.

[4] E. Cignoni, V. Slama, L. Cupellini and B. Mennucci, J. Chem. Phys., 2022, **156**, 120901.

[5] M. K. Lee, P. Huo and D. F. Coker, Annu. Rev. Phys. Chem., 2016, **67**, 639–668.

[6] F. J. A. Ferrer and F. Santoro, Phys. Chem. Chem. Phys., 2012, **14**, 13549–13563.

[7] V. Slama, U. Rothlisberger, Chimia, 2026, **80**, 238–241.

[8] J. Adolphs and T. Renger, Biophys. J., 2006, **91**, 2778–2797.

[9] M. Nottoli, S. Jurinovich, L. Cupellini, A. T. Gardiner, R. Cogdell and B. Mennucci, Photosynth. Res., 2018, **137**, 215–226.

[10] J. Merz, J. Fink, A. Friedrich, I. Krummenacher, H. H. Al Mamari, S. Lorenzen, M. Haehnel, A. Eichhorn, M. Moos, M. Holzapfel, H. Braunschweig, C. Lambert, A. Steffen, L. Ji and T. B. Marder, Chem. Eur. J, 2017, **23**, 13164–13180.

[11] A. G. Crawford, A. D. Dwyer, Z. Liu, A. Steffen, A. Beeby, L. O. Pålsson, D. J. Tozer and T. B. Marder, J. Am. Chem. Soc., 2011, **133**, 13349–13362.

[12] C. R. Goldschmidt and M. Ottolenghi, J. Phys. Chem., 1970, **74**, 2041–2042

[13] P. Foggi, L. Pettini, I. Sknta, R. Righini, S. Califano and L. E. Fermi, J. Phys. Chem. A, 1995, **99**, 7439–7445.

[14] P. Foggi, S. Corti, R. Righini and S. Califano, AIP Conf. Proc., 1996, **364**, 169–174.

[15] F. V. R. Neuwahl and P. Foggi, Laser Chemistry, 1999, **19**, 375–379.

[16] K. Basuroy, J. de J. Velazquez-Garcia, D. Storozhuk, D. J. Gosztola, S. T. Veedu and S. Techert, J. Chem. Phys., 2021, **155**, 1–12.

[17] M. Raytchev, E. Pandurski, I. Buchvarov, C. Modrakowski and T. Fiebig, J.Phys. Chem. A, 2003, **107**, 4592–4600.

NMR spectra

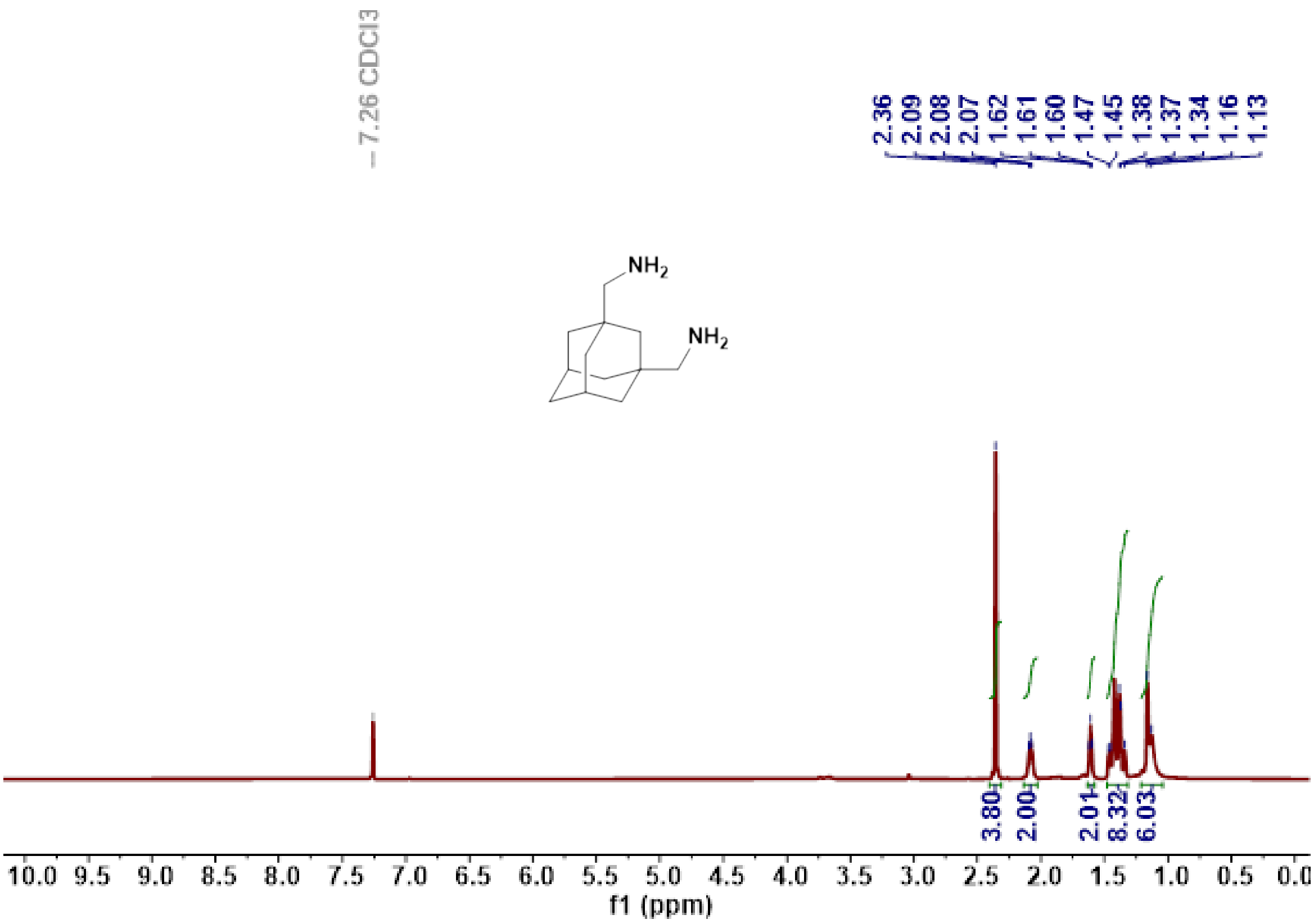

Figure S19. ^{1}H-NMR spectrum of Ada-NH_2.

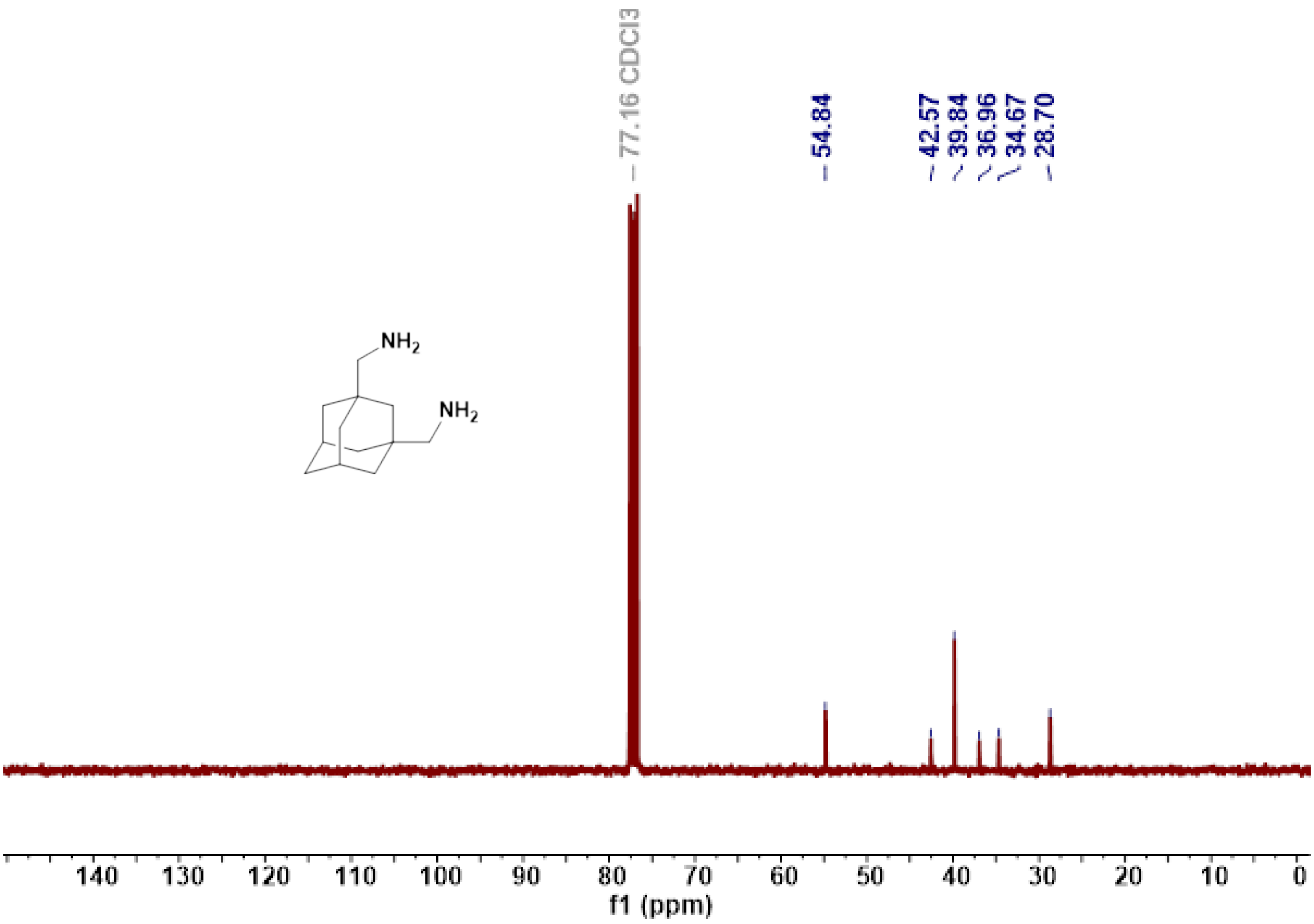

Figure S20. ^{13}C-NMR spectrum of Ada-NH_2.

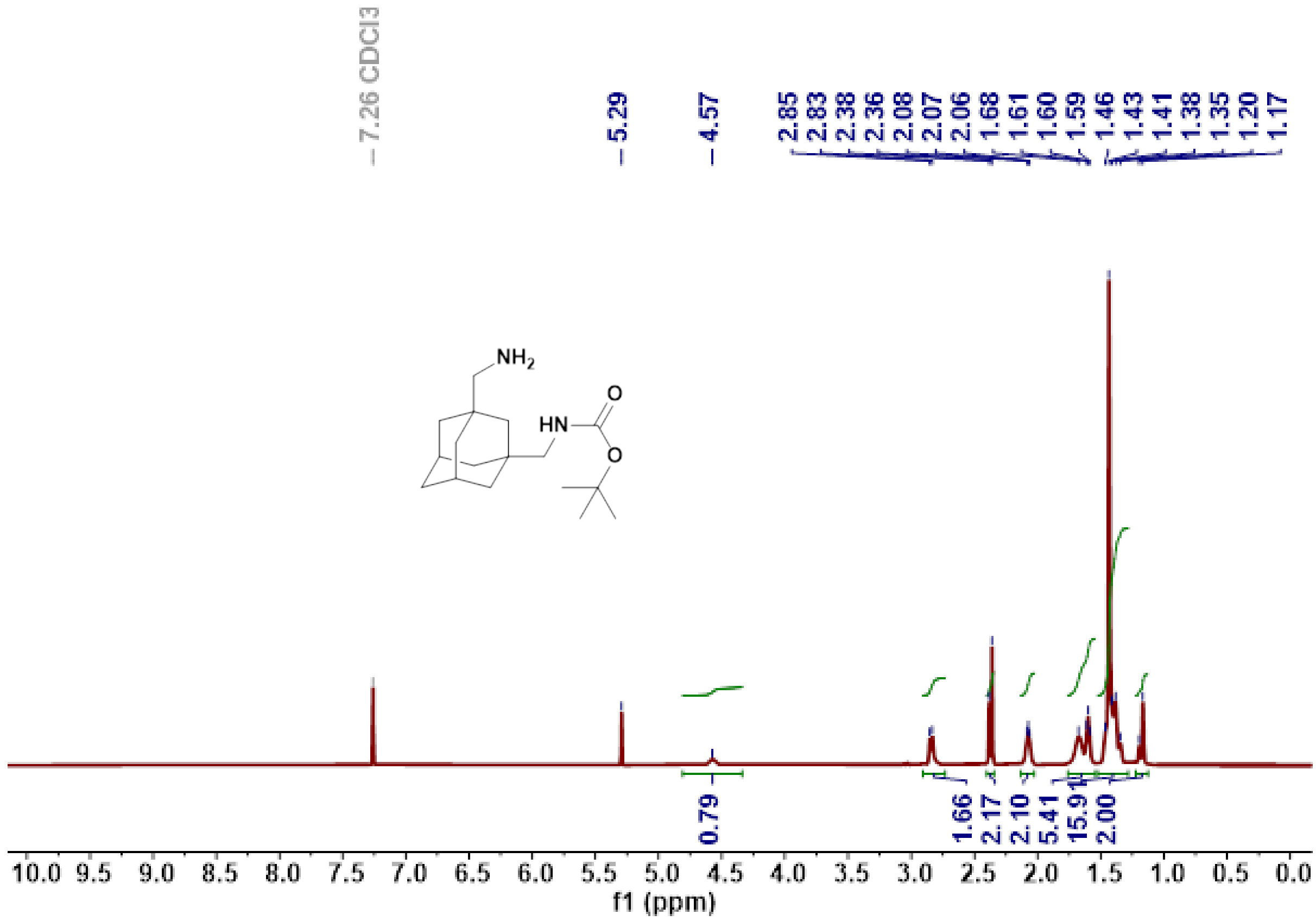

Figure S21. ^{1}H-NMR spectrum of Ada-NHBoc.

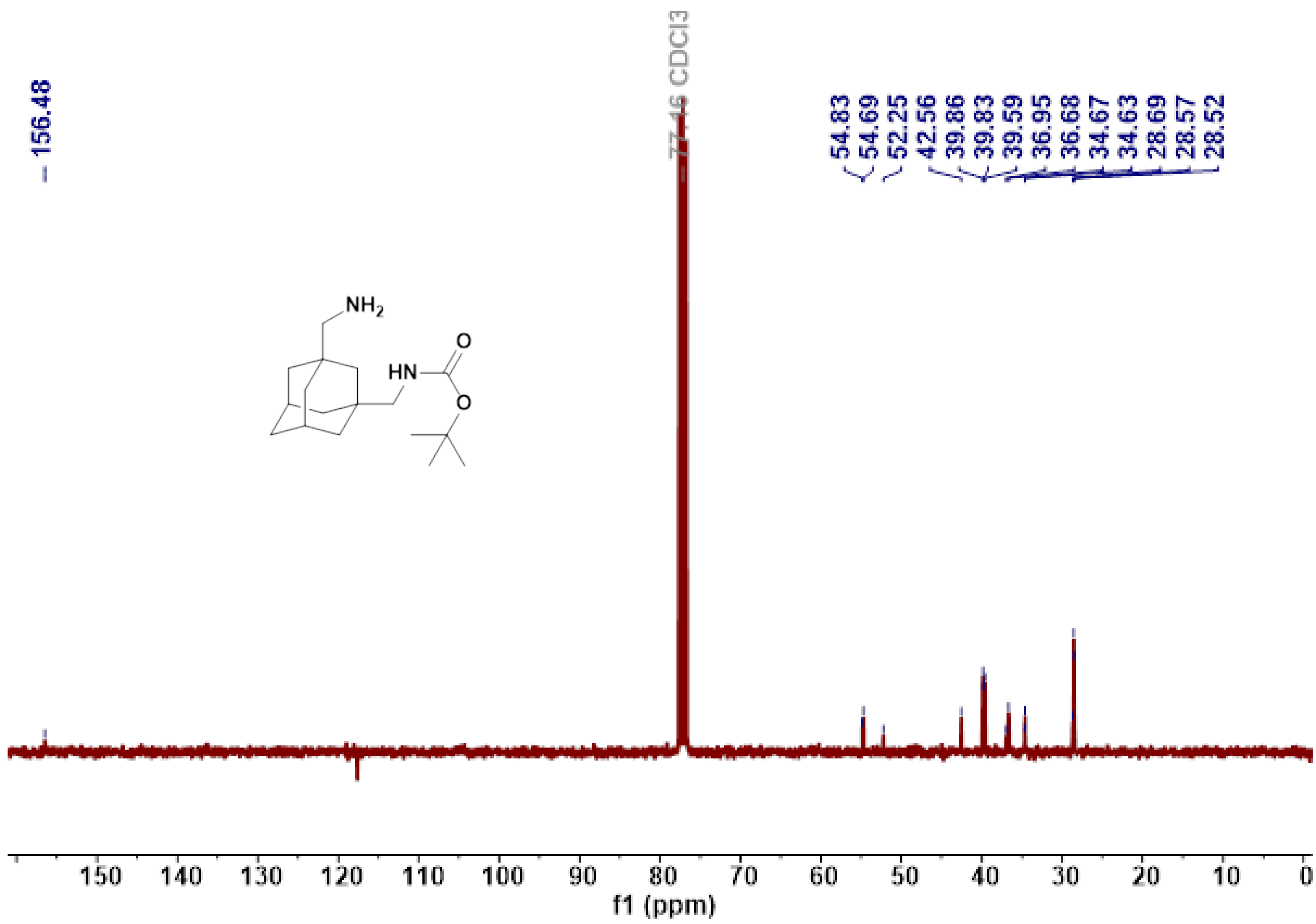

Figure S22. ^{13}C-NMR spectrum of Ada-NHBoc.

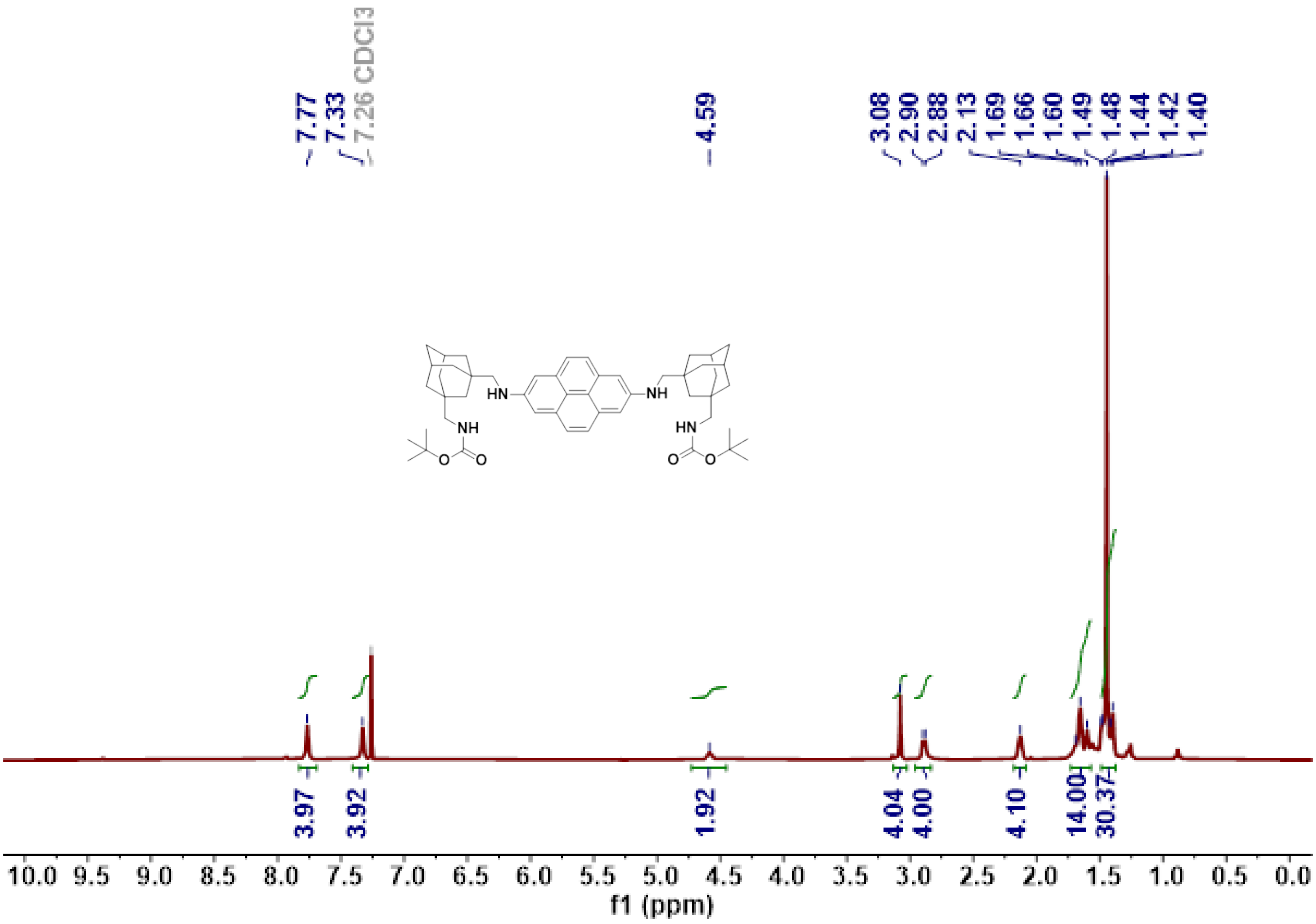

Figure S23. ¹H-NMR spectrum of AdaNHBoc-Py.

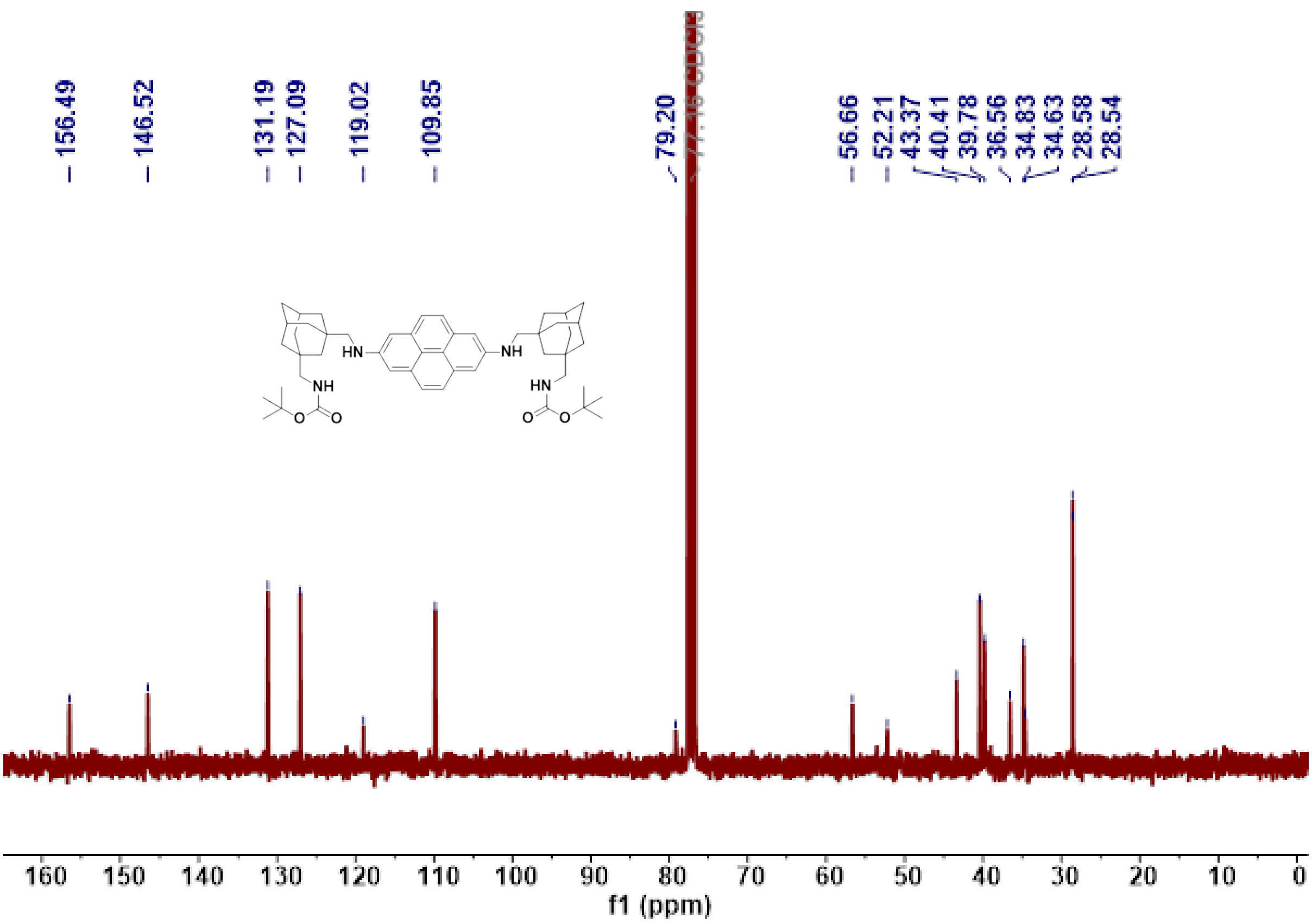

Figure S24. ¹³C-NMR spectrum of AdaNHBoc-Py.

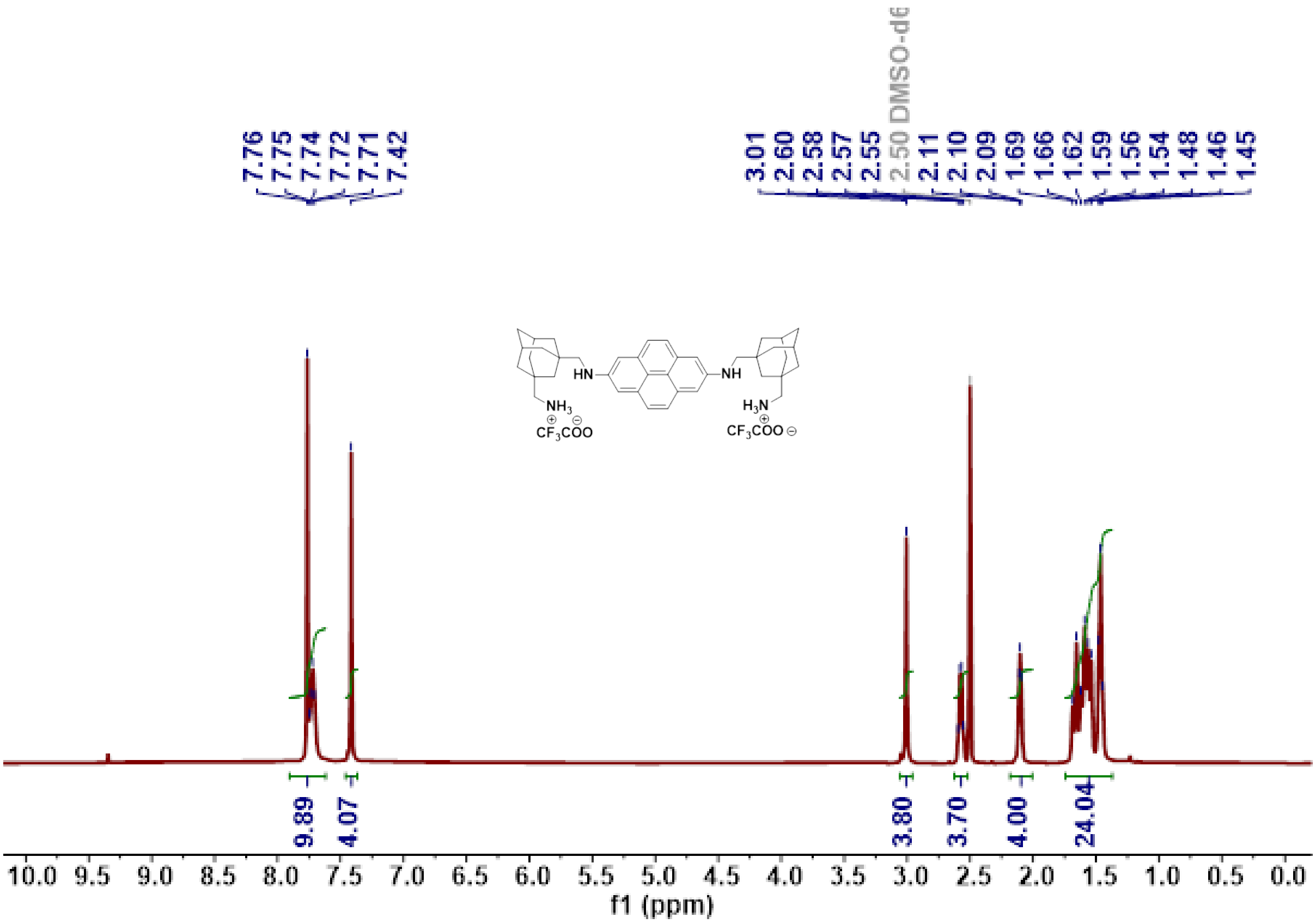

Figure S25. ^{1}H-NMR spectrum of AdaNH-Py.

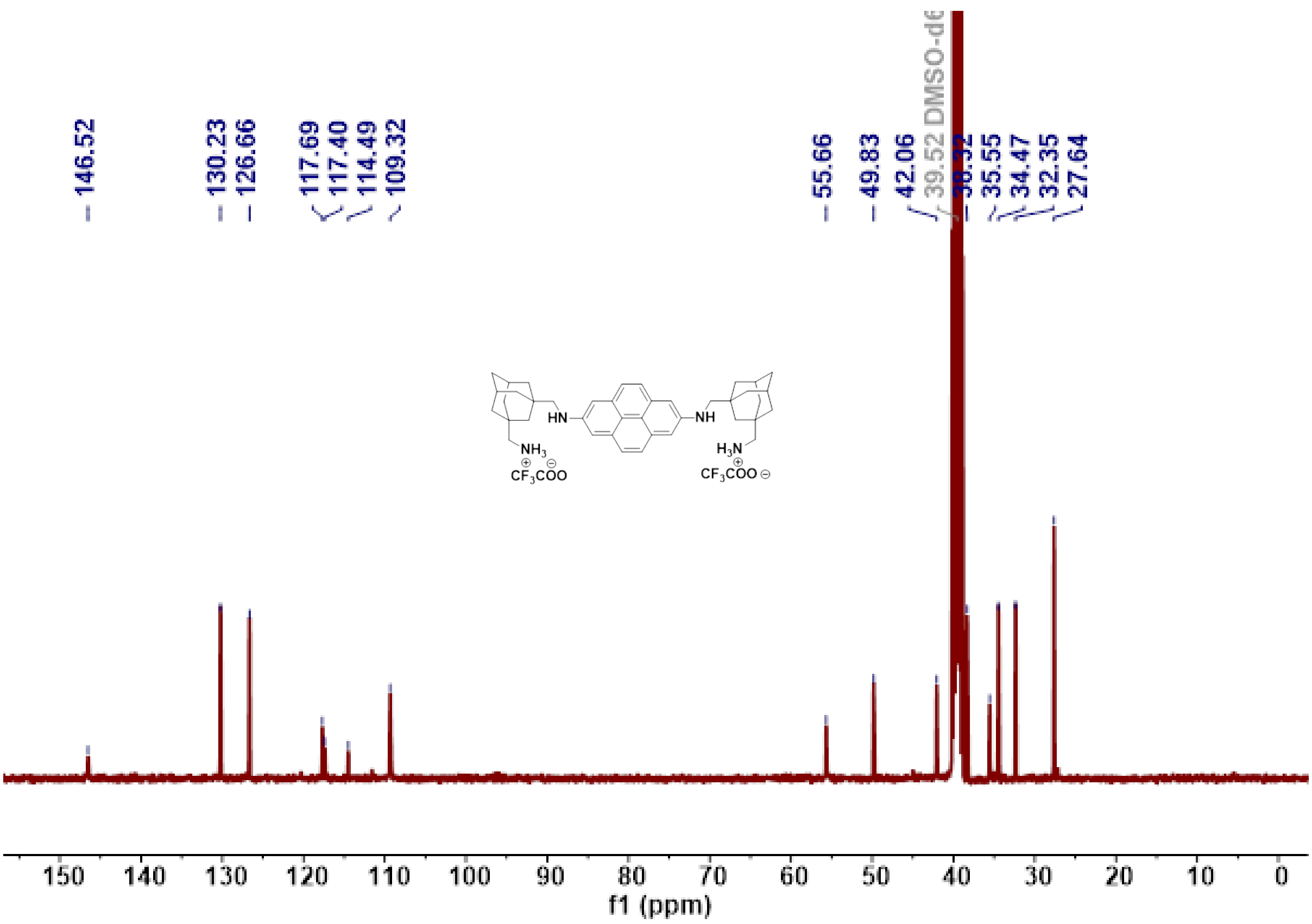

Figure S26. ^{13}C-NMR spectrum of AdaNH-Py.

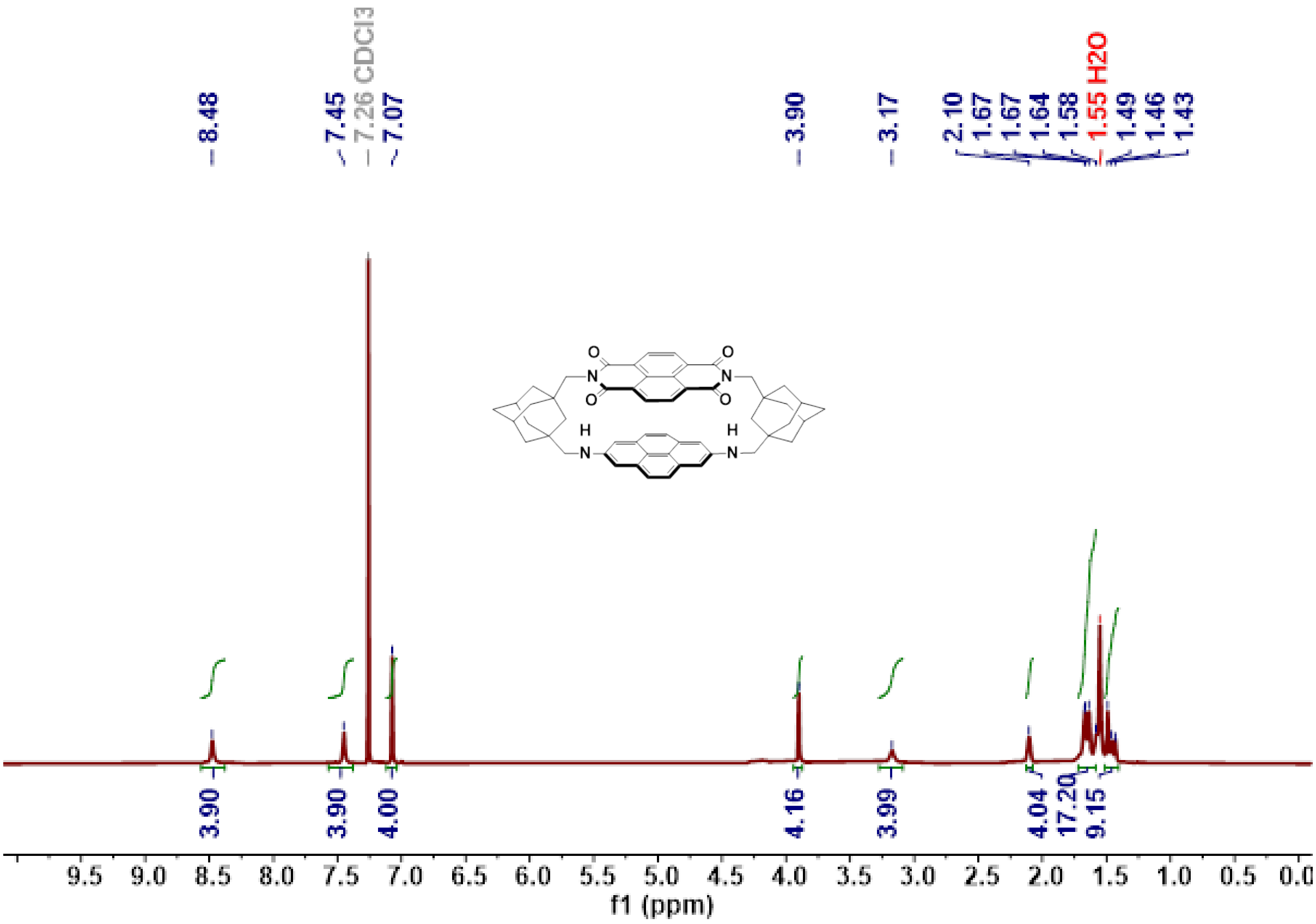

Figure S27. ¹H-NMR spectrum of NDI-AdaNH-Py.

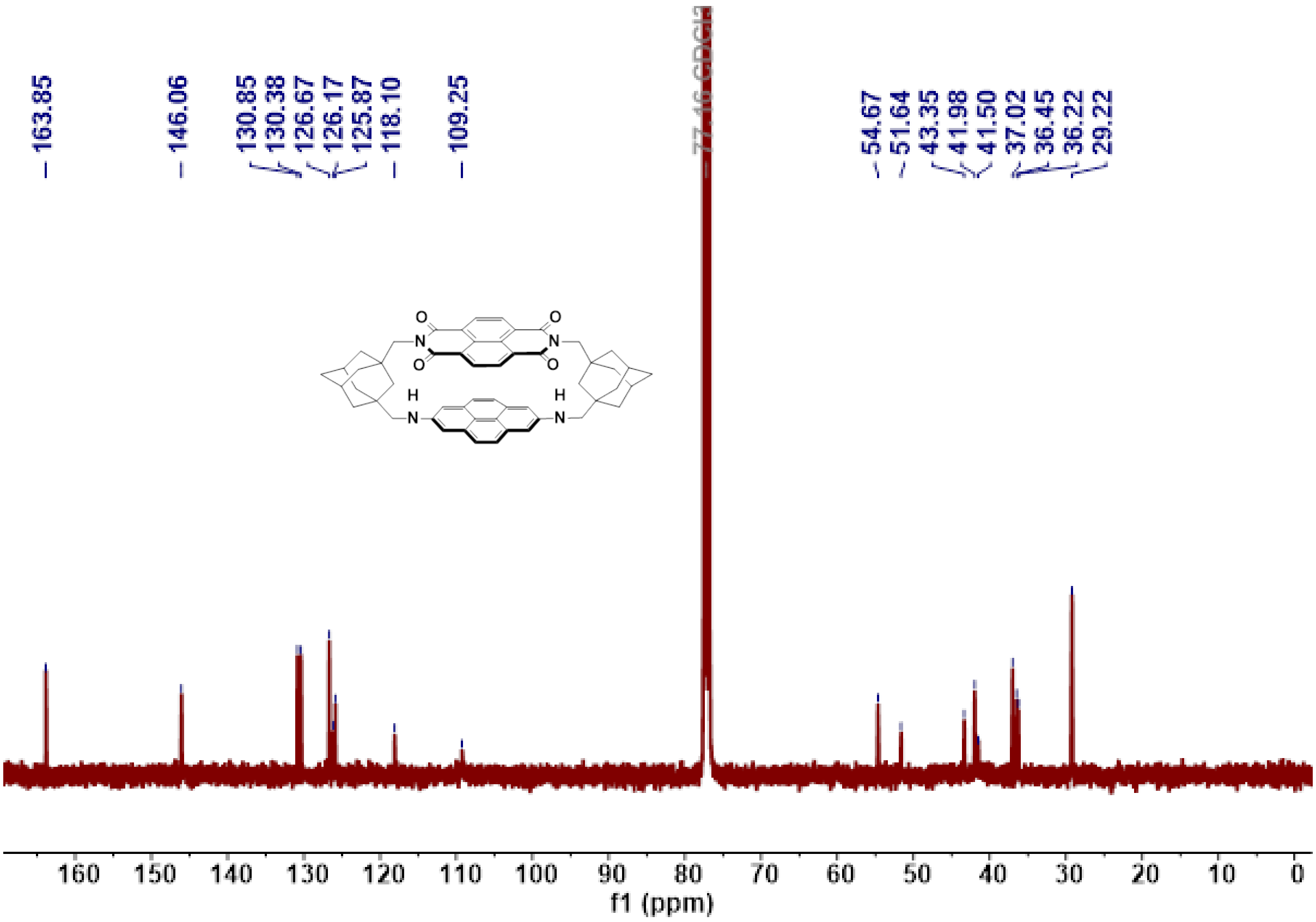

Figure S28. ¹³C-NMR spectrum of NDI-AdaNH-Py.

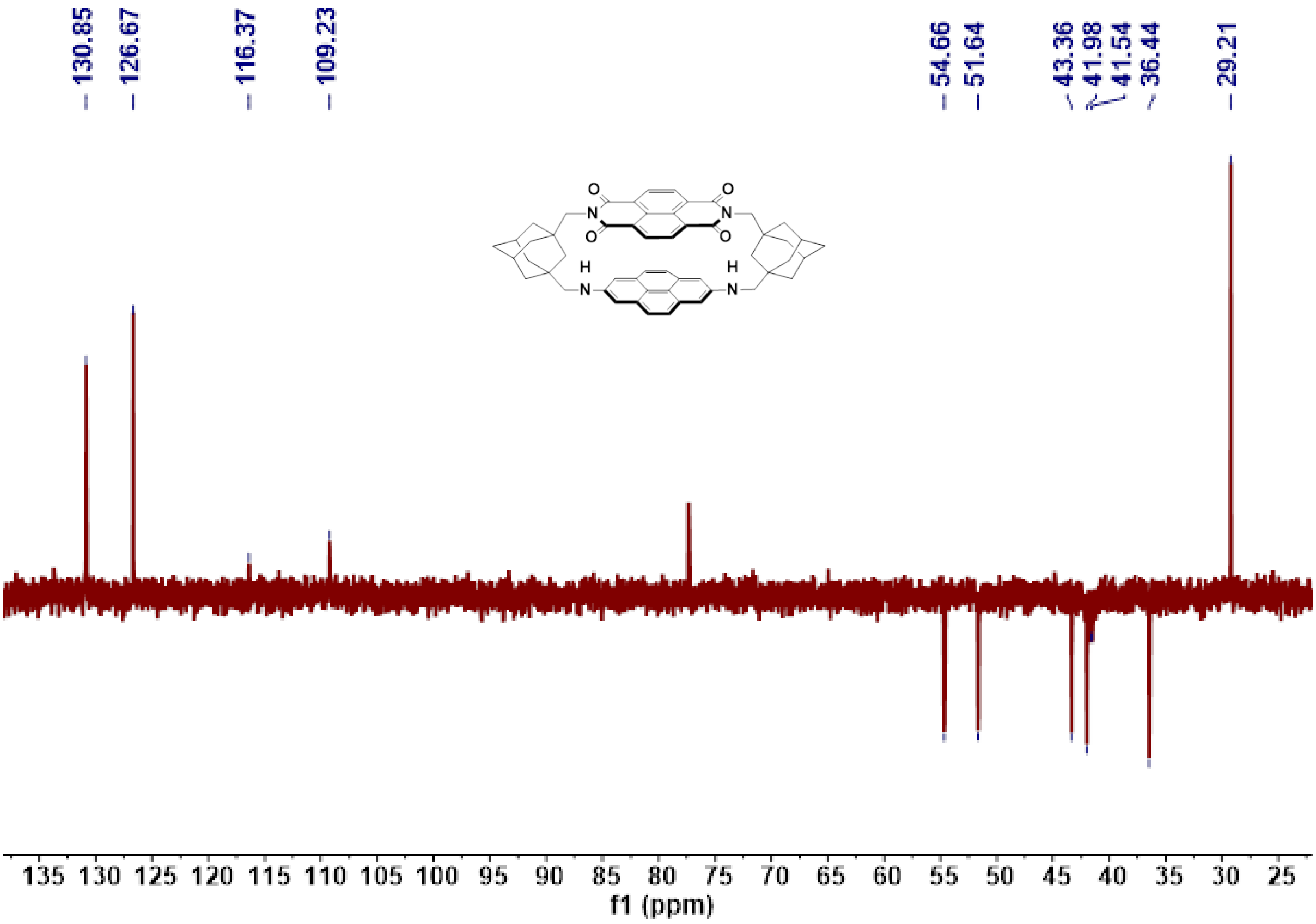

Figure S29. DEPT-135 spectrum of NDI-AdaNH-Py.

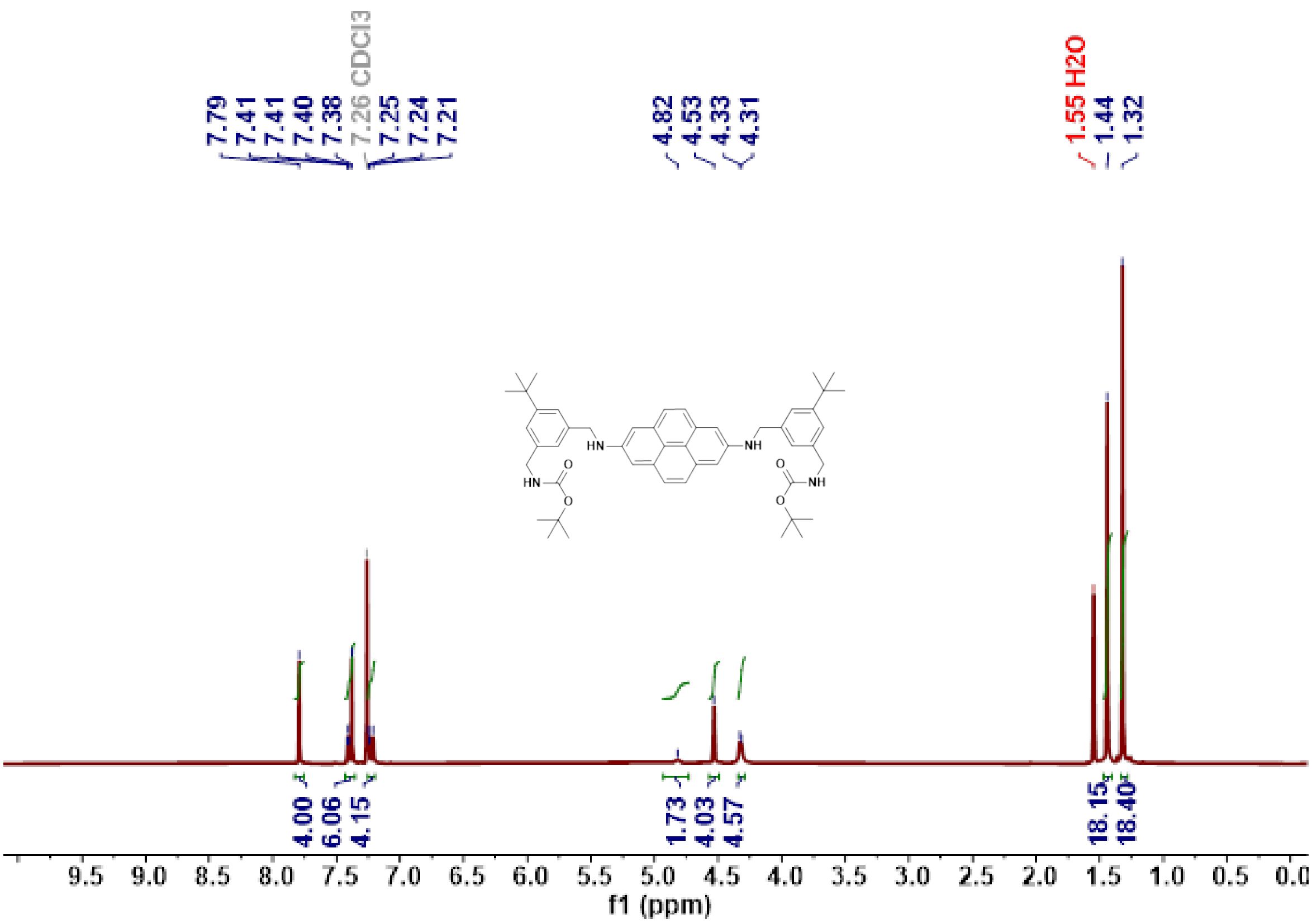

Figure S30. ^{1}H-NMR spectrum of BnNHBoc-Py.

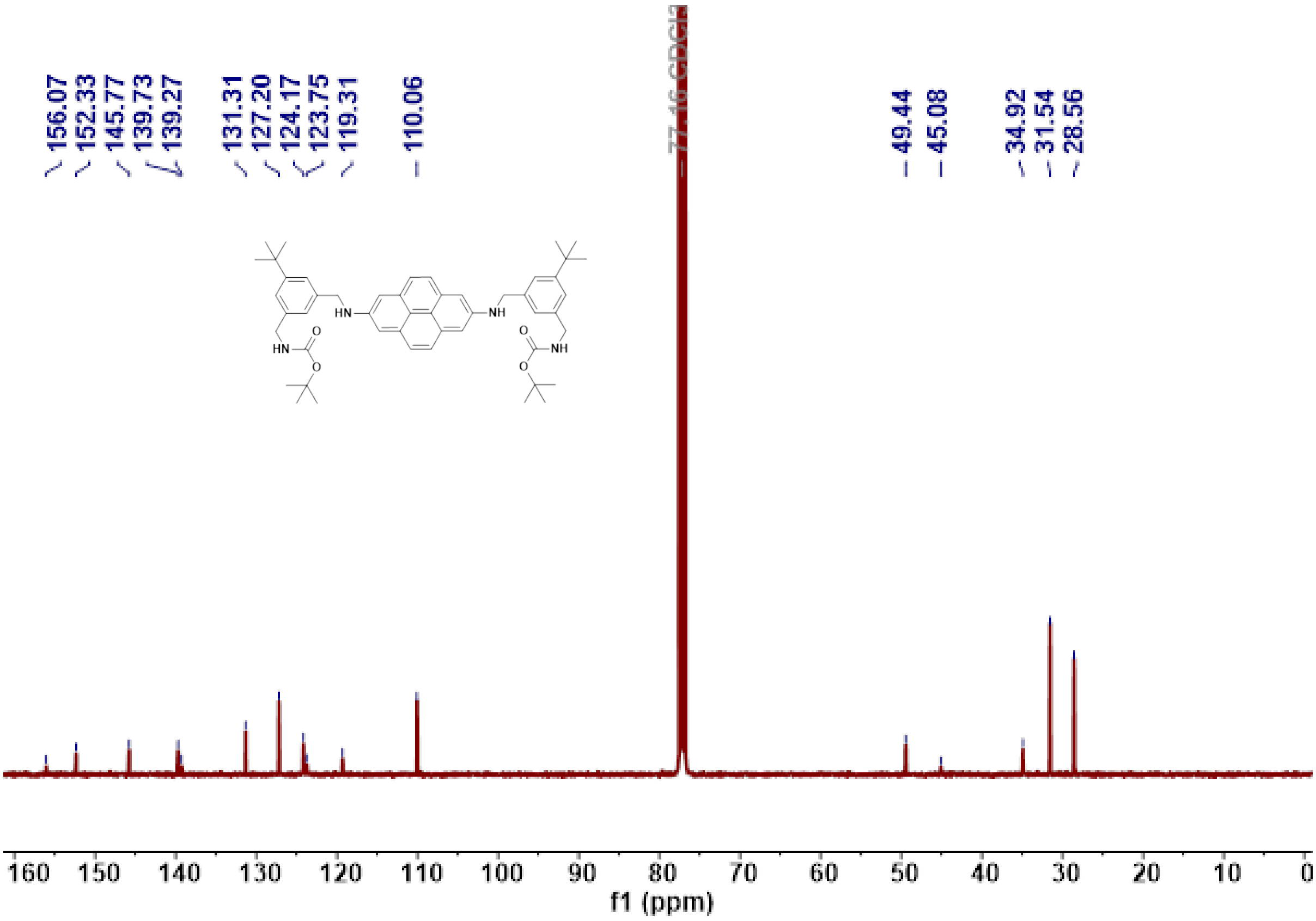

Figure S31. ^{13}C-NMR spectrum of BnNHBoc-Py.

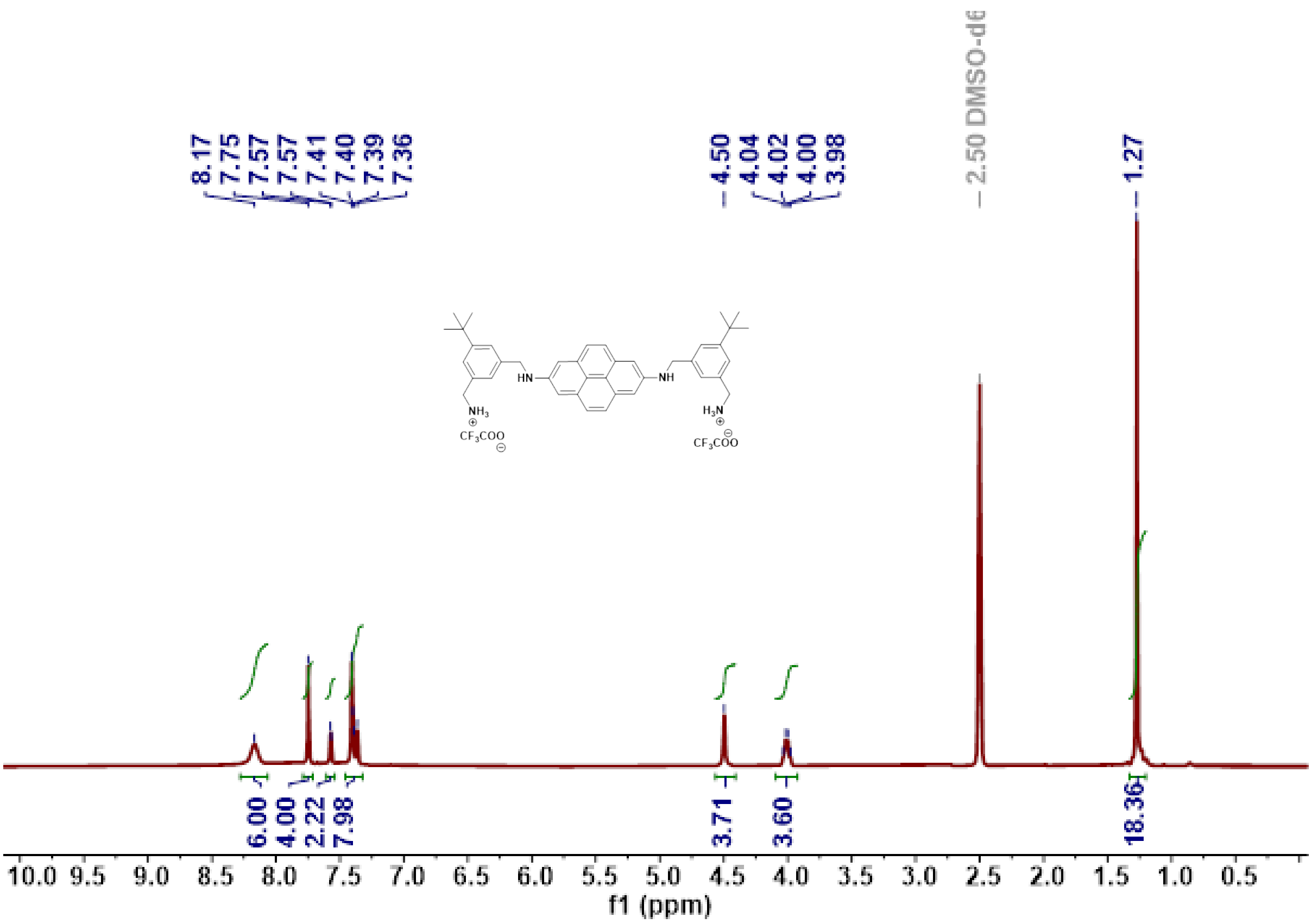

Figure S32. ^{1}H-NMR spectrum of BnNH-Py.

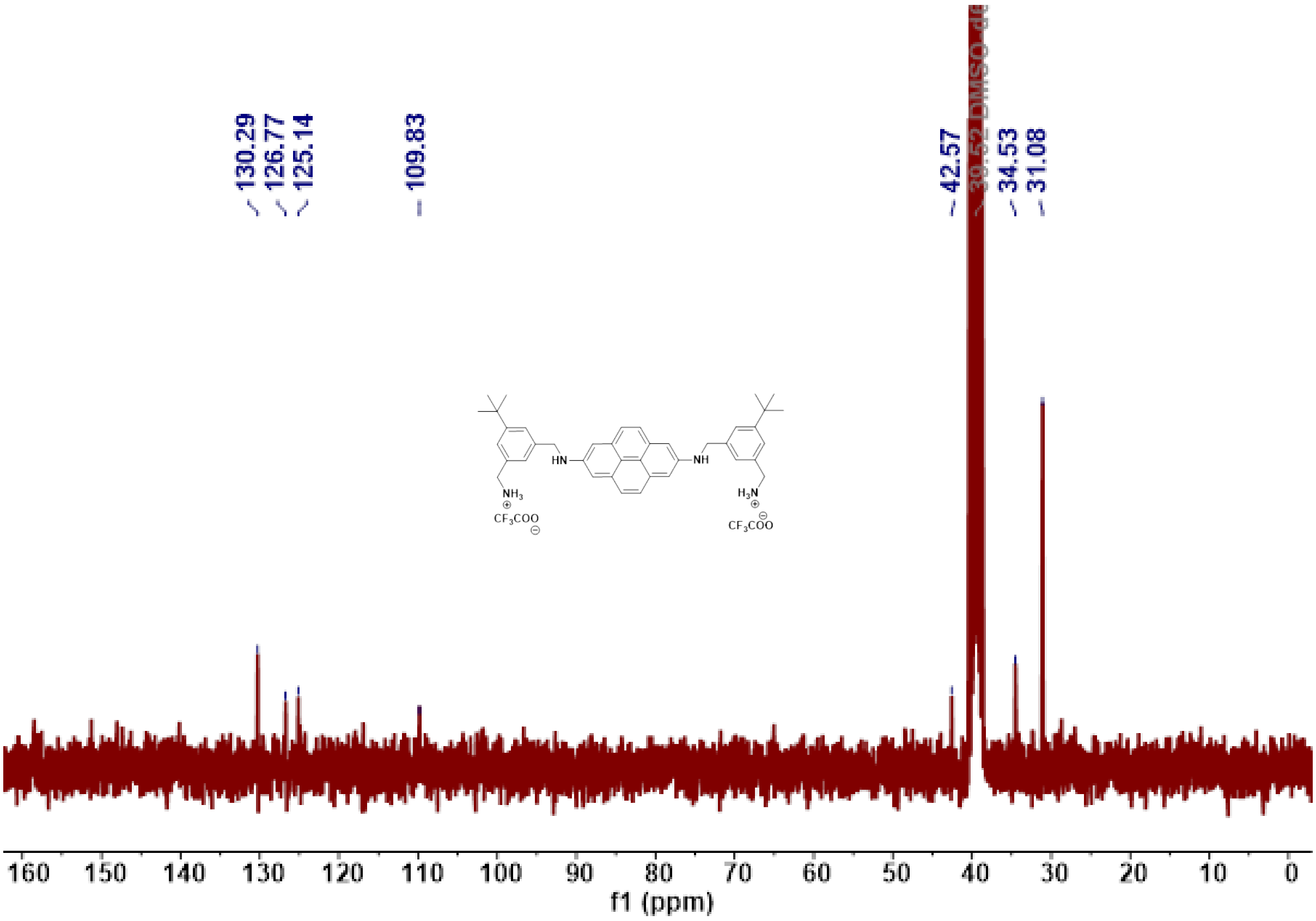

Figure S33. ^{13}C-NMR spectrum of BnNH-Py.

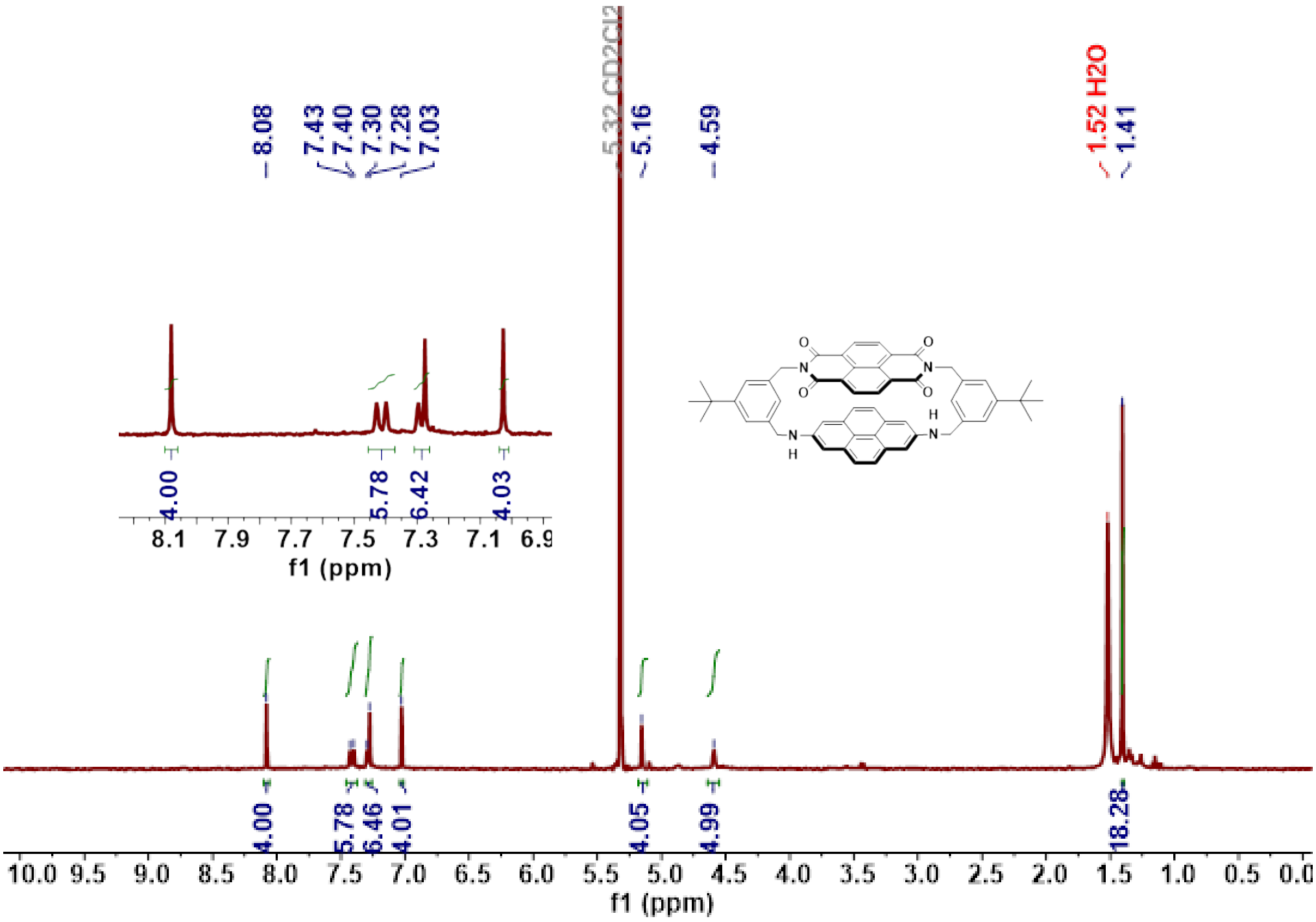

Figure S34. ^{1}H-NMR spectrum of NDI-BnNH-Py.

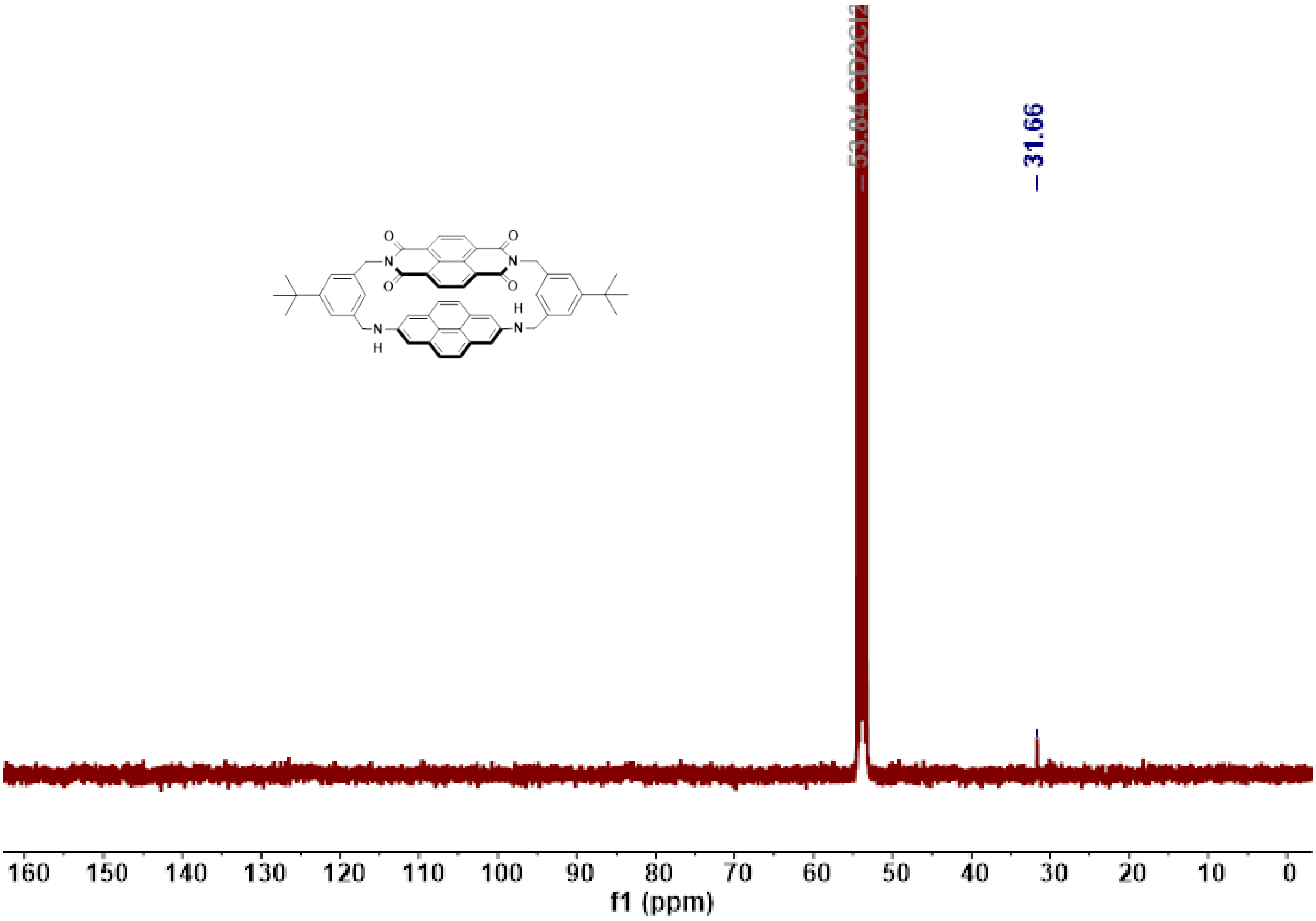

Figure S35. ^{13}C-NMR spectrum of NDI-BnNH-Py.

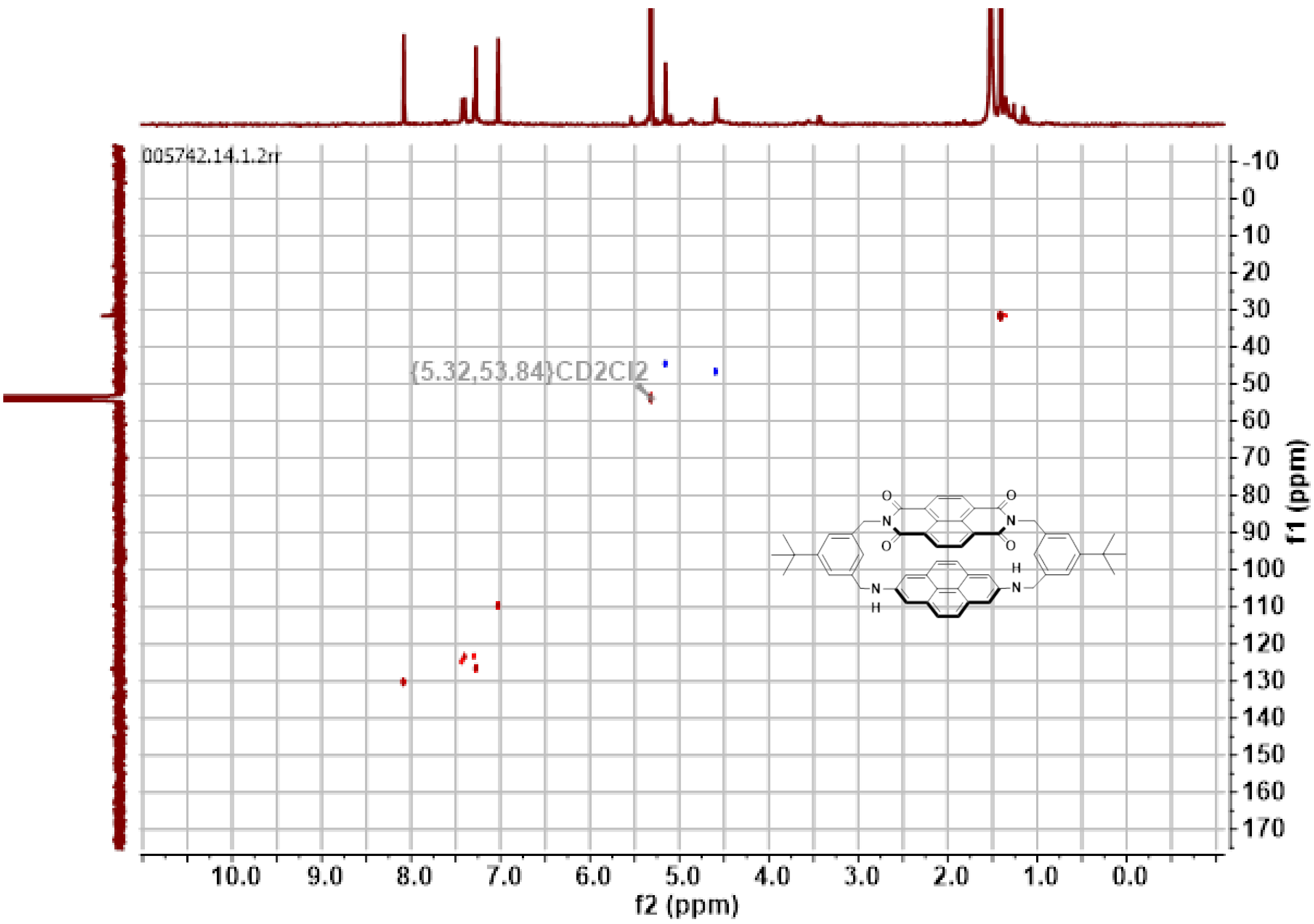

Figure S36. HSQC spectrum of NDI-BnNH-Py.

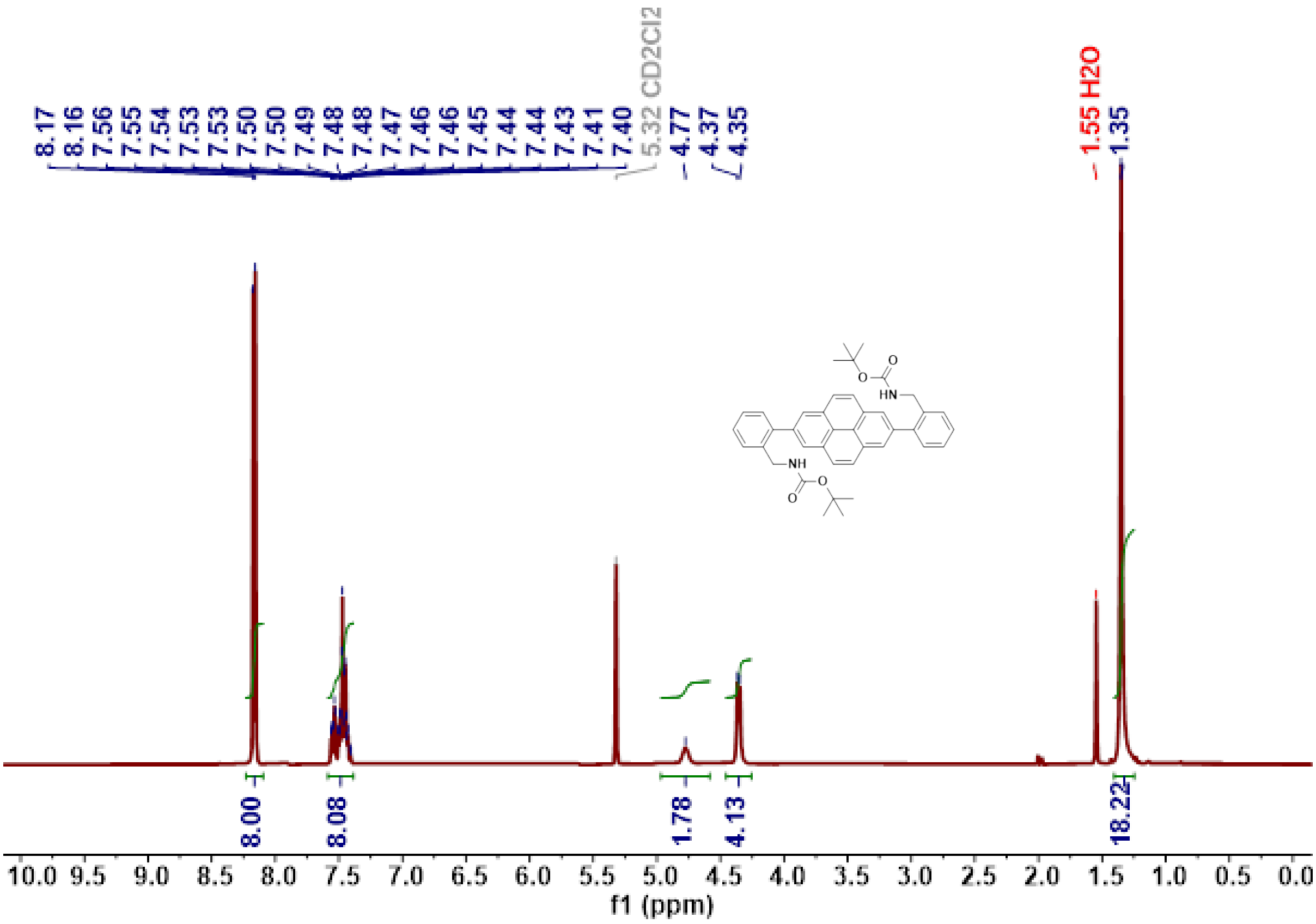

Figure S37. ^{1}H-NMR spectrum of PhNHBoc-Py.

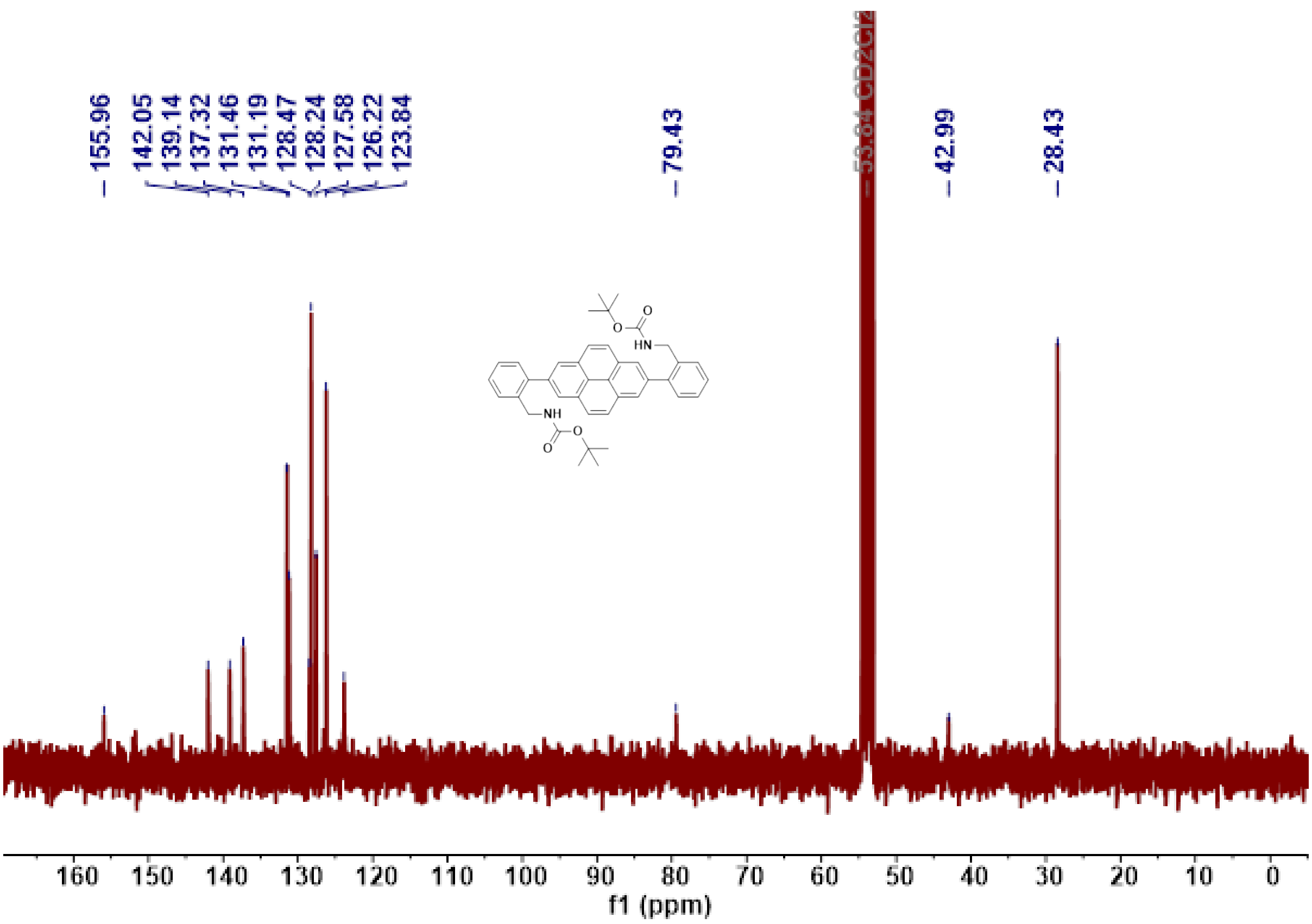

Figure S38. ^{13}C-NMR spectrum of PhNHBoc-Py.

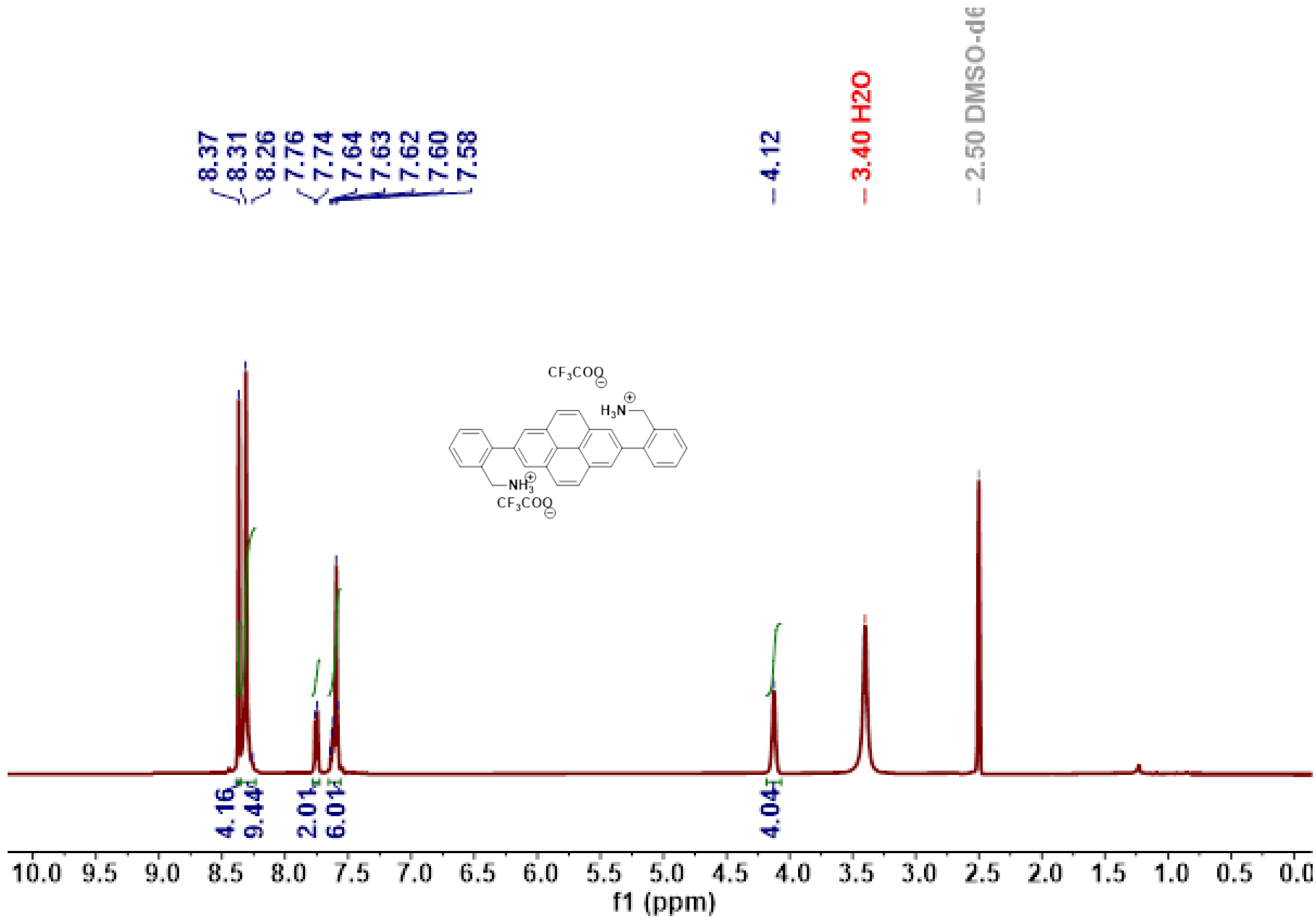

Figure S39. ^{1}H-NMR spectrum of PhNH-Py.

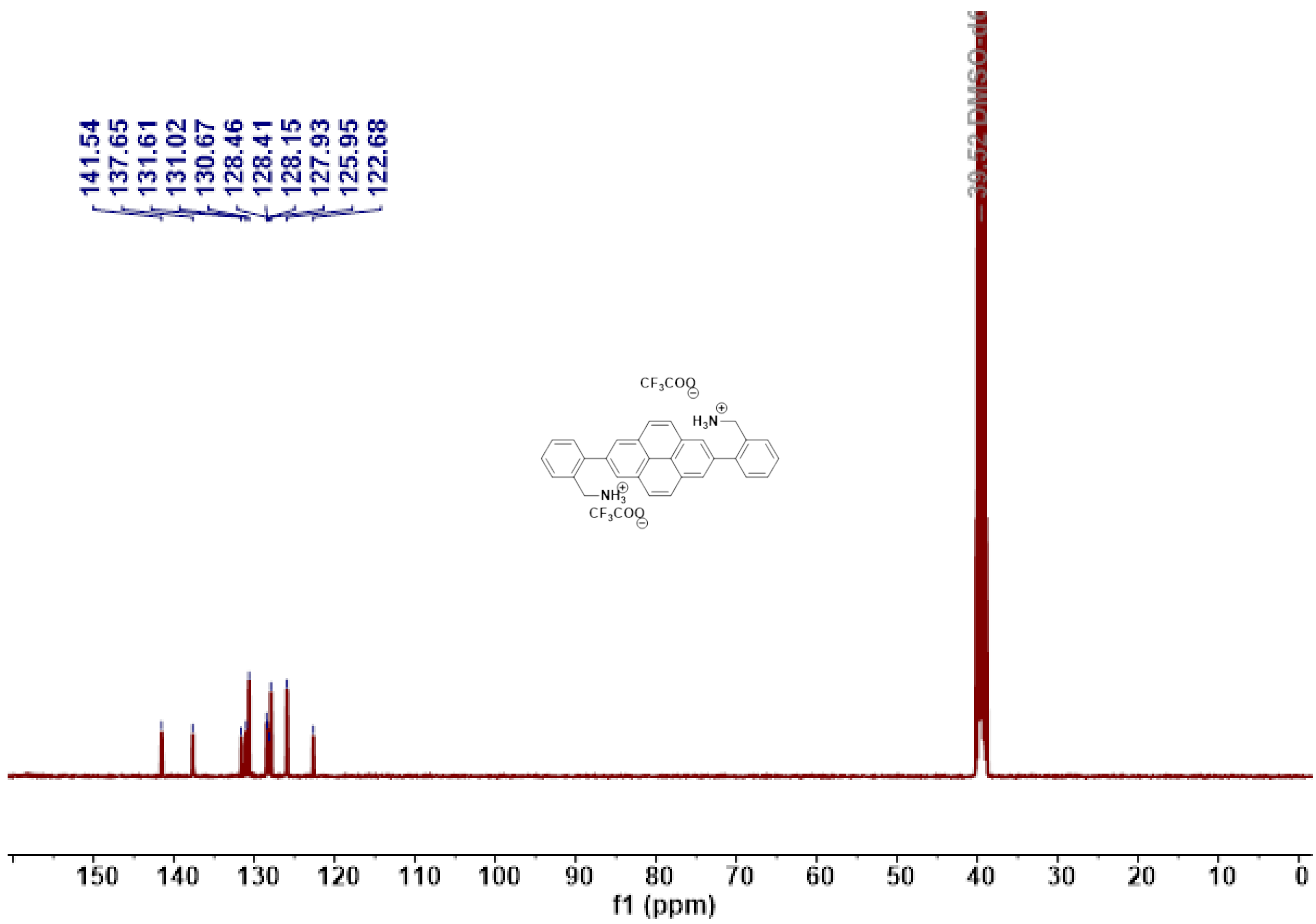

Figure S40. ^{13}C-NMR spectrum of PhNH-Py.

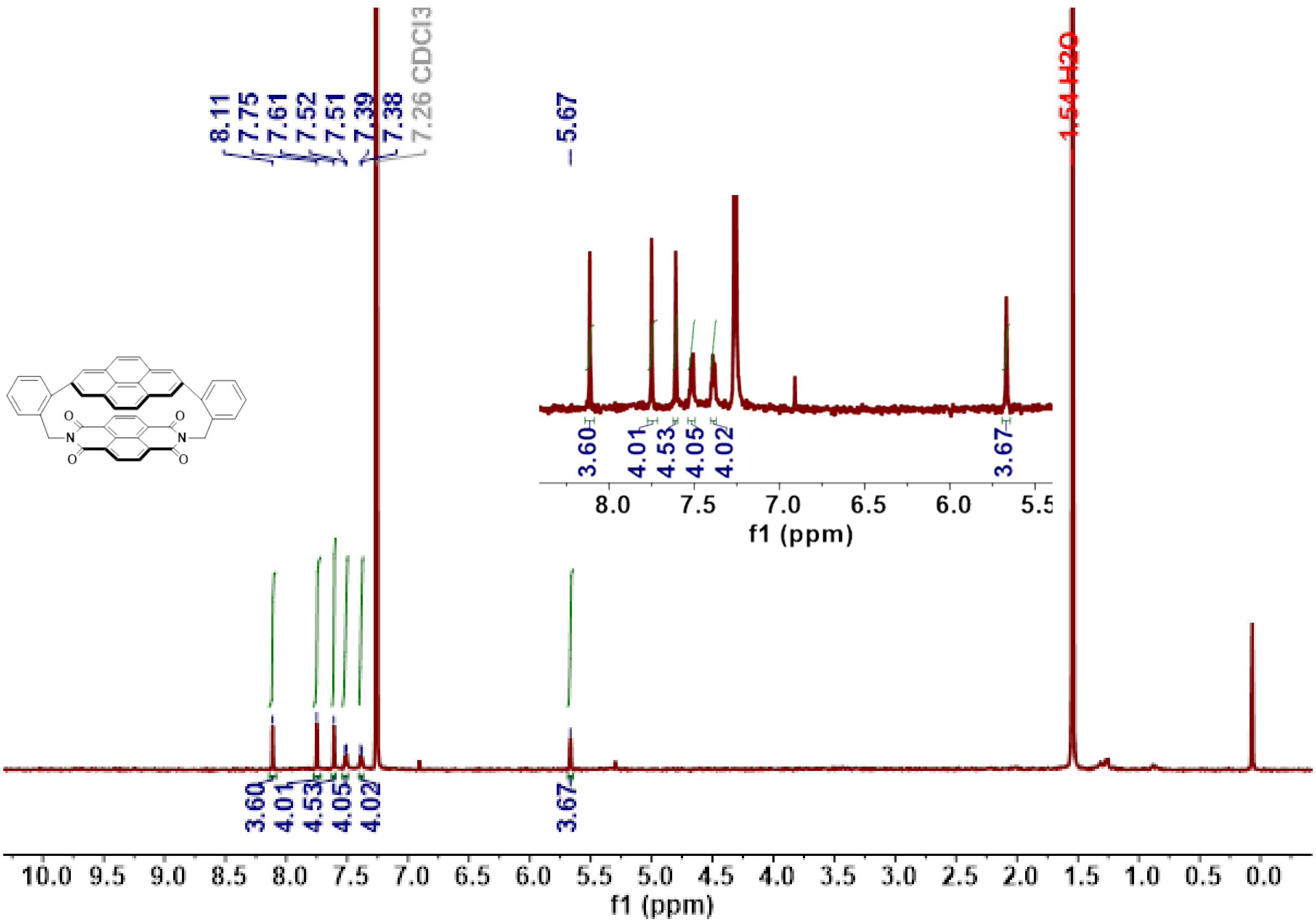

Figure S41. ¹H-NMR spectrum of NDI-Ph-Py.

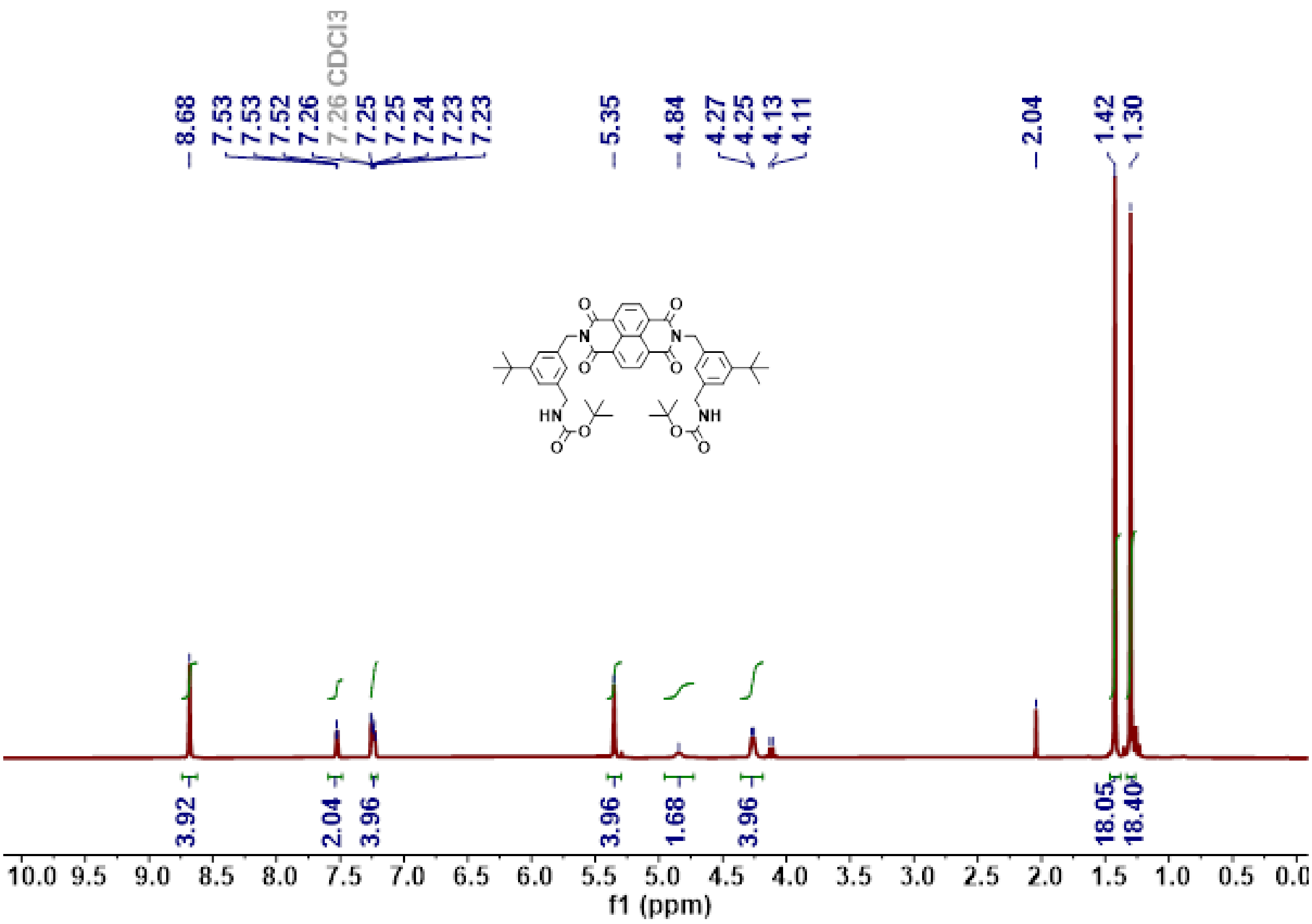

Figure S42. ¹H-NMR spectrum of the reference compound NDI.

Mass spectra

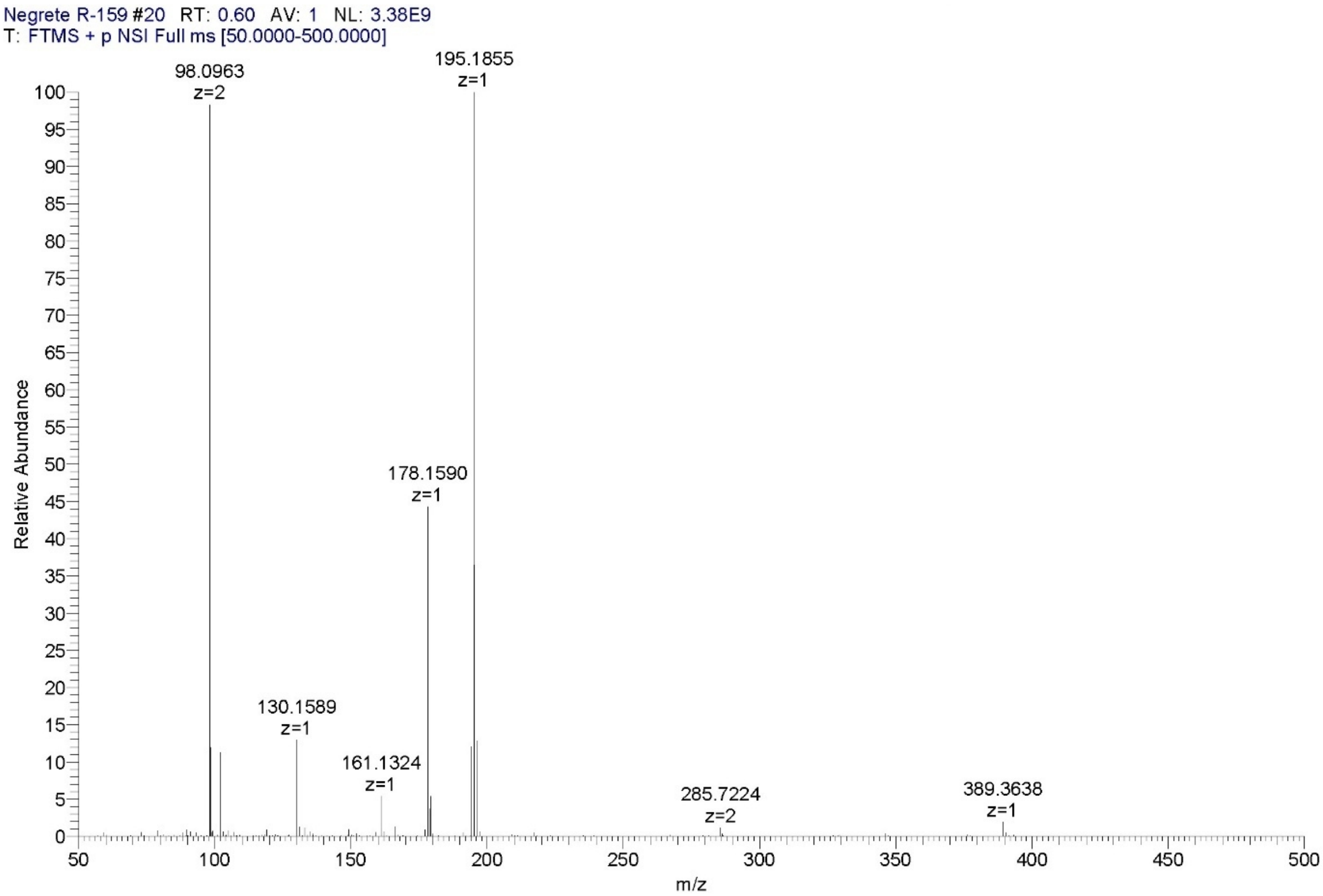

Mass spectrum of Ada-NH_2.

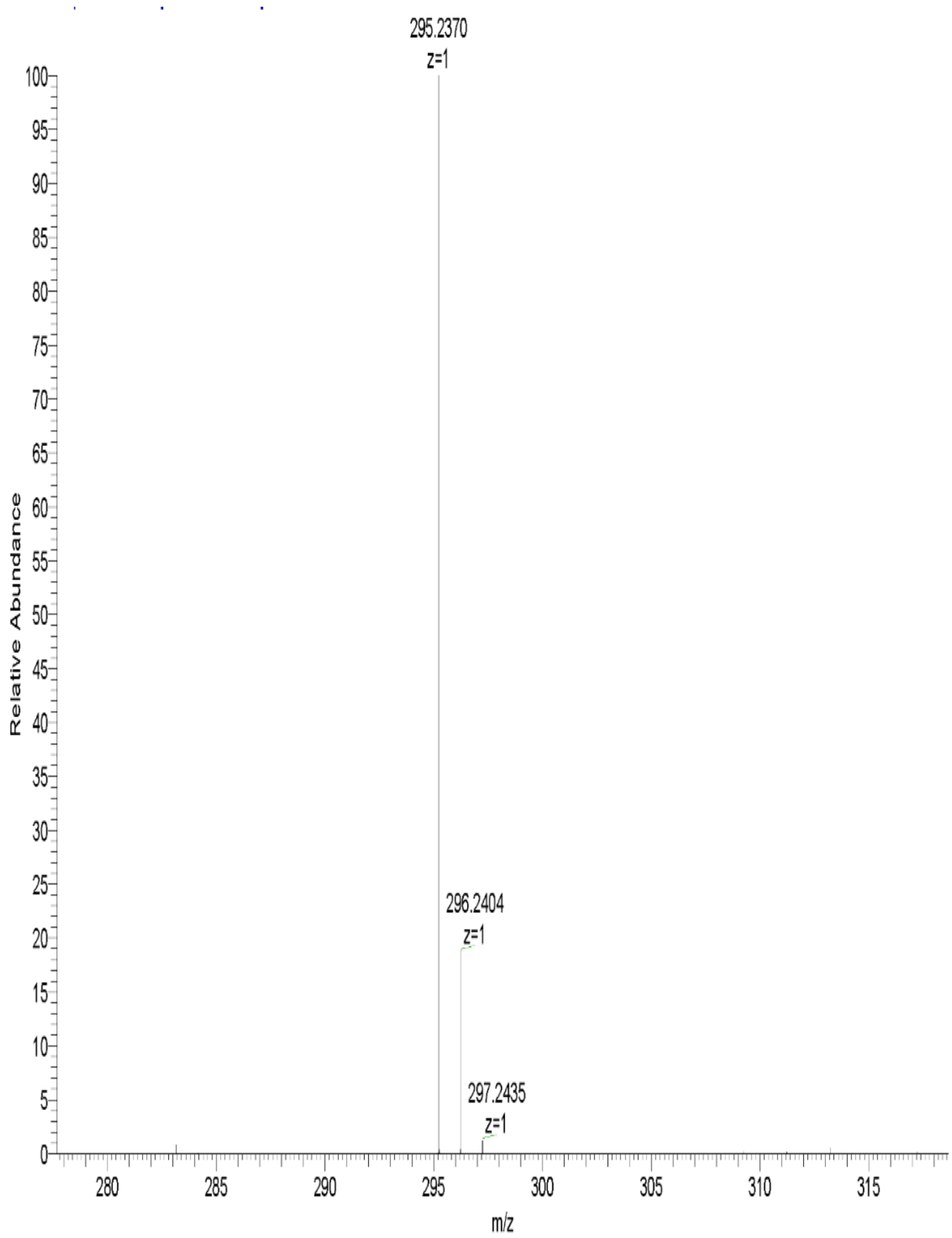

Mass spectrum of Ada-NHBoc.

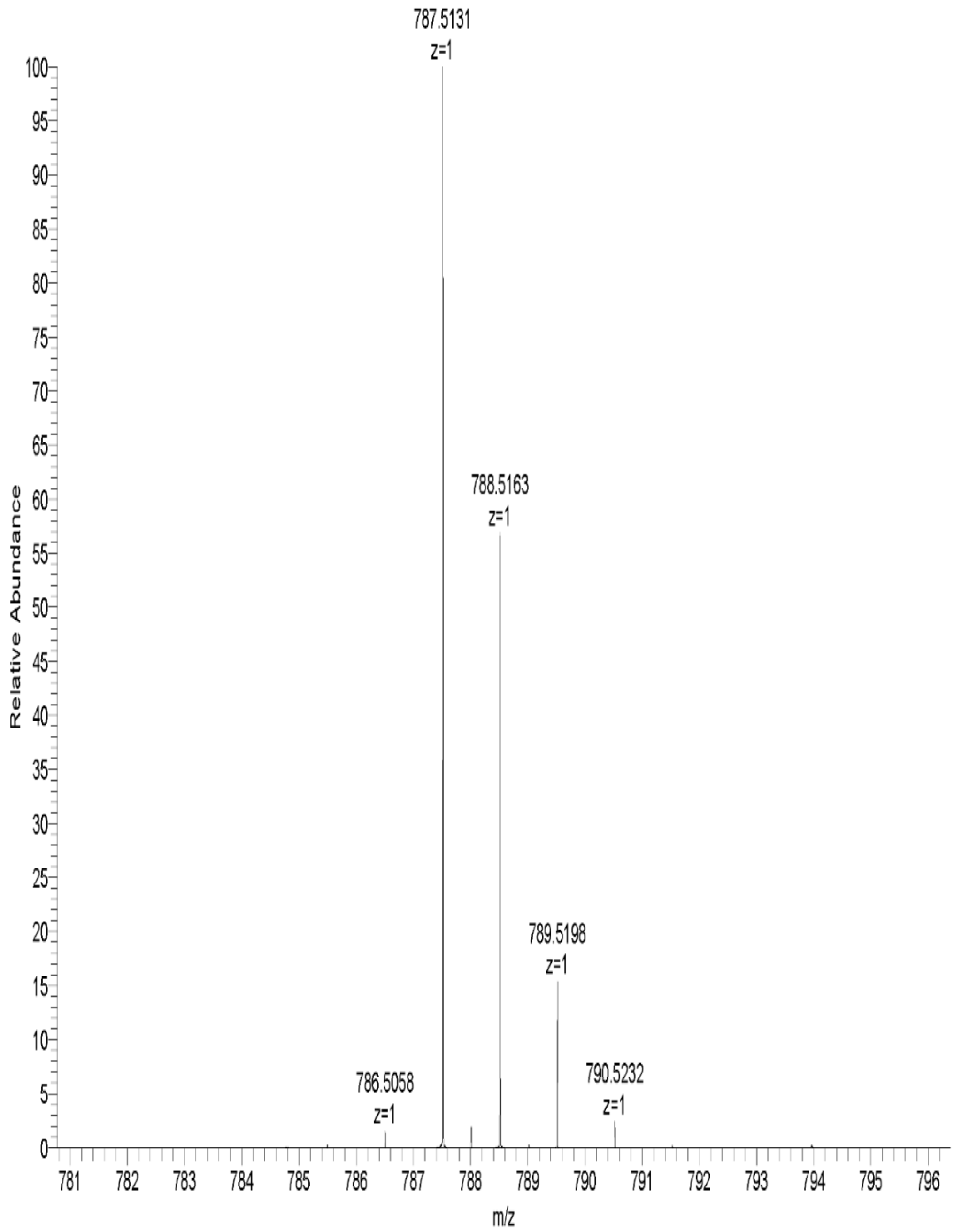

Mass spectrum of AdaNHBoc-Py.

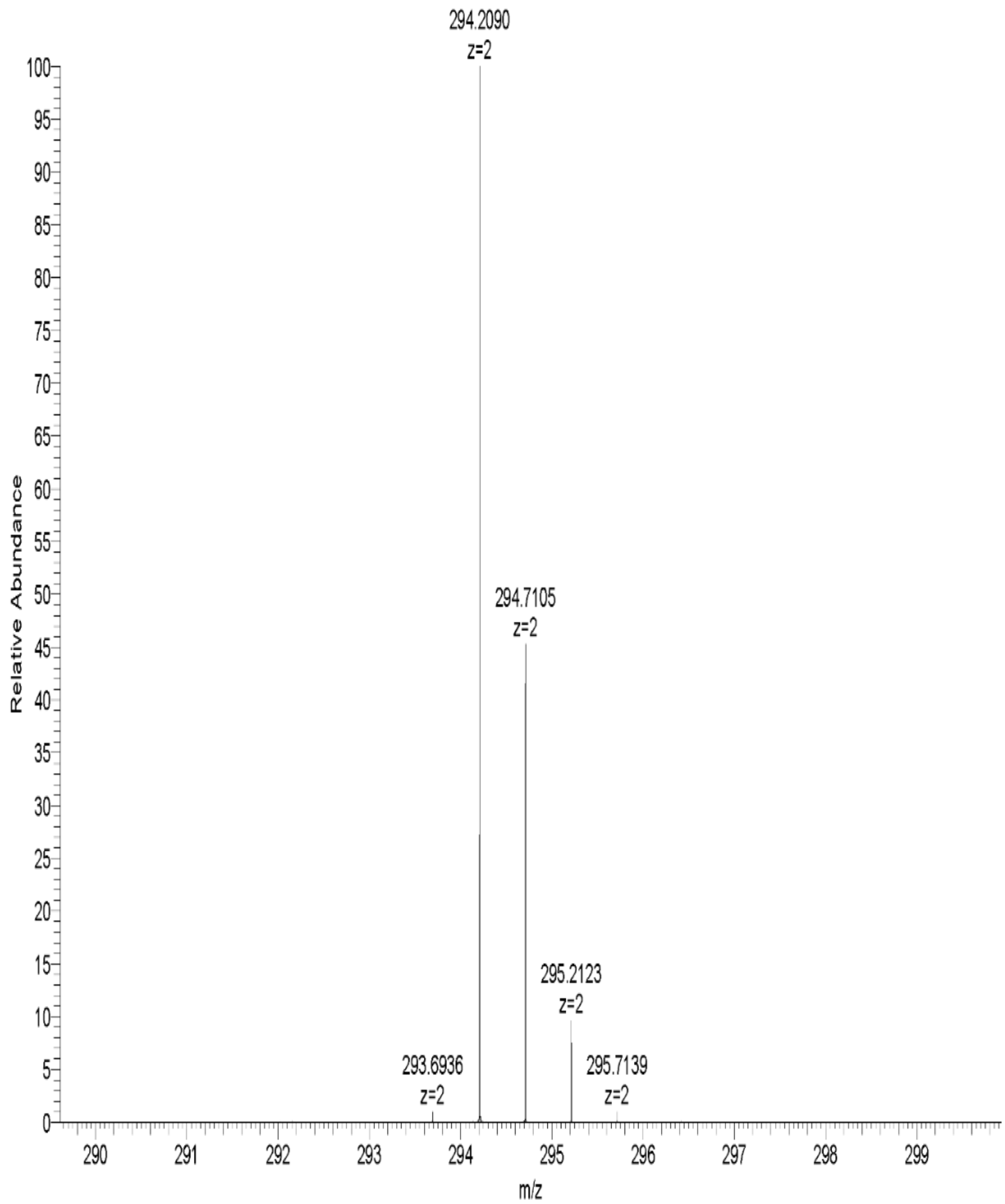

Mass spectrum of AdaNH-Py.

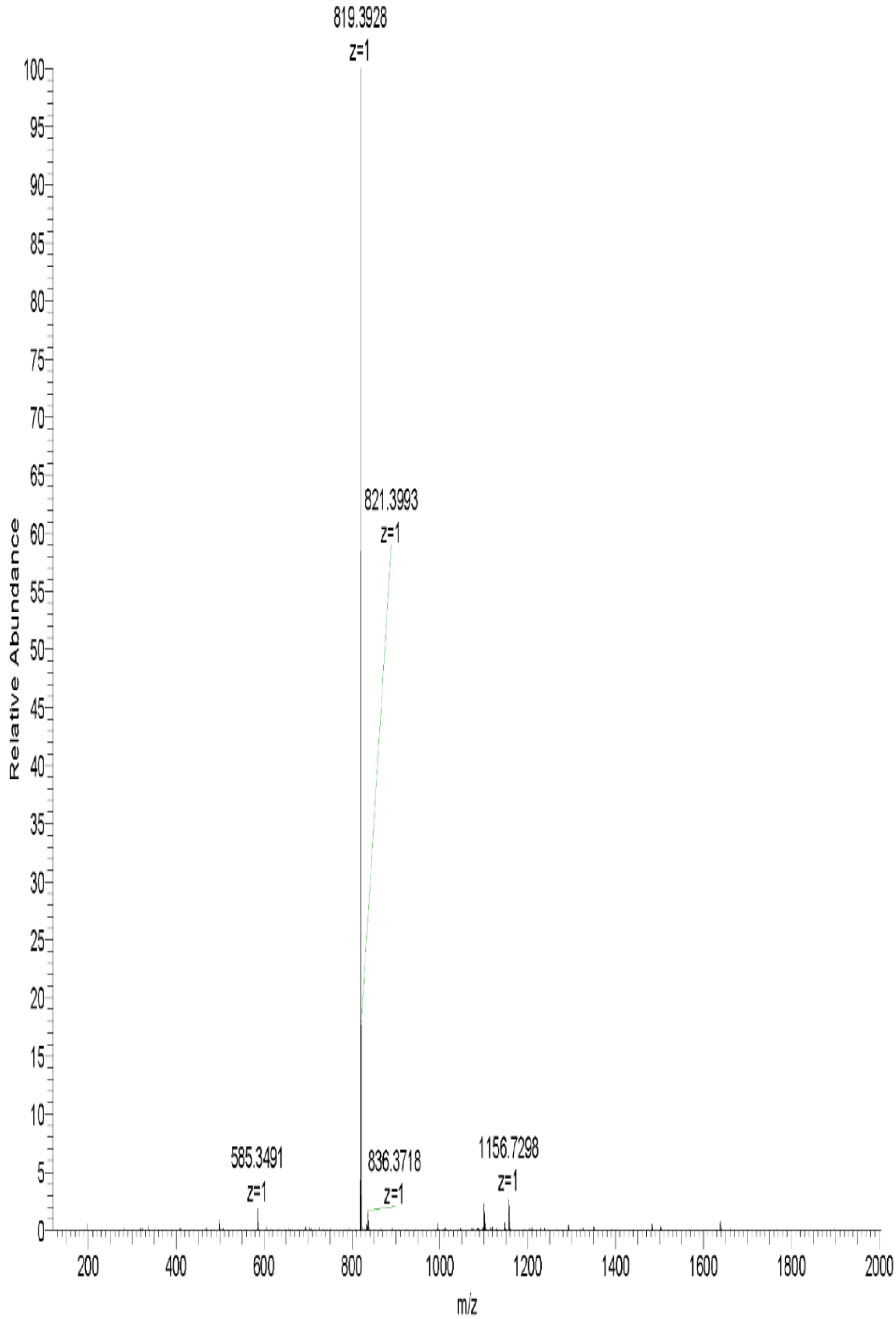

Mass spectrum of NDI-AdaNH-Py.

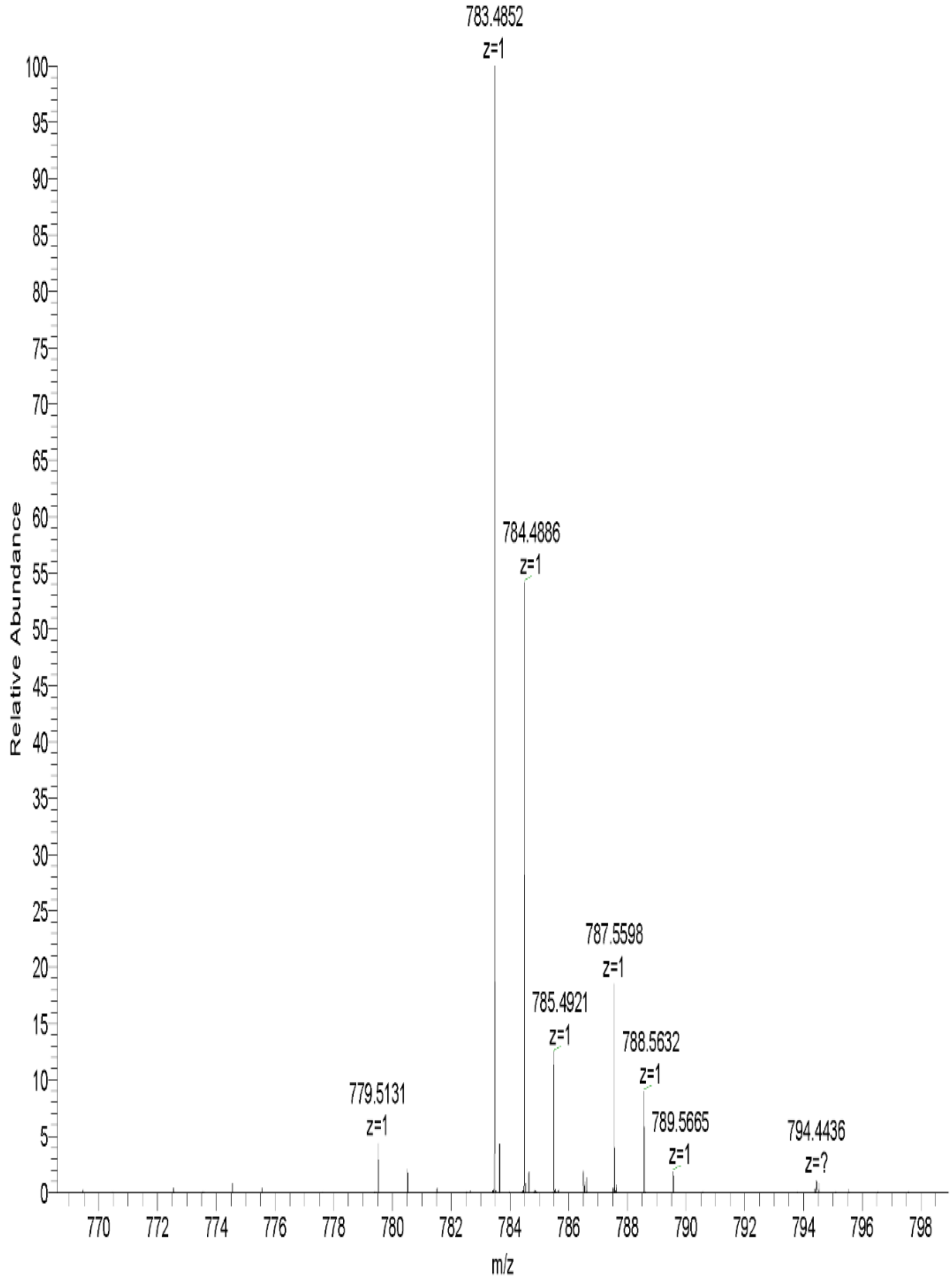

Mass spectrum of BnNHBoc-Py.

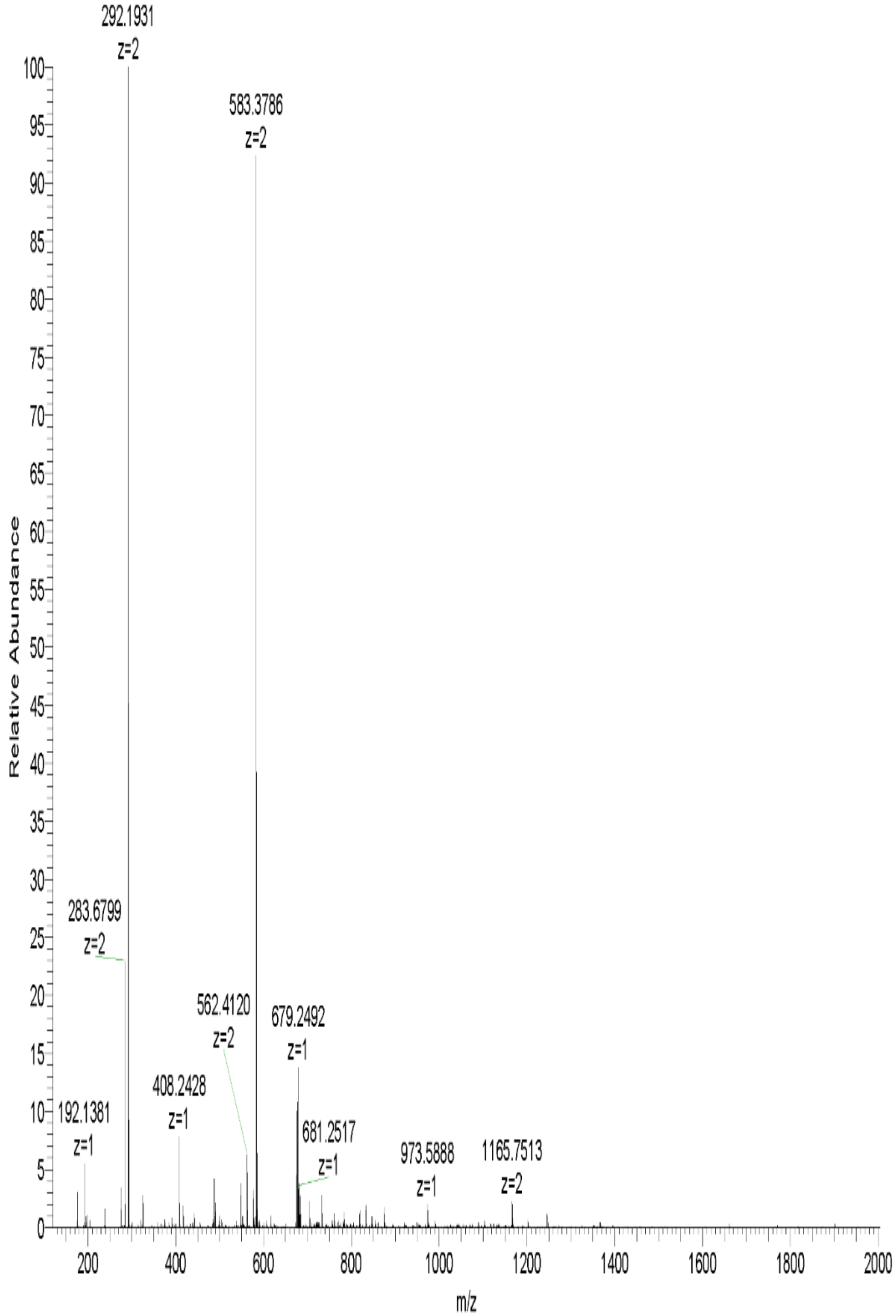

Mass spectrum of BnNH-Py.

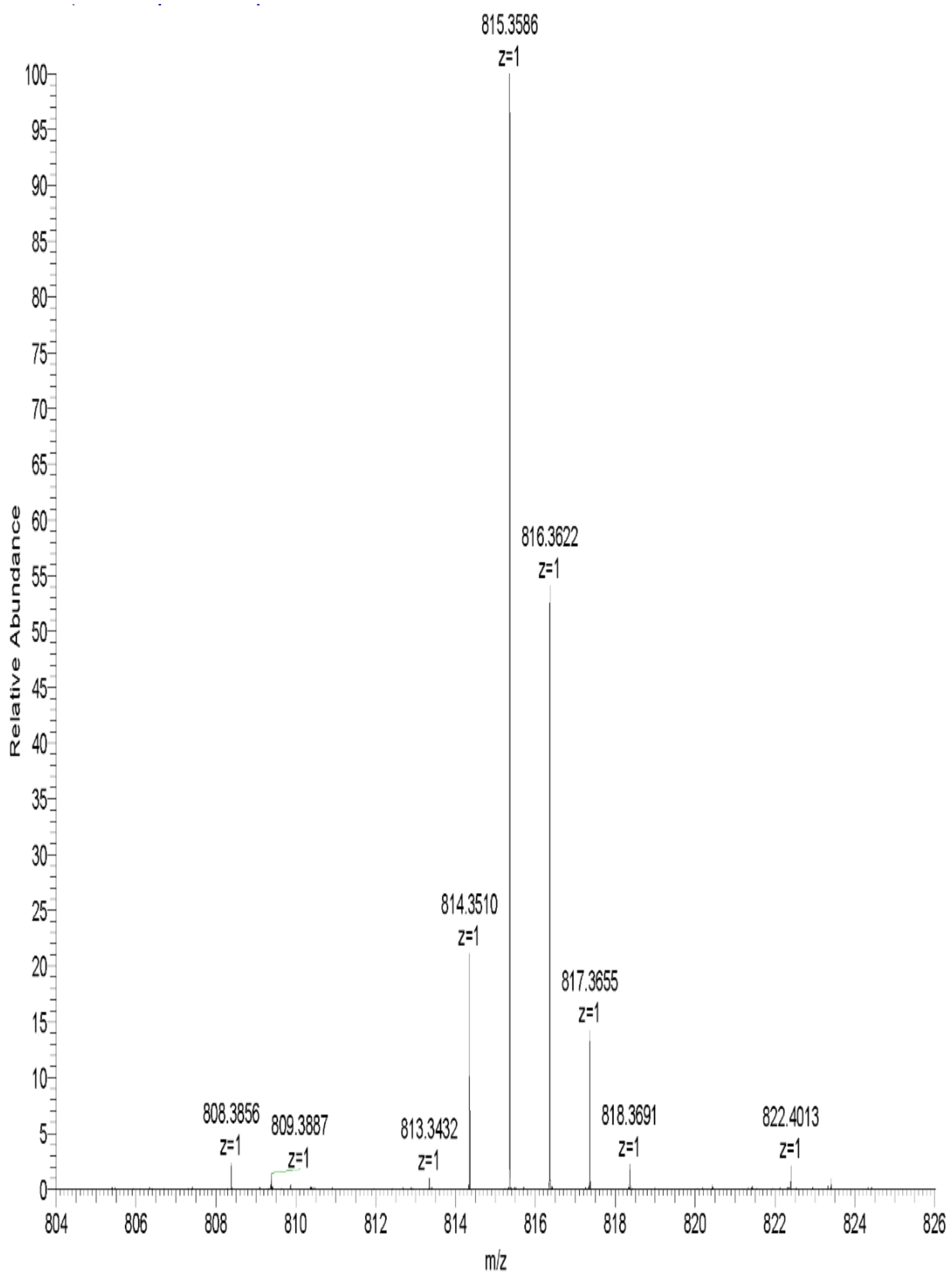

Mass spectrum of NDI-BnNH-Py.

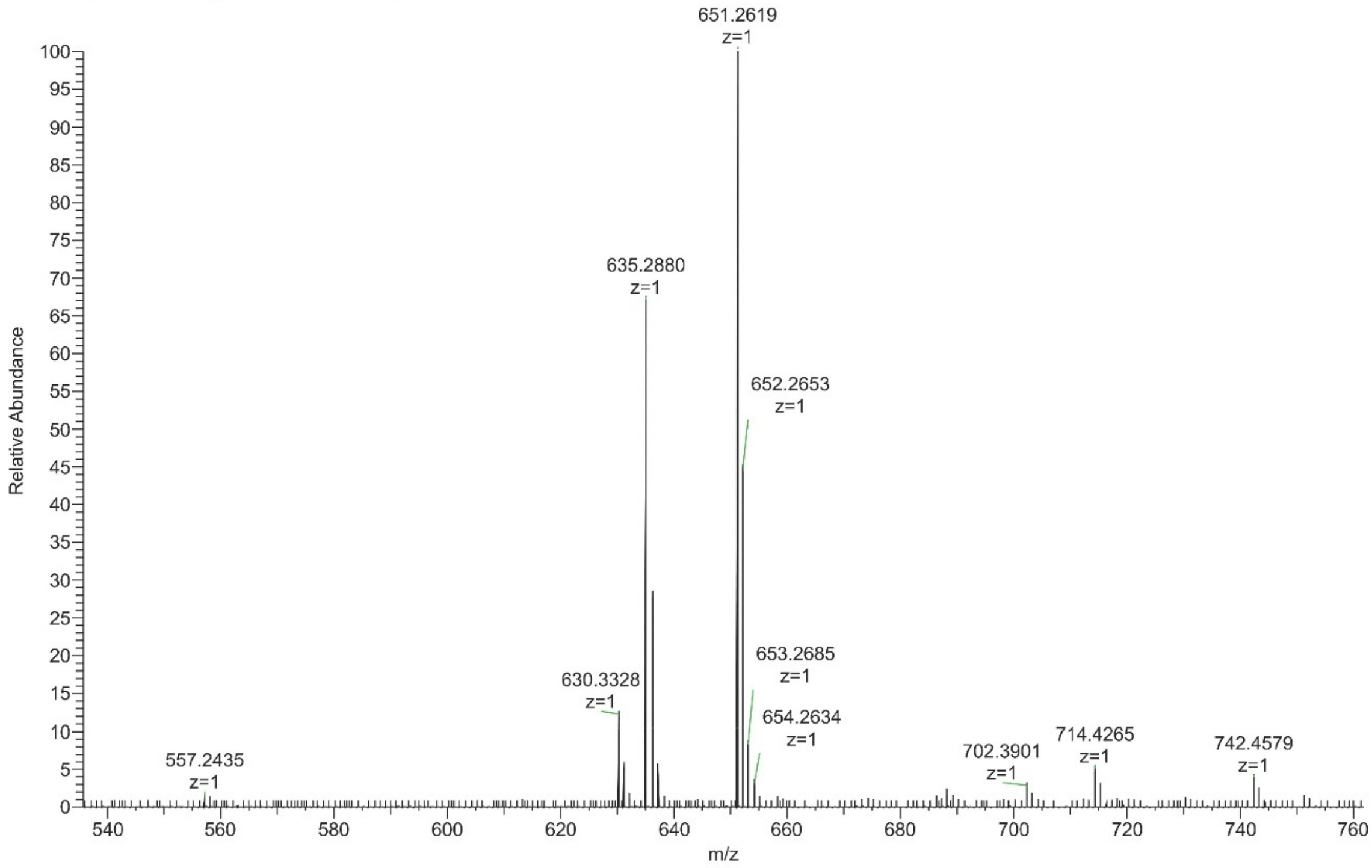

Mass spectrum of PhNHBoc-Py.

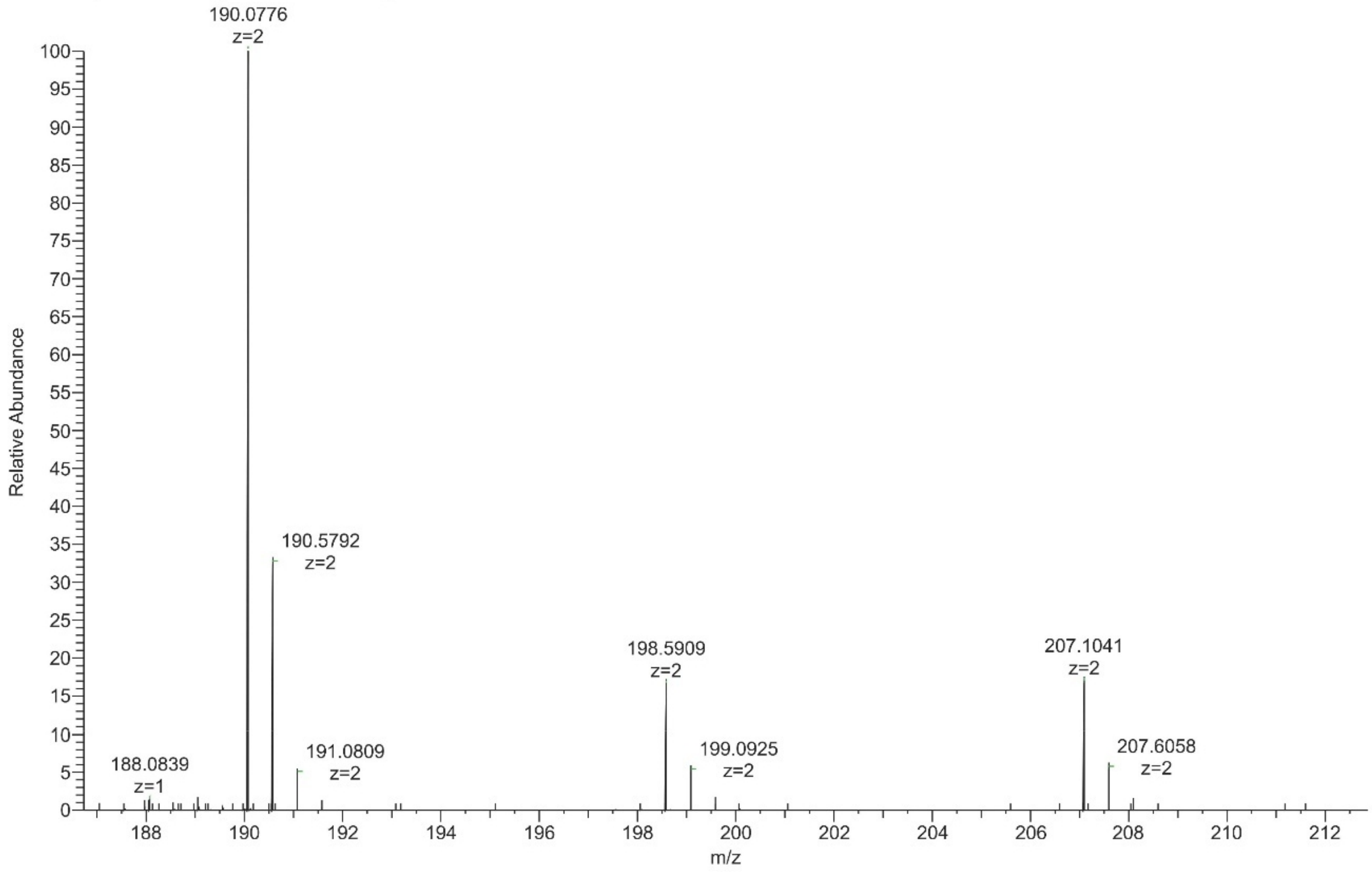

Mass spectrum of PhNH-Py.

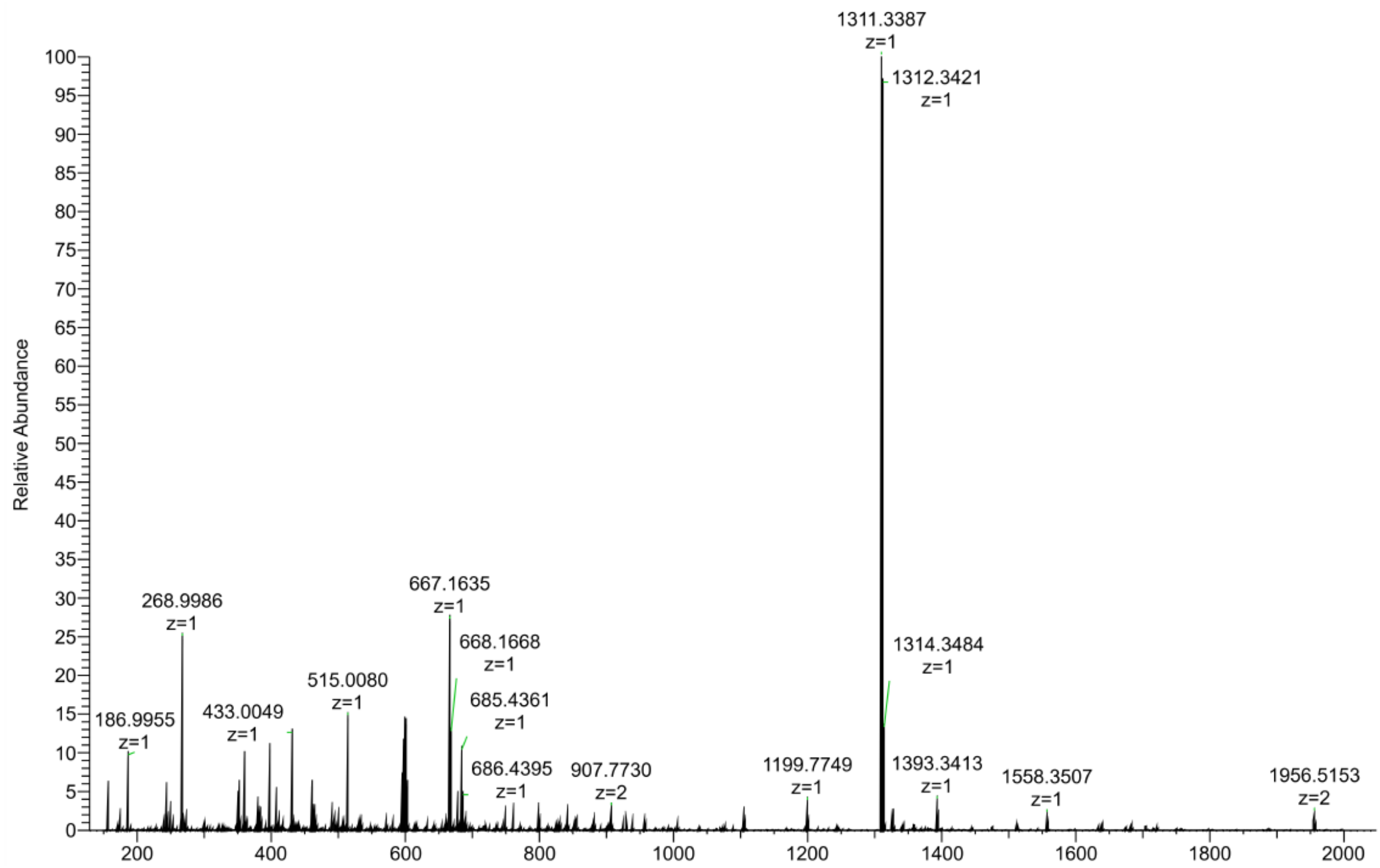

Mass spectrum of NDI-Ph-Py.
